# Donor–acceptor interactions between cyclic trinuclear pyridinate gold(i)-complexes and electron-poor guests: nature and energetics of guest-binding and templating on graphite†
†Electronic supplementary information (ESI) available: Synthetic manipulations, details on NMR titration experiments, stability study on **1b**, NMR and UV/vis absorption spectra, details on quantum chemical calculations and STM measurements. See DOI: 10.1039/c7sc05355j


**DOI:** 10.1039/c7sc05355j

**Published:** 2018-03-05

**Authors:** Raiko Hahn, Fabian Bohle, Stefan Kotte, Tristan J. Keller, Stefan-S. Jester, Andreas Hansen, Stefan Grimme, Birgit Esser

**Affiliations:** a Institute for Organic Chemistry , University of Freiburg , Albertstraße 21 , 79104 Freiburg , Germany . Email: besser@oc.uni-freiburg.de; b Mulliken Center for Theoretical Chemistry , University of Bonn , Beringstraße 4 , 53115 Bonn , Germany; c Kekulé Institute for Organic Chemistry and Biochemistry , University of Bonn , Gerhard-Domagk-Straße 1 , 53121 Bonn , Germany

## Abstract

Donor–acceptor-type interactions between π-electron systems are of high relevance in the design of chemical sensors. Due to their electron-rich nature, cyclic trinuclear complexes (CTCs) of gold(i) are ideal receptor sites for electron-deficient aromatic analytes. Scanning tunneling microscopy provided insight into the structures of two-dimensional crystals of pyridinate gold CTCs that form on a graphite template at the solid/liquid interface. One polymorph thereof – in turn – templated the on-top co-adsorption of π-acidic pyrazolate CTCs as electron-poor guests up to a certain threshold. From NMR titration experiments, we quantified free energies of –6.1 to –7.5 kcal mol^–1^ for the binding between pyridinate gold(i) CTCs and π-acidic pyrazolate CTCs. Quantum chemical calculations revealed that these interactions are largely dominated by London dispersion. These results give a more detailed insight into a rational design of sensitive CNT- or graphene-based sensors for π-acidic analytes, such as electron-deficient aromatics.

## Abstract 2

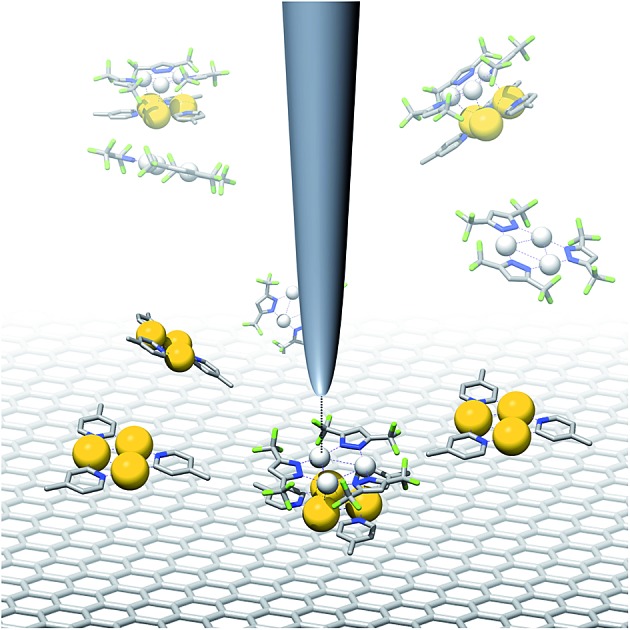
Aromatic stacking interactions of π-basic Au(i) complexes with π-acids were analyzed experimentally, theoretically and at the solid/liquid interface using STM.

## Introduction

Non-covalent interactions between π–systems and cations, anions or aromatics are found in biological systems and are important in, among others, host/guest chemistry, self-assembly,^1^–^3^ and molecular machines.^4^–^7^ Donor–acceptor-type interactions between planar arenes^8^ also play an important role in the design of chemical sensors, for instance for the detection of electron-poor nitroaromatics, such as 1,3,5-trinitrotoluene, TNT, or structurally related explosives.^9^ The field of resistivity-based sensing – using carbon nanotubes (CNTs) or graphene – is emerging due to the operational simplicity of such devices, their small size and potential low cost.^10^,^11^ In a CNT- or graphene-based sensor, however, binding of the analyte to the carbon surface is unspecific and often of insufficient strength.^11^–^13^ Aiming at the detection of electron-deficient aromatics, functionalization of the surface with a complementary electron-rich π-system could selectively template and increase the binding strength to a specific analyte compound or a group thereof. Non-covalent doping of the carbon-based material by adsorption of a monolayer-forming species is proposed to lead to changes in the electronic band structure. This may affect the electrical resistivity in a sensing setup – without requiring a covalent doping of the carbon material itself, which may introduce defects or disorder.^14^

**Fig. 1:**
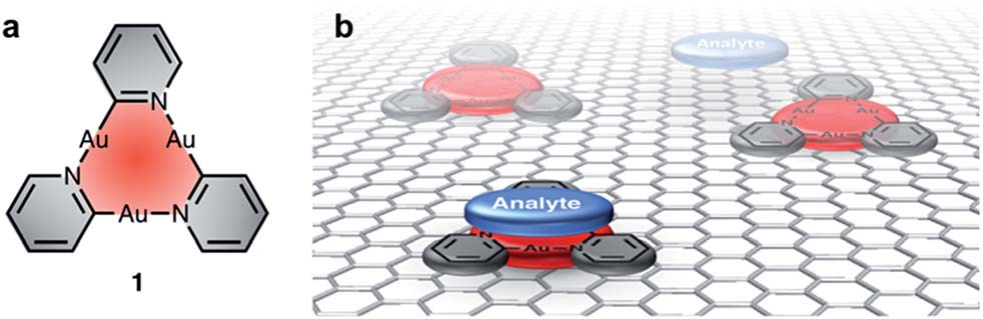
(a) Electron-rich cyclic trinuclear pyridinate gold(i) complexes **1** and (b) concept of functionalizing a CNT or graphene surface with **1** to template the binding of electron-poor analytes.

Due to their planar, cyclic structure and the electron-rich nature of their π-system, cyclic trinuclear complexes (CTCs) of gold(i)^15^–^17^ with pyridinate ligands (**1**, Fig. 1a) are promising candidates for the design of such graphene-based sensors. Our use of pyridine ligands with long alkyl substituents aimed at a two-dimensional (2D) supramolecular engineering approach towards packing of oblate CTCs on planar carbon-based materials, such as graphite or graphene.^18^ Au(i) CTCs have been shown to possess a high electron-density above and below the trinuclear gold core, also referred to as high π-basicity.^19^ Modification of a 2D carbon surface with complexes **1** could increase surface binding to electron-poor analytes, as depicted in Fig. 1b. The selective binding of an analyte on top of the active sites of the monolayer would effectively modulate charge transfer to the carbon surface, thereby leading to a change in electrical conductivity.

**Fig. 2:**
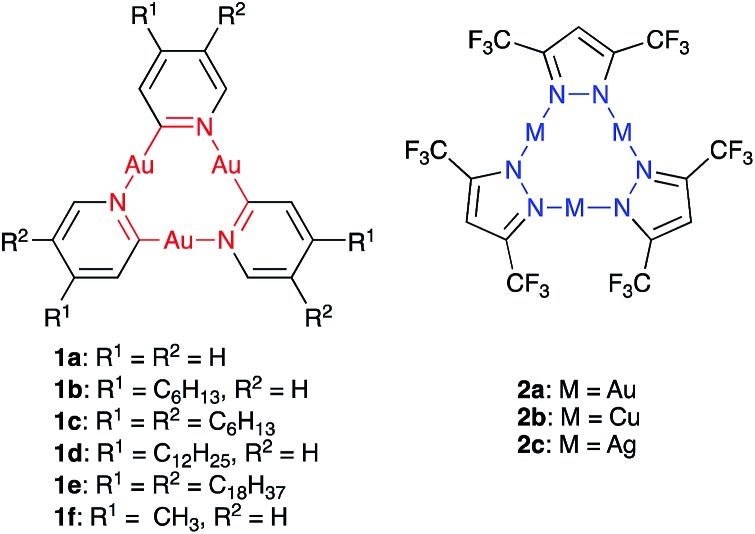
Electron-rich cyclic trinuclear pyridinate gold(i) complexes **1a–f** and electron-poor pyrazolate complexes **2a–c** used in this study.

Scanning tunneling microscopy (STM) is a tool to visualize receptor-analyte adducts that form or deposit on a solid surface. We used highly-oriented pyrolytic graphite (HOPG) as the bulk analogue of graphene and as model system for adsorption experiments by STM. Au(i)-CTCs have previously been investigated by this method after adsorption from various solvents.^20^ In addition, long alkyl/alkoxy side-chains are known to separate large macrocyclic backbones and determine the adsorption/packing into 2D crystalline supramolecular nanopatterns on graphite,^21^ a strategy that has previously been used for the design and high-resolution imaging of Au(i)-CTCs.^22^ A particular aim was to investigate whether monolayers of CTCs of type **1** could template the co-adsorption of electron-poor guests, which may be important for future sensing applications. To validate our concept, we chose electron-deficient guests with a known high π-acidity, namely pyrazolate CTCs **2a–c** (Fig. 2).^19^,^23^–^26^ While donor–acceptor-type interactions with electron-poor π-systems have not yet been reported for pyridinate CTCs of type **1**, they have been observed for imidazolate or carbeniate CTCs.^16^,^27^–^33^ However, attempts to quantify these interactions have not been reported up to date, and no account exists to study these interactions at the solid–liquid interface. We herein present a study on donor–acceptor-type interactions of pyridinate CTCs of type **1** with π-acidic guests **2**. We provide interaction free energies (determined through NMR-investigations and quantum chemical calculations for these compounds in solution) and visualize self-assembled monolayers of **1e** as well as co-adsorbates of **1e** and **2c** by STM at the solid/liquid interface. These results may aid the design of sensitive and selective sensors, where the graphite surface functionalized with CTCs **1** used in STM measurements is a model system for a chemoresistive CNT- or graphene-based sensor.

## Results and discussion

### Synthesis of pyridinate CTCs **1**

The pyridinate complex **1a** was the first CTC to be described by Vaughan in 1970,^34^ and its structure was confirmed through X-ray crystallography 30 years later by Balch and coworkers.^35^ In order to obtain derivatives of CTCs **1** with the ability to form stable 2D self-assemblies on HOPG for STM studies and with high solubility in organic solvents for NMR titration experiments, one (**1b**, **d**) or two (**1c**, **e**) alkyl chains of different lengths were attached to each pyridine ring.

**Scheme 1:**
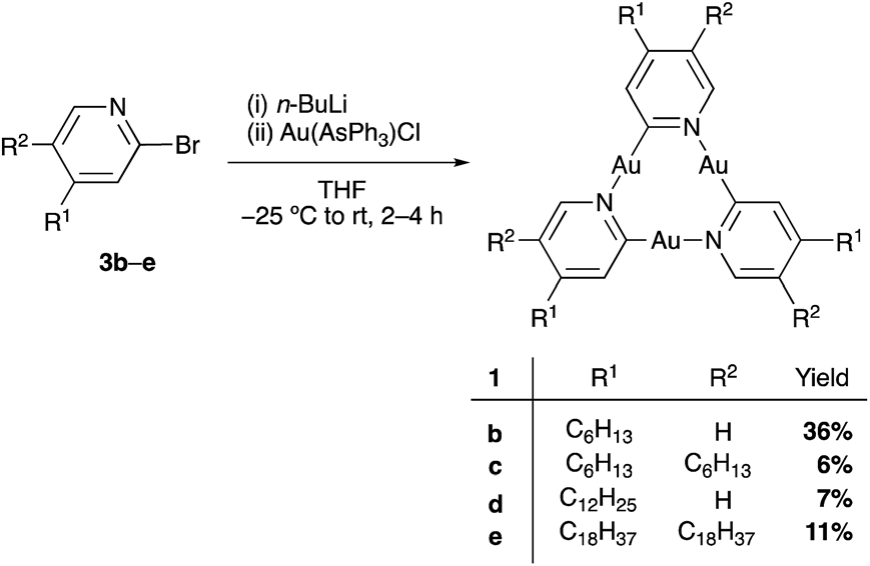
Synthesis of the trinuclear pyridinate gold complexes **1b–e**.

Starting from the 2-bromo pyridines **3b–e** (for synthesis see ESI†), the synthesis of CTCs **1b–e** was accomplished using a modification of Vaughan's procedure (Scheme 1).^34^ Several column chromatography and recrystallization steps led to pure samples of **1b**, **d**, and **e**. Pyridinate CTC **1c** with six hexyl chains, however, could not be obtained in pure form (see ESI†). **1b** and **1d** showed excellent solubility in common organic solvents, and **1e** became soluble at temperatures above 40 °C. This allowed for their characterization by NMR spectroscopy and MALDI-TOF analysis. All synthesized pyridinate CTCs **1** were somewhat light-sensitive but did not decompose in high vacuum. Compared to the unsubstituted derivative **1a**,^34^**1b**, **1d**, and **1e** were less prone to decomposition in solution and could be handled for a prolonged amount of time, likely due to the shielding effect of the alkyl chains on the trinuclear gold centers. Pyrazolate CTCs **2a–c** were synthesized according to literature procedures^23^,^36^ and were obtained in excellent purity. They showed high solubility in benzene, but were somewhat light-sensitive.

### Determination of binding constants between CTCs **1** and **2a–c**

The binding constants between the pyridinate CTCs **1b** and **1d** as π-donors (D) and electron-deficient CTCs **2a–c** as π-acceptors (A) were determined using ^1^H NMR titration experiments.^37^ UV/vis spectra of mixtures of **1d** and acceptors **2a–c** showed the appearance of a small charge-transfer band between 250 and 350 nm (see ESI†). However, due to significant overlap of the absorption maxima of the two compounds UV/vis spectroscopy was not employed to determine binding constants.

**Scheme 2:**
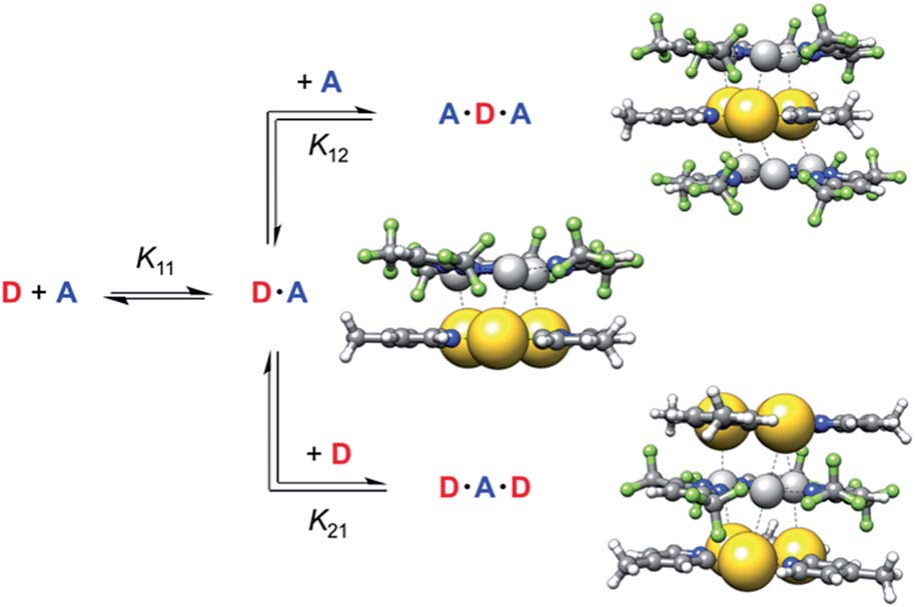
Equilibria of complex formation between gold pyridinate CTC **1f** as donor D and silver pyrazolate **2c** as acceptor A (PBEh-3c calculated structures).

**Fig. 3:**
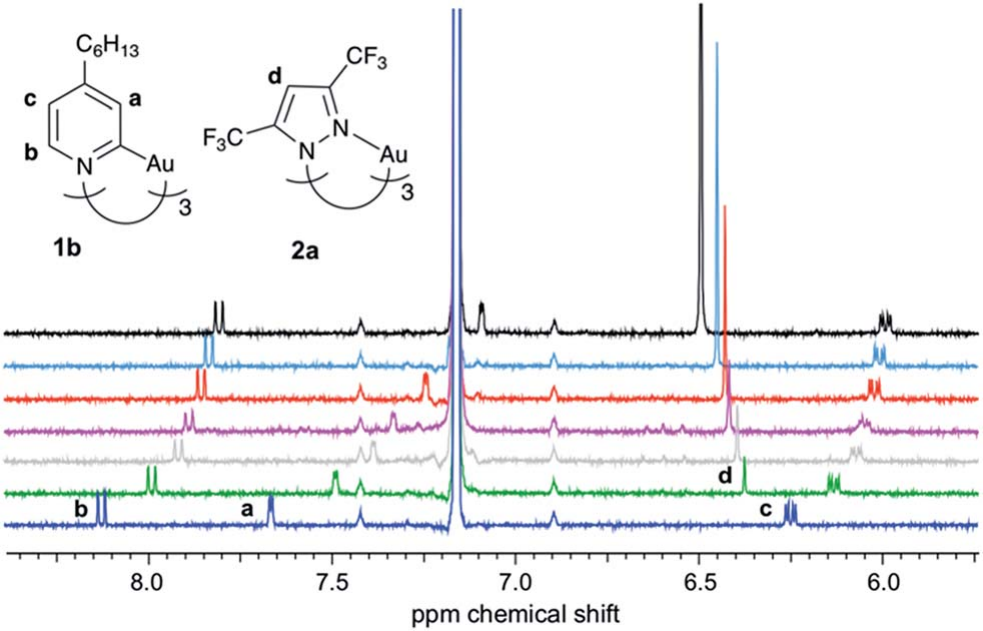
^1^H NMR titration of **1b** with **2a** (C_6_D_6_, 300 K; added equivalents of **2a** from bottom to top: 0, 0.6, 1.1, 1.5, 2.5, 4.0, 11).

Compared to common host/guest systems, a quantitative analysis of π–π interactions in the case of CTCs is more difficult to achieve, since due to the planarity of the molecules both the donor and the acceptor possess two equal binding sites. Hence not only the formation of 1 : 1, but also of 2 : 1 and 1 : 2 complexes and higher order structures is possible (Scheme 2). In the NMR titrations reported herein, the acceptor was added to the donor, and in most data points collected the acceptor was present in excess. Hence the formation of 1 : 1 (D·A) and 1 : 2 (A·D·A) complexes was considered, while 2 : 1 (D·A·D) complexes were neglected. The results of the titration experiments were fitted using non-linear regression to a 1 : 2 (D : A) binding model,^37^ employing an online tool provided by Thordarson and coworkers.^38^,^39^ All measurements showed a fast exchange between donor and acceptor in relation to the NMR time scale, resulting in one set of signals for the donor and for the acceptor. Selected ^1^H NMR spectra from a titration of **1b** with **2a** are shown in Fig. 3. Titration experiments of CTC **1b** with the three acceptors **2a–c**, performed three times at slightly different concentrations (see ESI†), yielded the binding constants and free energies of binding listed in Table 1. These values confirm that the π-acidity of the pyrazolate CTCs **2** increases in the order Au < Cu < Ag, as predicted by Omary *et al.*^23^

**Table 1:** Experimental binding constants *K* (in L mol^–1^) and free binding energies Δ*G* (in kcal mol^–1^) from ^1^H NMR spectroscopic titrations in C_6_D_6_ at 300 K of pyridinate CTC **1b** (as donor D) with pyrazolate CTCs **2a–c** (as acceptors A)

Acceptor	*K*_11_ ^*a*^	Δ*G*_11_^*a*^	*K*_12_ ^*b*^	Δ*G*_12_^*b*^
**2a** (M = Au)	3.01 × 10^4^	–6.1	515	–3.7
**2b** (M = Cu)	1.57 × 10^5^	–7.1	260	–3.3
**2c** (M = Ag)	2.83 × 10^5^	–7.5	3090	–4.7

^*a*^1 : 1 complex D·A.

^*b*^1 : 2 complex A·D·A.

The binding constants *K*_11_ for the formation of D·A complexes lie between 3 × 10^5^ and 3 × 10^4^ L mol^–1^, corresponding to free binding energies Δ*G*_11_ between –6.1 and –7.5 kcal mol^–1^. These values are in the typical range of binding free energies for common supramolecular complexes.^6^,^8^,^40^ For comparison, the mean of all experimental free energies of the S30L^41^ (benchmark set of realistic host-guest complexes) amounts to –6.5 kcal mol^–1^. This demonstrates that pyridinate CTCs **1** are promising candidates to strongly bind electron-poor analytes.

Due to a lowering of the electron density of the donor in the D·A complex, the binding constants *K*_12_ for the formation of complexes A·D·A are significantly smaller than *K*_11_. This indicates an anti-cooperative binding, since a ratio of 4 : 1 between *K*_11_ and *K*_12_ would be expected in the statistical case.^37^

All binding isotherms were also fitted to a 1 : 1 model, which clearly showed its inferiority over the 1 : 2 model. The deviations between experimental and fitted values (so-called residuals) were abound ten times higher than for the 1 : 2 model and showed sinusoidal shape (see ESI†).^42^ Binding models corresponding to higher-order structures were not employed, since *K*_12_ was already found to be two or three orders of magnitude smaller than *K*_11_.

A comparison with the binding constants determined for acceptor **2c** with donor **1d**, carrying three dodecyl groups, of *K*_11_ = 2.81 × 10^5^ L mol^–1^ and *K*_12_ = 9440 L mol^–1^ showed that the length of the alkyl groups on **1** did not affect its binding ability. This was important with respect to the STM measurements described below, where pyridinate complex **1e** with octadecyl groups was used.

Quantum chemical calculations, employing an established multilevel Ansatz,^40^,^43^ provided association free energies for the 1 : 1 complexes of **1b** with **2a–c** (Table 2). DFT calculations on gold CTCs have been performed before, however, with the intention to investigate their self-assembly on HOPG.^20^ For computational efficiency the alkyl side chains of donor **1b** were replaced by methyl groups (**1f**, Fig. 2). Geometries were optimized with the DFT composite scheme PBEh-3c,^44^ and energies (Δ*E*) were refined with PBE0-D3^45^,^46^ as a dispersion-corrected hybrid functional using the large def2-QZVP-f/-g^47^ basis set. Thermostatistical contributions to free energies Δ*G*_RRHO_ were evaluated at the PBEh-3c level of theory. COSMO-RS^48^ provided solvation free energies (Δ*δG*_solv_) in a black-box fashion.

**Table 2:** Calculated binding free energies^*a*^ Δ*G*_calc 11_^*b*^ of pyridinate CTC **1f** (as donor D) with pyrazolate CTCs **2a–c** (as acceptors A)

Acceptor	Δ*E* (PBE0-D3/QZ-f/-g)	Δ*G*_RRHO_ (PBEh-3c)	Δ*δG*_solv_	Δ*G*_calc 11_
**2a** (M = Au)	–32.1	17.4	4.6	–10.1
**2b** (M = Cu)	–38.0	17.5	6.1	–14.3
**2c** (M = Ag)	–39.5	17.3	6.0	–16.3

^*a*^All values are given in kcal mol^–1^.

^*b*^Obtained as the sum of electronic interaction energies Δ*E*, thermostatistical contributions Δ*G*_RRHO_ and solvation free energies Δ*δG*_solv_.

For the binding free energies between pyridinate gold CTC **1f** and pyrazolate CTCs **2a–c**, a reasonable agreement of (shifted) theoretical and experimental Δ*G*_calc 11_ values was observed. The experimental trend of increasing acceptor strength in going from **2a** to **2c** was clearly reproduced. These stacked CTCs are to a large extent stabilized by London dispersion. For the complex of **1f·2c** (without alkyl side chains) the dispersion interaction energy (D3(PBE0) level) amounted to –31.3 kcal mol^–1^, which accounts for 79% of the total interaction energy Δ*E*. The same trend was obtained with our recently developed D4 dispersion model,^49^ which additionally takes into account the influence of the electronic structure on the dynamic polarizability and thus the dispersion energy (see ESI†).

To further validate the DFT-calculated interaction energies, a high-level coupled-cluster value employing DLPNO-CCSD(T)^50^,^51^/TightPNO^52^/CBS^53^(def2-TZVPP^47^/def2-QZVPP^54^) for the 1 : 1 complex between **1f** and **2c** was calculated, yielding an electronic gas phase interaction energy of Δ*E*_11_ = –39.2 (±3) kcal mol^–1^. This value is in very good agreement with the corresponding PBE0-D3/def2-QZVP-f/-g result of –39.5 kcal mol^–1^, including 6.8 kcal mol^–1^ deformation energy mostly originating from the geometry relaxation of **2c**. Tentatively, the deviation to the experiment can be attributed to the neglect of the alkyl side chains on the donor **1f** and/or the implicit solvation model, which may be improperly parameterized for coinage metal cations within these planar structures.^55^

### Investigation of donor–acceptor-type interactions using STM

Finally, the interaction between a pyridinate gold CTC of type **1** and a pyrazolate acceptor of type **2** was investigated at the solid/liquid interface using STM. The HOPG surface is an excellent model system for graphene-based systems, such as in the proposed sensors. Our study aimed at elucidating whether this surface could be functionalized with electron-rich CTCs **1**. Up to date only two reports of STM measurements on CTCs have been published on pyrazolate and carbeniate complexes on HOPG.^20^,^22^

#### Self-assembly of **1e** on HOPG

**Fig. 4:**
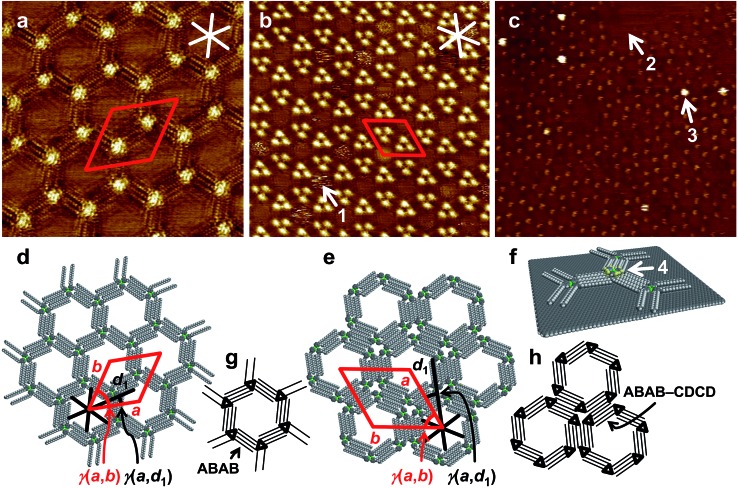
(a)–(c) STM images, (d)–(f) supramolecular models, and (g)–(h) schematic models of self-assembled ad-layers. (a), (d), (g): Low-concentration polymorph (pol. A) of **1e** (a: *c* = 3 × 10^–6^ M, 20 × 20 nm^2^, *V*_S_ = –0.15 V, *I*_t_ = 24 pA; *a* = *b* = (5.6 ± 0.2) nm, *γ*(*a*,*b*) = (60 ± 2)°, *γ*(*a*,*d*_1_) = (15 ± 1)°); (b), (e), (h) high-concentration polymorph (pol. B) of **1e** (b: *c* = 10^–5^ M, 42.8 × 42.8 nm^2^, *V*_S_ = –0.8 V, *I*_t_ = 10 pA; *a* = *b* = (7.1 ± 0.2) nm, *γ*(*a*,*b*) = (60 ± 2)°, *γ*(*a*,*d*_1_) = (19 ± 1)°; both samples were thermally annealed for 20 s at 80 °C prior to imaging); (c), (f): nanopattern of **1e** (0.5 μL of a 3 × 10^–6^ M solution, thermally annealed for 20 s at 80 °C) to which (after cooling to r.t.) 0.5 μL of a 10^–3^ M solution of **2c** were added (75 × 75 nm^2^ (internal scanner calibration), *V*_S_ = –0.9 V, *I*_t_ = 18 pA). White (black) and red lines indicate HOPG main axis directions and unit cell vectors, respectively.

We used **1e** equipped with (long) octadecyl chains to allow for the formation of stable self-assembled monolayers at the interface of 1-phenyloctane (PHO) and HOPG. At a (starting) concentration of 3 × 10^–6^ M of **1e** in the supernatant solution, a porous network (polymorph A, Fig. 4a and d) was observed, to which lattice constants of *a* = *b* = (5.6 ± 0.2) nm, *γ*(*a*,*b*) = (60 ± 2)°, containing two molecules, were indexed. The alkyl side chains aligned along the HOPG main axis directions, so that the unit cell vector *a* was oriented at an angle of *γ*(*a*,*d*_1_) = (15 ± 1)° relative to the HOPG main axis direction *d*_1_. This showed that HOPG acted as a template for the network formation. All side chains of the *D*_3h_ symmetric molecules of **1e** interdigitated in an intermolecular ABAB fashion (Fig. 4g), resulting in a chiral honeycomb network. Such porous patterns have previously been reported for alkyl/alkoxy-substituted hexadehydrotribenzo[12]annulenes (DBAs),^56^ to which **1a–f** are similar with respect to backbone size/symmetry and substitution pattern. However, the chiral honeycomb patterns were only observed for the DBAs with a shorter chain length of C_10_H_21_ (with respect to **1e**), whereas the DBA with identical chain length (C_18_H_37_) formed a linear-type network (in different solvents). As can be seen in Fig. 4d and g, all innermost side chains are oriented in a clockwise manner. In the packing of **1e** (polymorph A), each CTC (A) is coordinated (*via* alkyl side chain interdigitation) to three adjacent CTCs (B) that are rotated by 60°/180°/300°, a phenomenon related to the interdigitation of the long side chains. In contrast, CTCs with short alkyl side chains pack rather densely and are all oriented in the same direction (although an inverted orientation relative to its neighbors has been discussed).^20^

Concentration-20,22 and solvent-dependent^56^ polymorphisms are well-known phenomena.

Here, at an increased concentration for **1e** of 10^–5^ M, the molecules assembled in the more densely packed polymorph B (Fig. 4b and e). Two out of six alkyl chains of each molecule were not adsorbed on the HOPG surface but pointed towards the solution phase. Hexagonal assemblies resulted, likely with the same chiral packing as described in Fig. 4d and g, but with a counterclockwise orientation (Fig. 4h). Each corner of the hexagonal units appeared as a bright spot, representing the molecular backbones. The hexagonal assemblies packed densely to form an – again – hexagonal pattern (Fig. 4e and h), to which a unit cell of *a* = *b* = (7.1 ± 0.2) nm, *γ*(*a*,*b*) = (60 ± 2)° was indexed. The unit cell vector *a* was oriented at an angle *γ*(*a*,*d*_1_) = (19 ± 2)° relative to the HOPG main axis direction *d*_1_. Each (indexed) unit cell contained six molecules of **1e**. As a result of the hierarchical packing of hexagonal units (each formed by six molecules), triples of bright spots, containing three neighboring backbones, were observed. The bright regions in the interior of some of the pores (indicated by arrow 1 in Fig. 4b) appeared blurred, which was attributed to unspecifically adsorbed molecules that most probably moved/rotated faster than the timescale of the measurement. Similar studies on pore filling have been performed before with pyrene as guest molecule^20^ and have also been extended to larger guest molecules.^57^ Even at this increased concentration, no evidence for the formation of multilayers, where molecules of **1e** stacked on top of each other, was observed in any of the images acquired. This absence of multilayer stacks of **1e** on HOPG even at increased concentrations was highly relevant for being able to correctly conclude – from STM topography data – a possible formation/adsorption of mixed stacks.

#### Co-adsorption of **1e** and **2c** on HOPG

We aimed at the information whether **1e** on HOPG could act as a template to allow for a specific adsorption of electron-deficient CTCs **2**. We used silver pyrazolate **2c**, which had shown the highest binding free energies to CTCs **1**. In addition, **2c** does not form self-assembled monolayers at the PHO/HOPG interface (see ESI†). The lower concentration of 10^–6^ M for **1e** (for which polymorph A was observed) was chosen to guarantee that all alkyl chains were aligned on the HOPG substrate (which otherwise might sterically hinder a double layer formation). 0.5 μL of a 3 × 10^–6^ M solution of **1e** in PHO were applied onto HOPG at 80 °C, the surface was allowed to cool to room temperature, and successful monolayer formation of polymorph A was confirmed *via* STM. Then, 0.5 μL of a 10^–3^ M solution of **2c** in PHO were added, and STM imaging was continued. The resulting image (Fig. 4c) shows the hexagonal packing of **1e** in polymorph A with parts of the HOPG surface remaining uncovered (as indicated by arrow 2). Additional bright features, such as indicated by arrow 3, are visible, which are clearly localized above the backbone regions of **1e**, according to the lattice. We attribute these to the formation of supramolecular stacks, where **2c** adsorbs on top of the backbone of **1e**, as indicated by arrow 4 in Fig. 4f. Through its electron-rich trinuclear core, **1e** acted as a secondary template for the adsorption of electron-poor, π-acidic **2c**, dictated through donor–acceptor interactions. Judging from the determined association constants (Table 1) a higher coverage of gold pyridinate CTCs **1e** with silver pyrazolate CTCs **2c** would have been expected at this concentration. To elucidate a potential charge-transfer from the gold-pyridinate-complex to the graphite surface, which would diminish its donor strength, we calculated the charge transfer from CTC **1f** onto a molecular extended graphite surface in the gas phase (see ESI†). We found, however, the charge-transfer to be negligibly small.

This result shows that gold pyridinate CTCs **1** are promising molecules in the design of CNT- or graphene-based sensors for electron-poor aromatics, where surface-functionalization with **1** would increase binding of the analyte. Furthermore, to the best of our knowledge, this is the first report of a co-adsorption, studied by STM, which is directed by a donor–acceptor-type interaction between a π-basic and a π-acidic molecule. Several studies have been published, where the pores of self-assembled 2D-networks on HOPG were filled with guest molecules^58^–^64^ or where the adlayer of one macrocycle was used as a template for another macrocycle.^65^ However, donor–acceptor-type interactions in the context of the formation of a double layer at the solid/liquid interface have only been discussed for STM-observations of the co-deposition of C_60_ with thiophene-containing macrocycles.^66^,^67^ The results discussed herein will stimulate us to (in the future) enhance the π-basicity of donor compounds on HOPG and to quantify host-guest binding on the surface.

#### Self-assembly of **1e** on gold(111)

We were also interested to find out whether **1e** would form self-assembled monolayers on Au(111). As shown in the ESI,† a similar network to the one found on HOPG was formed on Au(111). To the best of our knowledge, this is the first report on STM images of a gold coordination compound that self-assembles at the Au(111) surface.

## Conclusions

In conclusion, we have shown through NMR titration experiments that the free energies of binding between gold(i) pyridinate CTCs **1** and coinage-metal pyrazolate CTCs **2** lie between –6.1 and –7.5 kcal mol^–1^. This places them in the typical range compared to common supramolecular host-guest complexes, where the host possesses a cavity. High-level quantum chemical calculations revealed that these interactions are largely stabilized by London dispersion. Their strongly π-basic nature makes pyridinate CTCs **1** attractive as binding sites for electron-poor molecules, for instance in the design of chemical sensors. We demonstrated this concept by using pyridinate CTC **1e** to template the co-adsorption of silver(i) pyrazolate CTC **2c** on HOPG. These results give a more detailed insight into a rational design of sensitive CNT- or graphene-based sensors for π-acidic analytes, such as electron-deficient aromatics, with potential for a better sensitivity for π-acidic analytes as compared to existing detection systems.

## Conflicts of interest

There are no conflicts to declare.

## Supplementary Material

Supplementary information
