# Supplementary material for: Donor–acceptor interactions between cyclic trinuclear pyridinate gold(i)-complexes and electron-poor guests: nature and energetics of guest-binding and templating on graphite
Source: Chem Sci. 2018 Mar 5;9(14):3477–83. doi: 10.1039/c7sc05355j (PMC5934696; doi:10.1039/c7sc05355j)
Supplement: Supplementary file 1 [file SC-009-C7SC05355J-s001.pdf]

Electronic Supplementary Material (ESI) for Chemical Science.
This journal is © The Royal Society of Chemistry 2018

Electronic Supplementary Information for

Donor-acceptor interactions between cyclic trinuclear pyridinate gold(I)-complexes and electron-poor guests: Nature, energetics and templating of guest-binding on graphite

Raiko Hahn,[a] Fabian Bohle,[b] Stefan Kotte,[c] Tristan J. Keller,[c] Stefan-S. Jester,[c] Andreas Hansen,[b] Stefan Grimme[b] and Birgit Esser*[a]

[a] Institute for Organic Chemistry, University of Freiburg, Albertstraße 21, 79104 Freiburg, Germany. E-mail: besser@oc.uni-freiburg.de

[b] Mulliken Center for Theoretical Chemistry, University of Bonn, Beringstraße 4, 53115 Bonn, Germany

[c] Kekulé Institute for Organic Chemistry and Biochemistry, University of Bonn, Gerhard-Domagk-Straße 1, 53121 Bonn, Germany

Table of contents

1 Materials and methods S2
2 Synthetic manipulations S4
3 NMR titration experiments S14
4 Stability of pyridinate **1b** S38
5 NMR spectra S39
6 UV/Vis spectroscopy S53
7 Computational studies S55
8 STM measurements S81
9 References S88

1 Materials and methods

Chemicals and solvents were purchased from Sigma-Aldrich, Acros-Organics, Alfa-Aesar, TCI, Merck, or ChemPur and used directly unless otherwise noted. Reactions with dry solvents were carried out under an argon atmosphere in oven-dried glassware. Anhydrous solvents (THF, CH_2Cl_2) were withdrawn from an MB-SPS-800 unit from M-Braun and stored over molecular sieves (4 Å). Solvents for column chromatography were purified by distillation. 2-(Dimethylamino)-ethanol was distilled under argon and stored over molecular sieves (4 Å). **Analytical Thin Layer Chromatography (TLC)** was carried out using TLC Silica gel 60 F_{254} aluminum sheets from Merck KGaA or POLYGRAM® Alox N/UV_{254} polyester sheets from Macherey-Nagel GmbH & Co. KG. Detection was carried out using short wave UV light (254 nm and 366 nm). **Column chromatography** was accomplished using silica gel S (0.040-0.062 mm) or aluminum oxide purchased from Merck KGaA and deactivated with 15% w/w H_2O. **^{1}H and ^{13}C NMR** spectra were recorded at room temperature on the following spectrometers: Bruker Avance DPX 500 (^{1}H = 500 MHz, ^{13}C = 126 MHz), Bruker Avance DPX 400 (^{1}H = 400 MHz, ^{13}C = 101 MHz), Bruker Avance DPX 300 (^{1}H = 300 MHz, ^{13}C = 76 MHz) (all University of Bonn), Bruker Avance III HD 500 (^{1}H = 500 MHz, ^{13}C = 126 MHz) and Bruker Avance II 400 (^{1}H = 400 MHz, ^{13}C = 101 MHz) (University of Freiburg, 300 K). Unless stated otherwise all spectra were recorded in deuterochloroform or benzene-*d*6, which were purchased from Sigma-Aldrich, Acros-Organics or Deutero, and all chemical shifts are given in δ units relative to tetramethylsilane (singlet: δ = 0.00). Calibration of ^{1}H spectra was achieved using the residual solvent signal of chloroform at δ_H = 7.26 ppm or benzene at δ_H = 7.16 ppm. ^{13}C spectra were calibrated to the central signal line of the triplet of chloroform at δ_C = 77.16 ppm or the singlet of benzene at δ_C = 128.06 ppm.[1] Analyses followed first order, and the following abbreviations were used throughout: s = singlet, d = doublet, t = triplet, dd = doublet of doublets, m = multiplet. Coupling constants (*J*) are given in Hertz [Hz]. **Mass spectra (MS)** were recorded on a Bruker Daltonics ESI micrOTOF-Q time of flight, a Thermo Finnigan MAT 95 XL sector mass, a Bruker Daltonics autoflex II TOF/TOF spectrometer (University of Bonn), a Thermo Exactive with Orbitrap-analyser and electrospray ionization (ESI) or a MALDI-TOF autoflex™ by Bruker (University of Freiburg). **Melting points** were determined using a BÜCHI 510 melting point (University of Bonn). **UV/Vis spectra** were recorded on a Perkin-Elmer Lambda 950 UV/VIS spectrometer.

The **titration of alkyl magnesium halide** or alkyl magnesium halide-lithium chloride suspensions was performed following a procedure by Peters *et al.*[2] A stock solution containing *N*-phenyl-4-phenylazoaniline (10 mg, 37 µmol) in dry and degassed toluene (8.0 mL) and 2-butanol (0.80 mL) was prepared and stored under argon over 3 Å molecular sieves (stable over month). The Grignard suspension was added dropwise under inert conditions to the stock solution (0.2–0.4 mL). The equivalence point was indicated by a color change from orange to red. The procedure was performed twice, carried out before every

use, and the calculated concentration of 2-butanol was used to determine the concentration of the Grignard suspension.

Pyrazolate CTCs 2 were synthesized following procedures of Omary *et al.* (**2a**)[3] and Dias *et al.* (**2b**,**c**).[4]

Chloro(triphenylarsine)gold(I) was synthezised following a procedure of Nieto-Oberhuber *et al.*.[5]

2 Synthetic manipulations

2.1 Preparation of Grignard reagents

2.1.1 Preparation of alkyl magnesium halide suspensions

The preparation of alkyl magnesium halide solutions was performed following a modified procedure by Peters *et al.*[2] A dry three-necked round-bottom flask was charged with magnesium turnings (1.2 eq.), equipped with a reflux condenser and heated *in vacuo* for 7–10 min. After cooling to room temperature, 1/3 of a total amount of dry and degassed THF (1.7 mmol/mL) was added, and the resulting suspension was sonicated for 10 min. The respective alkyl halide (1.0 eq.) and a small crystal of iodine were added. The reaction mixture was heated to 85 °C without stirring to initiate activation of the Grignard formation. As soon as the formation of the Grignard reagent had started, the remaining THF was added, and the reaction mixture was heated under reflux for 1 h. The suspension was allowed to cool to room temperature and stirred overnight. The resulting Grignard suspension was used without purification and titrated as described above.

2.1.2 Preparation of alkyl magnesium halide-lithium chloride suspensions

The preparation of the alkyl magnesium halide-lithium chloride suspensions was performed following a modified procedure by Peters *et al.*.[2] To ground and vacuum-dried (120 °C, 8 h, 10^{-3} mbar) LiCl (1.0 eq.) was added the respective alkyl magnesium halide suspension in THF (1.0 eq.) under inert conditions. The reaction mixture was stirred at room temperature for 20 h until all solids had dissolved. It was then titrated and used without purification.

2.2 Synthesis of hexylpyridines 6b and 6c

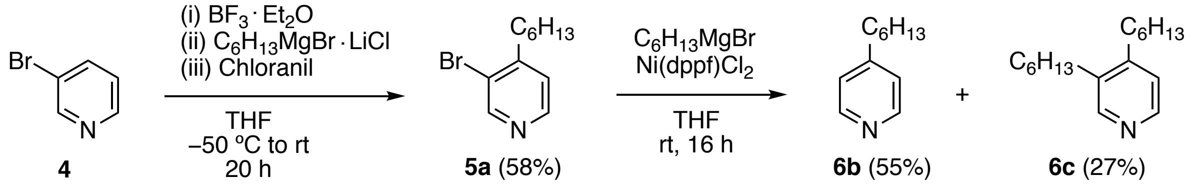

2.2.1 3-Bromo-4-hexylpyridine (5a)

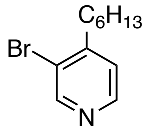

5a

The synthesis of **5a** was performed in analogy to a procedure by Knochel and coworkers.[6] To a stirred solution of 3-bromopyridine (**4**, 0.60 mL, 970 mg, 6.1 mmol, 1.0 eq) in dry THF (31 mL) was added $BF_3 \bullet OEt_2$ (0.86 mL, 960 mg, 6.8 mmol, 1.1 eq) at 0 °C. The solution was stirred at 0 °C for 20–30 min and cooled to −30 to −50 °C. $LiCl \bullet BrMgC_6H_{13}$ (1.4 M suspension in THF, 8.5 mL, 12 mmol, 2.0 eq.) was added over a period of 10–20 min. The orange suspension was

stirred at ambient temperature for 3 h. Chloranil (3.0 g, 12 mmol, 2.0 eq.) was suspended in dry THF (10 mL) and added to the reaction mixture at −30 °C. The resulting green suspension was allowed to warm to room temperature and stirred overnight. The resulting yellow suspension was quenched with NH_3 (9 mL, 25% w/w in H_2O) and brine (90 mL) and extracted with Et_2O (4 × 60 mL). The combined organic extracts were concentrated *in vacuo*. The residue was dissolved in CH_2Cl_2 (40 mL), washed with brine (20 mL) and dried over Na_2SO_4. Two consecutive chromatography steps (silica gel, CH_2Cl_2 and silica gel, cyclohexane/EtOAc: 15/1) yielded **5a** (870 mg, 3.6 mmol, 58%) as a yellow liquid. R_f 0.50 (cyclohexane/EtOAc: 4/1); ^{1}H NMR (400 MHz, $CDCl_3$): δ = 8.64 (s, 1H), 8.39 (d, *J* = 4.9 Hz, 1H), 7.14 (d, *J* = 4.9 Hz, 1H), 2.71 (t, *J* = 7.8 Hz, 2H), 1.65–1.58 (m, 2H), 1.44–1.25 (m, 6H), 0.90–0.86 (m, 3H)); ^{13}C NMR (101 MHz, $CDCl_3$): δ = 152.0, 151.0, 148.3, 125.2, 123.3, 35.5, 31.7, 29.1, 29.0, 22.7, 14.2; HRMS (ESI+): *m/z* calcd for $C_{23}H_{40}BrN$ 242.0539 $[M+H]^+$, found 242.0537.

2.2.2 4-Hexylpyridine (6b) and 3,4-dihexylpyridine (6c)

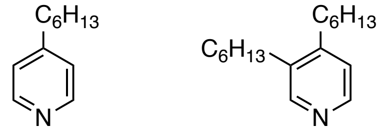

6b **6c**

The synthesis of **6b** and **6c** was performed following a modified procedure by Kumada and coworkers.[7] To a stirred solution of 3-bromo-4-hexylpyridine (**5a**, 460 mg, 1.9 mmol, 1.0 eq.) and Ni(dppp)Cl_2 (52 mg, 130 µmol, 0.07 eq.) in dry THF (23 mL) was added $C_6H_{13}MgBr$ (1.44 M suspension in THF, 1.7 mL, 2.5 mmol, 1.3 eq.) at room temperature. After stirring overnight, another portion of $C_6H_{13}MgBr$ (1.44 M solution in THF, 0.20 mL, 0.29 mmol, 0.15 eq.) was added to the dark suspension. The reaction mixture was stirred for 2.5 h and quenched with HCl (8.0 mL, 10% w/w in H_2O), after TLC indicated complete consumption of the starting material **5a**. A saturated aqueous solution of $NaHCO_3$ (20 mL) was slowly added, and the mixture was extracted with Et_2O (50 mL) and EtOAc (3 × 15 mL). The combined organic extracts were washed with brine and dried over Na_2SO_4. Purification by column chromatography (silica gel, cyclohexane/EtOAc: 10/1) yielded **6b** (170 mg, 1.0 mmol, 55%) and **6c** (130 mg, 0.52 mmol, 27%) as colorless liquids.

6b: R_f 0.23 (cyclohexane/EtOAc: 5/2); ^{1}H NMR (400 MHz, $CDCl_3$): δ = 8.47 (dd, *J* = 4.5, 1.5 Hz, 2H), 7.09 (dd, *J* = 4.5, 1.5 Hz, 2H), 2.59 (t, *J* = 7.7 Hz, 2H), 1.65–1.58 (m, 2H), 1.37–1.25 (m, 6H), 0.89–0.86 (m, 3H); ^{13}C NMR (76 MHz, $CDCl_3$): δ = 151.9, 149.8, 124.0, 35.4, 31.7, 30.4, 29.0, 22.7, 14.2; HRMS (ESI+): *m/z* calcd for $C_{11}H_{17}N$ 164.1434 $[M+H]^+$, found 164.1431.

6c: R_f 0.38 (cyclohexane/EtOAc: 5/2); ^{1}H NMR (400 MHz, $CDCl_3$): δ = 8.33 (s, 1H), 8.31 (d, *J* = 5.0 Hz, 1H), 7.03 (d, *J* = 5.0 Hz, 1H), 2.63–2.56 (m, 4H), 1.62-1.52 (m, 4H), 1.42–1.26, (m, 12H), 0.91–0.88 (m, 6H); ^{13}C NMR (76 MHz, $CDCl_3$): δ = 150.5, 149.4, 147.3, 136.2, 123.9, 32.0, 31.8, 31.1, 30.3, 30.2, 29.4, 22.7, 22.7, 14.2; HRMS (ESI+): *m/z* calcd for $C_{17}H_{29}N$ 248.2373 $[M+H]^+$, found 248.2368.

2.3 Synthesis of 3,4-dioctadecanylpyridine (6e)

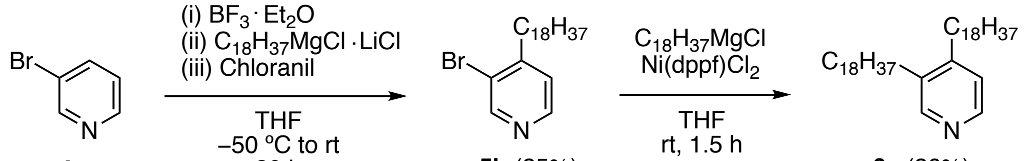

2.3.1 3-Bromo-4-octadecanylpyridine (5b)

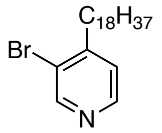

5b

The synthesis of **5a** was performed in analogy to a procedure by Knochel and coworkers.[6] To a stirred solution of 3-bromopyridine (**4**, 0.76 mL, 1.2 g, 7.8 mmol, 1.0 eq.) in dry THF (39 mL) was added BF_3 • OEt_2 (1.1 mL, 1.2 g, 8.6 mmol, 1.1 eq.) at 0 °C. The solution was stirred at 0 °C for 20−30 min and cooled to −30 to −50 °C. LiCl • $ClMgC_{18}H_{37}$ (1.1 M suspension in THF, 14 mL, 16 mmol, 2.0 eq.) was added over a period of 10 to 20 min. The orange suspension was stirred at ambient temperature for 3 h. Chloranil (3.8 g, 16 mmol, 2.0 eq.) was suspended in dry THF (15 mL) and added to the reaction mixture at −30 °C. The resulting green suspension was allowed to warm to room temperature and stirred overnight. The yellow suspension was quenched with NH_3 (13 mL, 25% w/w in H_2O). Brine (80 mL) was added, and the resulting dark suspension was extracted with Et_2O (70 mL) and ethyl acetate (3 × 100 mL). The combined organic extracts were concentrated *in vacuo*, dissolved in CH_2Cl_2 and filtered through a column of silica gel. The filtrate was concentrated *in vacuo*, diluted in CH_2Cl_2 (50 mL), washed with brine (50 mL) and dried over Na_2SO_4. Two consecutive chromatography steps (silica gel, cyclohexane/EtOAc: 20/1 and silica gel, CH_2Cl_2) yielded **5b** (820 mg, 2.0 mmol, 26%) as a white solid. R_f 0.70 (cyclohexane/EtOAc: 5/2); Mp 26 °C; ^{1}H NMR (500 MHz, $CDCl_3$): δ = 8.64 (s, 1H), 8.39 (d, *J* = 5.0 Hz, 1H), 7.14 (d, *J* = 5.0 Hz, 1H), 2.72–2.69 (m, 2H), 1.65–1.58 (m, 2H), 1.39–1.25 (m, 30H), 0.88 (t, *J* = 6.8 Hz, 3H); ^{13}C NMR (126 MHz, $CDCl_3$): δ = 152.0, 151.0, 148.3, 125.2, 123.3, 35.5, 32.1, 29.9, 29.8, 29.8, 29.8, 29.7, 29.5, 29.5, 29.4, 29.0, 22.9, 14.3; HRMS (ESI+): *m/z* calcd for $C_{23}H_{40}BrN$ 432.2236 $[M+Na]^+$, found 432.2235.

2.3.2 3,4-Dioctadecanylpyridine (6e)

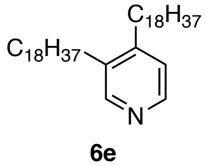

6e

The synthesis was performed following a modified procedure by Kumada and coworkers.[7] To a stirred solution of 3-bromo-4-octadecanylpyridine (**5b**, 620 mg, 1.5 mmol, 1.0 eq.) and Ni(dppp)Cl_2 (44 mg, 106 µmol, 0.07 eq.) in dry THF (6 mL) was added $C_{18}H_{37}MgCl$ (0.92 M in

THF, 2.2 mL, 2.0 mmol, 1.3 eq.) at room temperature. After stirring at room temperature for 15 min, another portion of $C_{18}H_{37}MgCl$ (0.92 M suspension in THF, 0.4 mL, 0.4 mmol, 0.25 eq.) was added to the dark suspension in two equal portions within 30 min. The reaction mixture was stirred for an additional 30 min and quenched with HCl (4 mL, 10% w/w in H_2O), after TLC indicated complete consumption of the starting material **5b**. A saturated aqueous solution of $NaHCO_3$ (8 mL) was slowly added, and the mixture was extracted with Et_2O (25 mL) and EtOAc (3 × 20 mL). The combined organic extracts were washed with brine and dried over Na_2SO_4. Purification by column chromatography (silica gel, cyclohexane/EtOAc: 20/1) yielded **6e** (230 mg, 400 µmol, 26%) as a white solid. R_f 0.55 (cyclohexane/EtOAc: 4/1); Mp 31 °C; ^{1}H NMR (400 MHz, $CDCl_3$): δ = 8.34 (s, 1H), 8.32 (d, *J* = 5.1 Hz, 1H), 7.09 (d, *J* = 5.1 Hz, 1H), 2.63–2.58 (m, 4H), 1.62–1.53 (m, 4H), 1.40–1.25 (m, 60H), 0.88 (t, *J* = 6.8 Hz, 6H); ^{13}C NMR (101 MHz, $CDCl_3$): δ = 150.5, 149.5, 147.3, 136.2, 123.9, 32.1, 32.0, 31.2, 30.4, 30.2, 29.9, 29.8, 29.8, 29.7, 29.7, 29.6, 29.5, 22.8, 14.3; HRMS (ESI+): *m/z* calcd for $C_{41}H_{77}N$ 584.6129 $[M+H]^+$, found 584.6134.

2.4 Synthesis of 4-dodecylpyridine (6d)

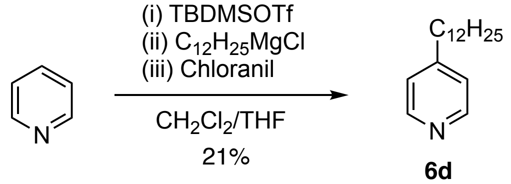

6d

The synthesis was performed following a modified procedure by Akiba *et al.*.[8] *tert*-Butyldimethylsilyl trifluoromethanesulfonate (12.4 g, 10.8 mL, 47.1 mmol, 1.0 eq.) was added dropwise to a stirred solution of dry pyridine (3.72 g, 3.80 mL, 47.1 mmol, 1.0 eq.) in dry CH_2Cl_2 (45 mL) at room temperature. After stirring at rt for 1 h, the solvent was removed *in vacuo*, and the residue was suspended in dry THF (45 mL). $C_{12}H_{25}MgBr$ (1.0 M suspension in THF, 56 mL, 56 mmol, 1.2 eq.) was added to the white suspension, and the resulting yellow solution was stirred at room temperature for 5 h. Chloranil (12.7 g, 51.8 mmol, 1.1 eq.) was added, and the resulting suspension was stirred overnight. The reaction mixture was quenched with sat. aqueous $NaHCO_3$ (130 mL) and H_2O (100 mL) and extracted with Et_2O (200 mL) and EtOAc (2 × 100 mL). The combined organic layers were washed with brine (200 mL), concentrated *in vacuo* and dissolved in CH_2Cl_2 (80 mL). The organic layer was washed again with a saturated aqueous solution of $NaHCO_3$ (50 mL), brine (100 mL) and dried over Na_2SO_4. Column chromatography (silica gel, cyclohexane/EtOAc) yielded 4-dodecylpyridine **6d** (2.40 g, 9.71 mmol, 21%) as a yellowish oil. R_f 0.28 (cyclohexane/EtOAc: 5/2); ^{1}H NMR (400 MHz, $CDCl_3$): δ = 8.48 (dd, *J* = 4.4 Hz, 1.7 Hz, 2H), 7.10 (dd, *J* = 4.4 Hz, 1.7 Hz, 2H), 2.60 (t, *J* = 7.8 Hz, 2H), 1.56–1.69 (m, 2H), 1.19–1.39 (m, 18H), 0.88 (t, *J* = 6.8 Hz, 3H); ^{13}C NMR (101 MHz, $CDCl_3$): δ = 151.9, 149.7, 124.0, 35.4, 30.4, 29.7, 29.7, 29.6, 29.5, 29.4, 29.3, 27.0, 22.8, 14.2; HRMS (ESI+): *m/z* calcd for $C_{17}H_{30}N$ 248.2373 $[M]^+$, found 248.2371.

2.5 General procedure for synthesis of 2-bromopyridines 3

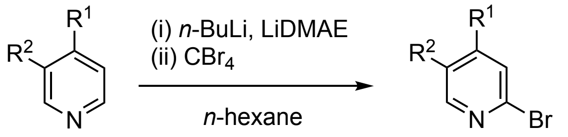

6b–e

3b: $R^1 = C_6H_{13}$, R^2 = H (47%)
3c: $R^1 = R^2 = C_6H_{13}$ (41%)
3d: $R^1 = C_{12}H_{25}$, R^2 = H (46%)
3e: $R^1 = R^2 = C_{18}H_{37}$ (36%)

The synthesis of **3** was performed following a modified procedure by Gros *et al.*.[9] To a stirred solution of 2-(dimethylamino)ethanol (2.0 eq.) in dry *n*-hexane (300 µmol/mL) was added *n*-BuLi (1.6 M solution in *n*-hexane, 4.0 eq.) dropwise at 0 °C. The colorless solution was stirred for 1–2 h at 0 °C, followed by addition of a solution of the respective alkylpyridine **6** (1.0 eq.) in dry *n*-hexane (700 µmol/mL). The resulting orange reaction mixture was stirred at 0 °C for 1–3 h, cooled to −90 °C, and treated with a solution of CBr_4 (2.5 eq.) in dry THF (1.0 mmol/mL). The resulting yellow suspension was stirred at −78 °C for 2 h and was then allowed to warm to room temperature and stirred for an additional 15 min. The resulting black suspension was cooled to 0 °C and quenched with H_2O. Brine and Et_2O were added, the organic layer was separated, and the aqueous layer was extracted with EtOAc. The combined organic extracts were dried over Na_2SO_4. Column chromatography yielded 2-bromopyridines **3** (36–51%) as brown solids or oils. Yields were not optimized.

2.5.1 2-Bromo-4-hexylpyridine (3b)

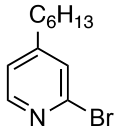

3b

According to the general procedure, 2-(dimethylamino)ethanol (390 µL, 350 mg, 3.9 mmol, 2.0 eq.) in *n*-hexane (12.6 mL) was reacted with *n*-BuLi (1.6 M solution in *n*-hexane, 4.9 mL, 7.8 mmol, 4.0 eq.) and a solution of 4-hexylpyridine (**6b**, 316 mg, 1.94 mmol, 1.0 eq.) in *n*-hexane (2.5 mL). The reaction was treated with a solution of CBr_4 (1.61 g, 4.85 mmol, 2.5 eq.) in THF (5.0 mL). The resulting black suspension was quenched with H_2O (20 mL). Brine (20 mL) and Et_2O (25 mL) were added, separated and the aqueous layer was extracted with EtOAc (3 × 30 mL). Purification by column chromatography (silica gel, cyclohexane/CH_2Cl_2: 6/4) yielded **3b** (221 mg, 913 µmol, 47%) as a light brown liquid. R_f 0.20 (cyclohexane/CH_2Cl_2: 1/1); ^{1}H NMR (400 MHz, $CDCl_3$): δ = 8.23 (d, *J* = 5.1 Hz, 1H), 7.31 (dd, *J* = 1.3, 0.6 Hz, 1H), 7.06, (dd, *J* = 5.1, 1.5 Hz, 1H), 2.57 (t, *J* = 7.8 Hz, 2H), 1.64–1.57 (m, 2H), 1.36–1.26 (m, 6H), 0.90–0.87 (m, 3H); ^{13}C NMR (76 MHz, $CDCl_3$): δ = 155.2, 150.0, 142.5, 128.1, 123.2, 35.1, 31.7, 30.2, 28.9, 22.6, 14.2; MS (EI+): *m/z* 171.0 $[M–C_5H_{10}]^+$; 162.2 $[M-Br]^+$, 92.1 $[M–Br–C_5H_{10}]^+$; HRMS (EI+): *m/z* calcd for $C_{11}H_{16}BrN$ 241.0461 $[M]^+$, found 241.0467.

2.5.2 2-Bromo-4,5-dihexylpyridine (3c)

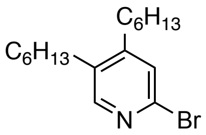

3c

According to the general procedure, 2-(dimethylamino)ethanol (80 µL, 71 mg, 0.80 mmol, 2.0 eq.) in *n*-hexane (2.6 mL) was reacted with *n*-BuLi (1.6 M in *n*-hexane, 1.0 mL, 1.6 mmol, 4.0 eq.) and a solution of 3,4-dihexylpyridine (**6c**, 99 mg, 400 µmol, 1.0 eq.) in *n*-hexane (0.60 mL). The reaction was treated with a solution of CBr_4 (332 mg, 1.00 mmol, 2.5 eq.) in THF (1.0 mL). The resulting black suspension was quenched with H_2O (4 mL). Brine (8 mL) and Et_2O (15 mL) were added, separated and the aqueous layer was extracted with EtOAc (3 × 15 mL). The combined organic extracts were dried over Na_2SO_4. Two consecutive chromatography steps (silica gel, cyclohexane/CH_2Cl_2, 7/3 and silica gel, cyclohexane/EtOAc: 70/1) yielded **3c** (52.9 mg, 162 µmol, 41%) as a light yellow liquid. R_f 0.65 (cyclohexane/EtOAc: 10/1); ^{1}H NMR (300 MHz, $CDCl_3$): δ = 8.07 (s, 1H), 7.23 (s, 1H), 2.57-2.52 (m, 4H), 1.62-1.48 (m, 4H), 1.41-1.25 (m, 12H), 0.93-0.85 (m, 6H); ^{13}C NMR (76 MHz, $CDCl_3$): δ = 153.0, 150.5, 139.7, 135.7, 127.7, 31.9, 31.7, 31.7, 30.8, 30.1, 29.6, 29.4, 29.3, 22.7, 22.7, 14.2; HRMS (ESI+): *m/z* calcd for $C_{17}H_{28}BrN$ 348.1297 $[M+Na]^+$, found 348.1303.

2.5.3 2-Bromo-4-dodecylpyridine (3d)

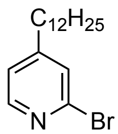

3d

According to the general procedure, 2-(dimethylamino)ethanol (925 µL, 825 mg, 9.25 mmol, 2.0 eq.) in *n*-hexane (29 mL) was reacted with *n*-BuLi (1.6 M solution in *n*-hexane, 10.7 mL, 17.6 mmol, 4.0 eq.) and a solution of 4-dodecylpyridine (**6d**, 1.09 g, 4.41 mmol, 1.0 eq.) in *n*-hexane (7.0 mL). The reaction was treated with a solution of CBr_4 (3.66 g, 11.0 mmol, 2.5 eq.) in THF (11 mL). The resulting black suspension was quenched with H_2O (50 mL). Brine (100 mL) and Et_2O (50 mL) were added, separated and the aqueous layer was extracted with EtOAc (3 × 100 mL). Purification by column chromatography (silica gel, cyclohexane/EtOAc) yielded **3d** (659 mg, 2.02 mmol, 46%) as a light brown liquid. R_f 0.60 (cyclohexane/EtOAc: 5/1); ^{1}H NMR (400 MHz, $CDCl_3$): δ = 8.23 (dd, *J* = 5.1 Hz, 0.5 Hz, 1H), 7.31 (dd, *J* = 1.5 Hz, 0.5 Hz, 1H), 7.06, (dd, *J* = 5.1, 1.5 Hz, 1H), 2.57 (t, *J* = 7.8 Hz, 2H), 1.67–1.56 (m, 2H), 1.33–1.23 (m, 6H), 0.88 (t, *J* = 6.8 Hz, 3H); ^{13}C NMR (101 MHz, $CDCl_3$): δ = 155.2, 149.8, 142.4, 128.1, 123.2, 35.0, 32.0, 30.2, 29.7, 29.7, 29.6, 29.5, 29.4, 29.2, 22.8, 14.2; HRMS (EI+): *m/z* calcd for $C_{17}H_{29}NBr$ 326.1478 $[M]^+$, found 326.1479.

2.5.4 2-Bromo-4,5-dioctadecanylpyridine (3e)

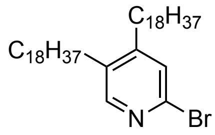

3e

According to the general procedure, 2-(dimethylamino)ethanol (120 µL, 107 mg, 1.20 mmol, 2.0 eq.) in *n*-hexane (3.8 mL) was reacted with *n*-BuLi (1.6 M solution in *n*-hexane, 1.5 mL, 2.4 mmol, 4.0 eq.) and a solution of 3,4-dioctadecanylpyridine (**6e**, 342 mg, 586 µmol, 1.0 eq.) in *n*-hexane (0.90 mL). The reaction was treated with a solution of CBr_4 (486 mg, 1.47 mmol, 2.5 eq.) in THF (1.5 mL). The resulting black suspension was quenched with H_2O (6 mL). Brine (10 mL) and Et_2O (15 mL) were added, separated and the aqueous layer was extracted with EtOAc (3 × 15 mL). Two consecutive chromatography steps (silica gel, cyclohexane/EtOAc: 200/1 and silica gel, cyclohexane/CH_2Cl_2, 10/1) yielded **3e** (140 mg, 211 µmol, 36%) as a light yellow solid. R_f 0.25 (cyclohexane/EtOAc: 100/1); Mp 34 °C; ^{1}H NMR (400 MHz, $CDCl_3$): δ = 8.08 (s, 1H), 7.24 (s, 1H), 2.55 (t, *J* = 7.8 Hz, 2H), 2.55 (t, *J* = 7.8 Hz, 2H), 1.61–1.50 (m, 4H), 1.40–1.26 (m, 60H), 0.88 (t, *J* = 6.8 Hz, 6H); ^{13}C NMR (101 MHz, $CDCl_3$): δ = 153.0, 150.5, 139.7, 135.7, 127.7, 32.1, 31.9, 30.9, 30.1, 29.9, 29.8, 29.8, 29.8, 29.7, 29.7, 29.7, 29.6, 29.6, 29.6, 29.5, 29.5, 22.9, 14.3; HRMS (ESI+): *m/z* calcd for $C_{41}H_{76}BrN$ 662.5234 $[M+H]^+$, found 662.5245.

2.6 General Procedure for Synthesis of Tris[µ-(pyridinato-N^1,C^2)]tri-gold(I) complexes 1

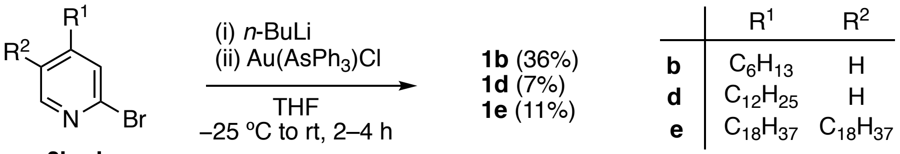

3b, d, e

The synthesis was performed following a modified procedure by Vaughan *et al.*.[10] To a stirred solution of 2-bromopyridine **3** in dry THF (40–60 µmol/mL) was added *n*-BuLi (0.3–1.6 M solution in *n*-hexane, 1.0 eq.) dropwise at −40 to −25 °C, and the resulting orange or red reaction mixture was stirred for 1 h. Chloro(triphenylarsine)gold(I) (1.0 eq.) was suspended in THF (50–100 µmol/mL) at −40 °C and added to the reaction mixture. The resulting suspension was allowed to warm to room temperature over a period of 1–3 h, and the mixture was concentrated *in vacuo*. The residue was filtered through a plug of alumina and either purified by recrystallization from toluene and benzene or column chromatography (alumina, cyclohexane/toluene or cyclohexane/CH_2Cl_2) to yield pyridinates **1** in yields of 6–37%. Yields are not optimized.

2.6.1 Tris[μ-(4-hexylpyridinato-N[1],C[2])]tri-gold(I) (1b)

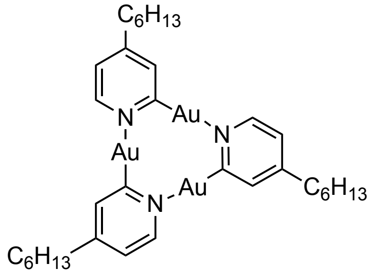

1b

According to the general procedure, 2-bromo-4-hexanylpyridine (**3a**, 78.0 mg, 322 µmol, 1.0 eq.) in dry THF (7 mL) was reacted with *n*-BuLi (1.7 M solution in *n*-hexane, 0.19 mL, 320 µmol, 1.0 eq.) and chloro(triphenylarsine)gold(I) (173 mg, 322 µmol, 1.0 eq.) in dry THF (3 mL). Two consecutive column chromatography steps (alumina, cyclohexane/CH_2Cl_2 and alumina, cyclohexane/benzene) yielded **1b** (42.1 mg, 39.1 µmol, 36%) as a colorless wax. R_f 0.17 (alumina, cyclohexane/CH_2Cl_2: 5/1); ^{1}H NMR (400 MHz, C_6D_6): δ = 8.13 (d, *J* = 5.9 Hz, 3H), 7.67 (d, *J* = 1.8 Hz, 3H), 6.26 (dd, *J* = 5.9 Hz, 1.8 Hz, 3H), 2.15 (t, *J* = 7.7 Hz, 6H), 1.38-1.27 (m, 6H), 1.25-1.10 (m, 18H), 0.87 (t, *J* = 7.2 Hz, 9H); ^{13}C NMR (101 MHz, C_6D_6): δ = 184.6, 151.3, 150.5, 138.7, 121.2, 35.4, 32.0, 30.1, 29.3, 22.9, 14.3; MS (ESI+): *m/z* calcd for $C_{33}H_{48}Au_3N_3$ 1100.3 $[M+Na]^+$, found 1100.4.

2.6.2 Tris[µ-(4,5-dihexanylpyridinato-N[1],C[2])]tri-gold(I) (1c)

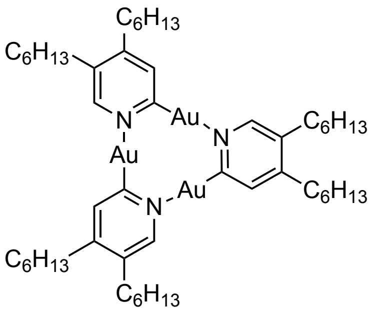

1c

According to the general procedure, 2-bromo-4,5-dihexanylpyridine (**3b**, 40 mg, 120 µmol, 1.0 eq.) in dry THF (4 mL) was reacted with *n*-BuLi (0.28 M solution in *n*-hexane, 0.44 mL, 120 µmol, 1.0 eq.) and chloro(triphenylarsine)gold(I) (, 66 mg, 120 µmol, 1.0 eq.) in dry THF (3 mL). The residue was purified by several column chromatography steps (alumina, cyclohexane/toluene) to yield **1c** (3.0 mg, 6.8 µmol, 6%) as a colorless wax. Significant amounts of $AsPh_3$ and traces of other unknown impurities could not be completely removed. R_f 0.35 (alumina, toluene); ^{1}H NMR

(500 MHz, C_6D_6): δ = 8.27 (s, 3H), 7.79 (s, 3H), 2.34 (t, *J* = 7.8 Hz, 6H), 2.28 (t, *J* = 7.8 Hz, 6H), 1.51–1.43 (m, 6H), 1.37–1.14 (m, 42H), 0.85–0.93 (m, 18H); ^{13}C NMR (126 MHz, C_6D_6): δ = 181.1, 151.6, 148.9, 138.5, 133.4, 32.0, 32.0, 32.0, 30.8, 30.0, 29.9, 29.7, 23.0, 22.9, 14.4, 14.3; HRMS (ESI+): *m/z* calcd for $C_{51}H_{84}Au_3N_3$ 1352.5554 $[M+Na]^+$, found 1352.5535.

2.6.3 Tris[*μ*-(4-dodecylpyridinato-N[1],C[2])]tri-gold(I) (1d)

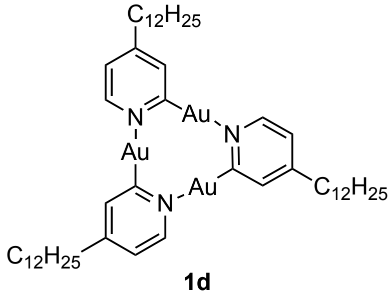

1d

According to the general procedure, 2-bromo-4-hexanylpyridine (**3c**, 178 mg, 545 µmol, 1.0 eq.) in dry THF (12 mL) was reacted with *n*-BuLi (1.6 M solution in *n*-hexane, 0.34 mL, 550 µmol, 1.0 eq.) and solid chloro(triphenylarsine)gold(I) (294 mg, 545 µmol, 1.0 eq.). Two consecutive column chromatography steps (alumina, cyclohexane/CH_2Cl_2 and Al_2O_3, cyclohexane/benzene) and several recrystallization steps from toluene yielded **1d** (17.1 mg, 12.8 µmol, 7%) as white solid. R_f 0.2 (alumina, cyclohexane/CH_2Cl_2: 5/1); ^{1}H NMR (400 MHz, C_6D_6): δ = 8.12 (d, *J* = 5.8 Hz, 3H), 7.68 (d, *J* = 1.7 Hz, 3H), 6.27 (dd, *J* = 5.9 Hz, 1.8 Hz, 3H), 2.19 (t, *J* = 7.8 Hz, 6H), 1.44–1.15 (m, 60H), 0.92 (t, *J* = 7.2 Hz, 9H); ^{13}C NMR (101 MHz, C_6D_6): δ = 184.4, 151.3, 150.6, 138.7, 121.3, 35.4, 32.4, 30.2, 30.2, 29.9, 29.9, 29.8, 29.6, 22.9, 14.3; MS (MALDI–TOF): *m/z* calcd for $C_{51}H_{84}Au_3N_3$ 1329.6 $[M]^+$, found 1329.6.

2.6.4 Tris[µ-(4,5-dioctadecanylpyridinato-N[1],C[2])]tri-gold(I) (1e)

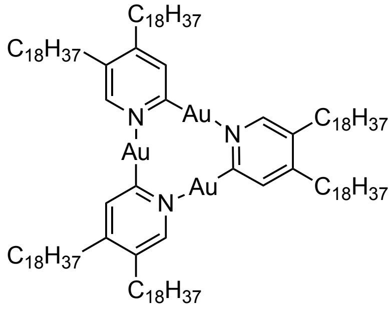

1e

According to the general procedure, 2-bromo-4,5-dioctadecanylpyridine (**3e**, 135 mg, 204 µmol, 1.0 eq.) in dry THF (5 mL) was reacted with *n*-BuLi (0.30 M solution in *n*-hexane, 0.68 mL, 204 µmol, 1.0 eq.) and chloro(triphenylarsine)gold(I) (110 mg, 204 µmol, 1.0 eq.) in dry THF (4 mL). The residue was recrystallized twice from toluene (2.5 mL), decanted and washed with cold toluene (4 × 0.5 mL). The remaining solid was dissolved in hot toluene and filtered through a plug of alumina at 50 °C. The crude product was recrystallized from benzene, filtered through a plug of alumina and dried *in vacuo* to yield **1e** (24.7 mg, 10.6 µmol, 16%) as a white solid. Mp 70 °C (decomposition at 128 °C); ^{1}H NMR (500 MHz, C_6D_6, 313 K): δ = 8.31 (s, 3H), 7.80 (s, 3H), 2.43 (t, *J* = 7.9 Hz, 6H), 2.37 (t, *J* = 8.0 Hz, 6H), 1.59–1.52 (m, 6H), 1.46–1.31 (m, 186H), 0.93 (t, *J* = 6.9 Hz, 18H); ^{13}C NMR (126 MHz, C_6D_6, 313 K): δ = 181.2,

151.7, 148.8, 138.6, 133.4, 32.4, 32.1, 31.0, 30.3, 30.3, 30.3, 30.2, 30.2, 30.1, 30.1, 30.0, 30.0, 30.0, 29.9, 29.9, 29.8, 23.1, 14.4; MS (MALDI–TOF): *m*/*z* calcd for $C_{69}H_{120}Au_3N_3$ 2338.69 $[M]^+$, found 2338.76.

3 NMR titration experiments

Binding constants were determined through NMR titrations in C_6D_6 and $CDCl_3$ according to a procedure by Thordarson *et al.*.[11] All titration experiments were carried out at constant concentration of the donor **1**, therefore all solutions of acceptors **2** were prepared using a stock solution of the donor **1**. Experiments were carried out as follows: A stock solution of donor **1** (ca. 4 mL) was prepared, and four NMR-tubes were filled with different amounts of this stock solution (0.5 to 0.15 mL). Three titration solutions containing different concentrations of the acceptors **2** were prepared from the initial donor stock solution. Each NMR-tube was then filled to a total volume of 0.5 mL. ^{1}H-NMR spectra were measured, and four to six small portions of acceptor solution were successively added. Each new sample was analyzed by ^{1}H NMR spectroscopy. All volumes were quantified by weighing using Hamilton syringes, and the weight differences were estimated and used for calculation of the concentrations. The measured data was fitted to a 1:2 binding model, using either the website supramolecular.org or a MatLab code provided by Thordarson *et al.*.[11] All titrations between donor **1b** and acceptors **2a**–**c** were performed three times, and the data are listed in the following.

3.1 Titration of pyridinate 1b with gold pyrazolate 2a

3.1.1 Data of experiment 1

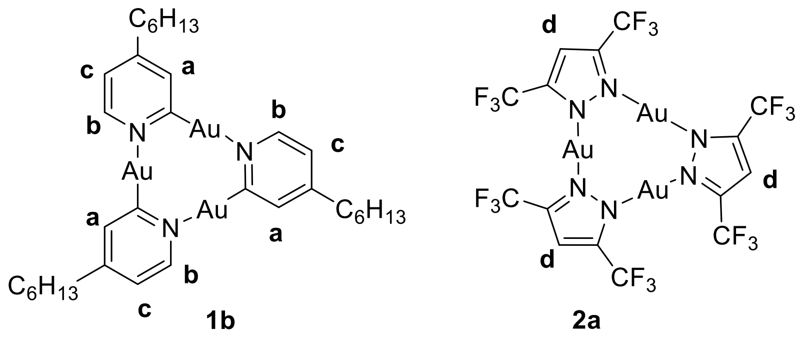

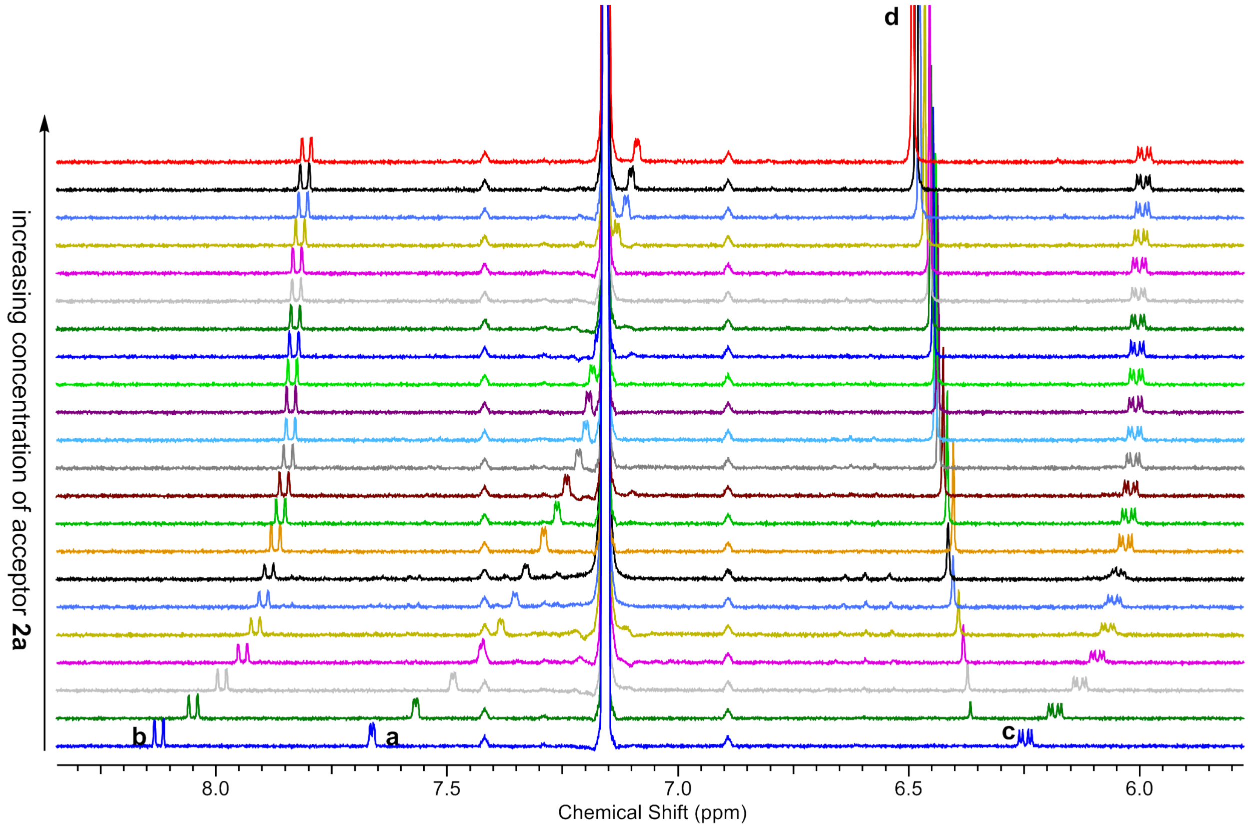

Figure S 1. ^{1}H NMR spectra for the titration of 1b with 2a (Experiment 1).

Table S 1. Measured data of the titration experiment between 1b and 2a, fitted to a non-linear 1:1 binding model (Experiment 1).

K_{11} [L mol^{-1}]		δ_H	δ_{HG}	RMS	Cov
1.50×10^{3}	proton b	8.134	7.842	1.79×10^{-2}	4.89×10^{-2}
**K_{11} error [%]**	proton a	7.663	7.192	6.52×10^{-2}	1.81×10^{-1}
66.5	proton c	6.261	6.019	1.10×10^{-2}	2.83×10^{-2}
	total			3.95×10^{-2}	8.62×10^{-2}

Table S 2. Measured data of the titration experiment between 1b and 2a, fitted to a non-linear 1:2 binding model (Experiment 1).

K_{11} [L mol^{-1}]	K_{12} [L mol^{-1}]		δ_H [ppm]	δ_{HG} [ppm]	δ_{HG2} [ppm]	RMS	Cov
1.52×10^{4}	446	proton b	8.133	7.881	7.802	2.06×10^{-3}	6.40×10^{-4}
**K_{11} error [%]**	**K_{12} error [%]**	proton a	7.662	7.342	7.032	3.15×10^{-3}	4.22×10^{-4}
6.46	2.15	proton c	6.260	6.042	5.995	1.68×10^{-3}	6.51×10^{-4}
		total				2.38×10^{-3}	3.11×10^{-4}

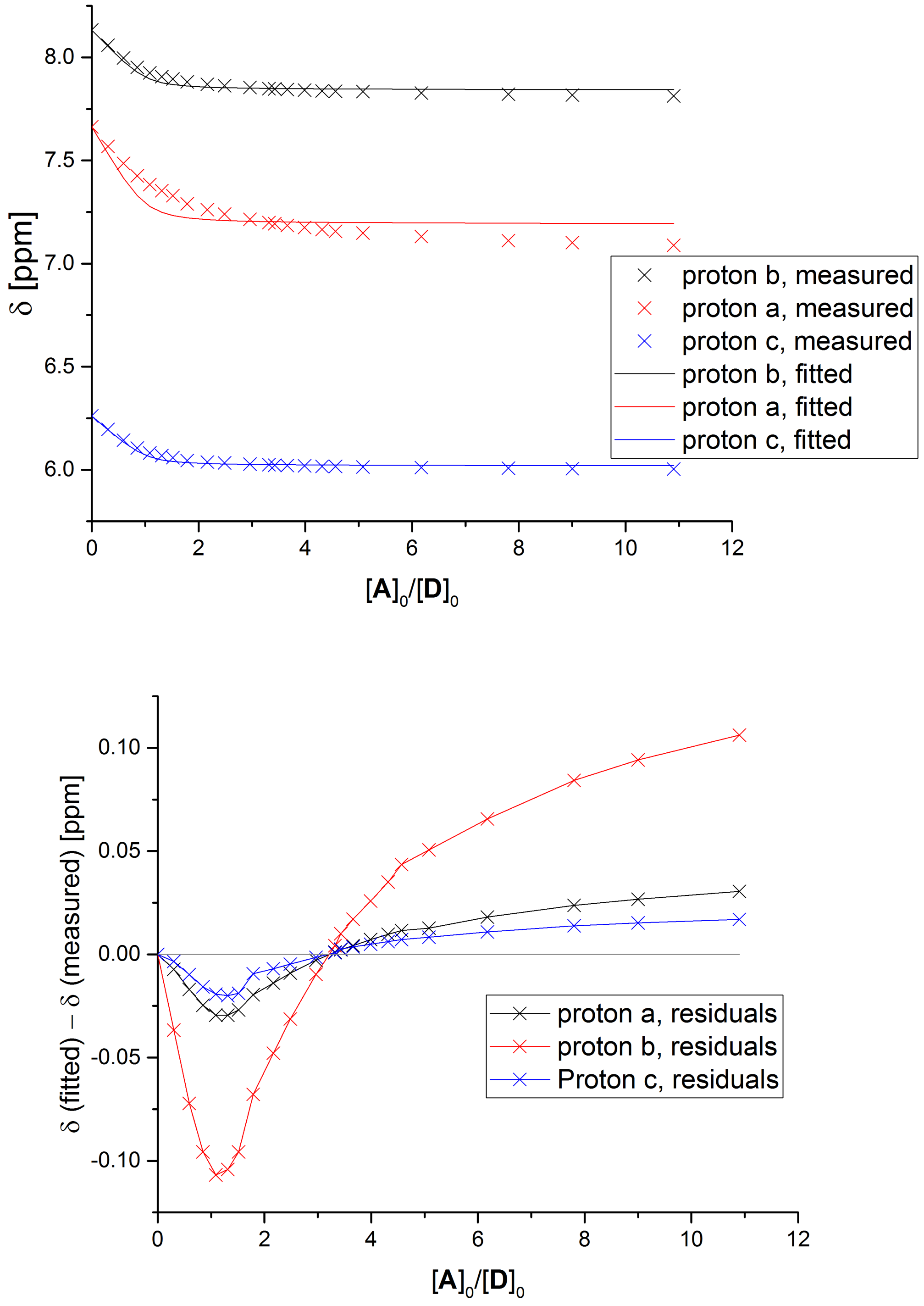

Figure S 2. Binding isotherms of the titration experiment between 1b and 2a, fitted to a 1:1 binding model, and the corresponding residuals between fitted and measured values (Experiment 1).

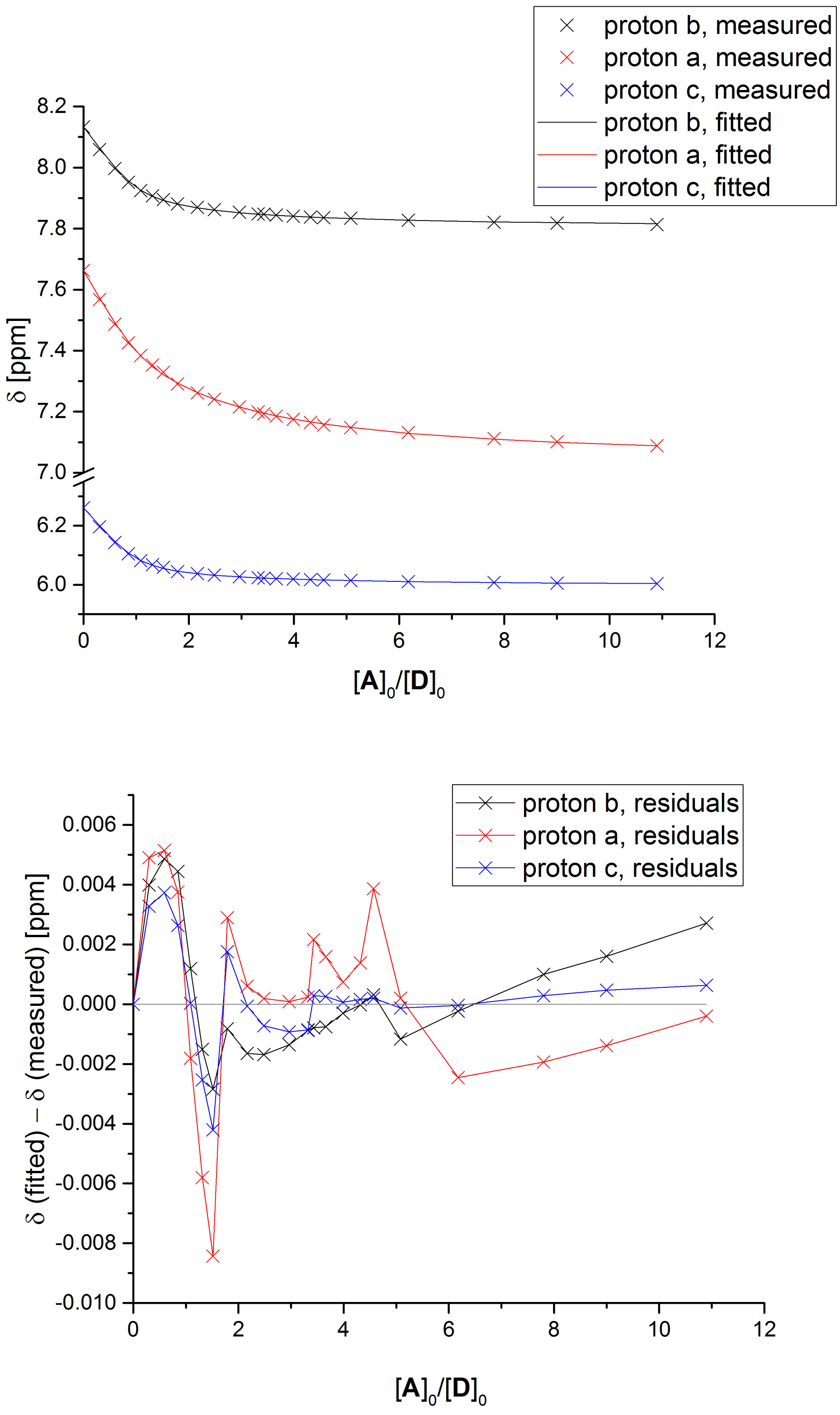

Figure S 3. Binding isotherms of the titration experiment between 1b and 2a, fitted to a 1:2 binding model, and the corresponding residuals between fitted and measured values (Experiment 1).

3.1.2 Data of experiment 2

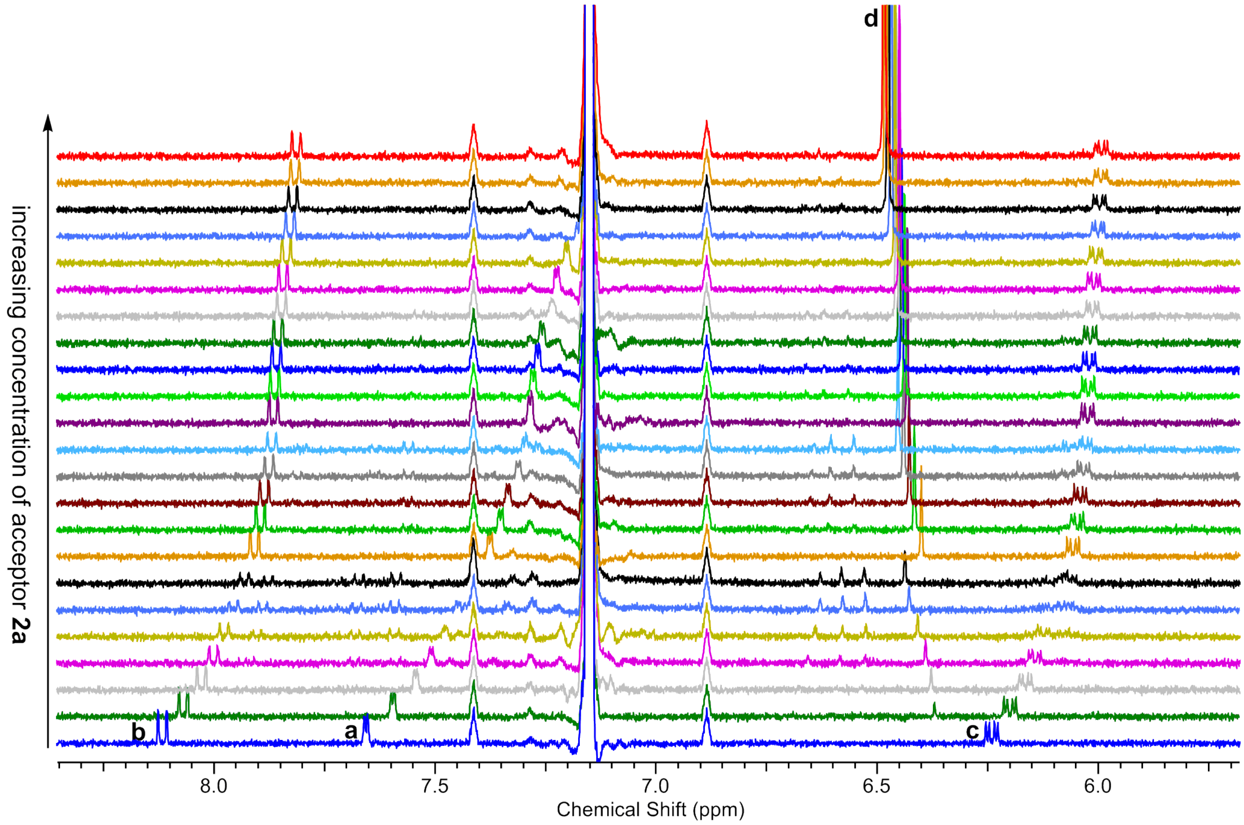

Figure S 4. ^{1}H NMR spectra for the titration of 1b with 2a (Experiment 2).

Table S 3. Measured data of the titration experiment between 1b and 2a, fitted to a non-linear 1:2 binding model (Experiment 2).

K_{11} [L mol^{-1}]	K_{12} [L mol^{-1}]		δ_H [ppm]	δ_{HG} [ppm]	δ_{HG2} [ppm]	RMS	Cov
5.09×10^{4}	574	proton b	8.127	7.909	7.790	2.70×10^{-3}	1.08×10^{-3}
**K_{11} error [%]**	**K_{12} error [%]**	proton a	7.661	7.039	7.048	4.25×10^{-3}	8.67×10^{-4}
14.4	3.29	proton c	6.254	6.051	5.992	4.92×10^{-3}	5.60×10^{-4}
		total				4.06×10^{-3}	1.17×10^{-3}

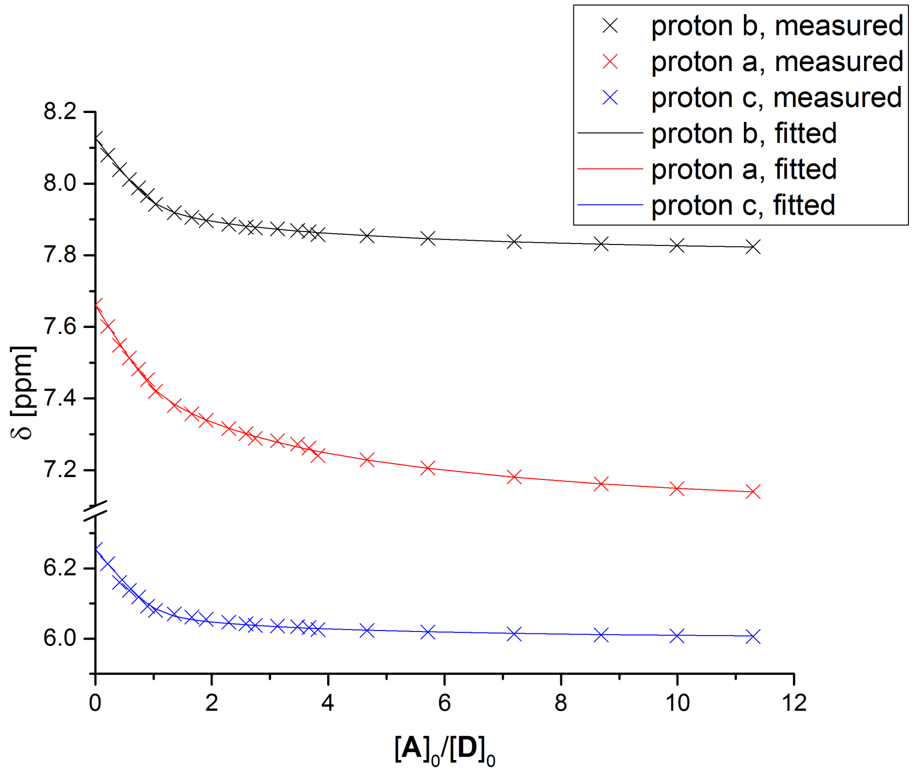

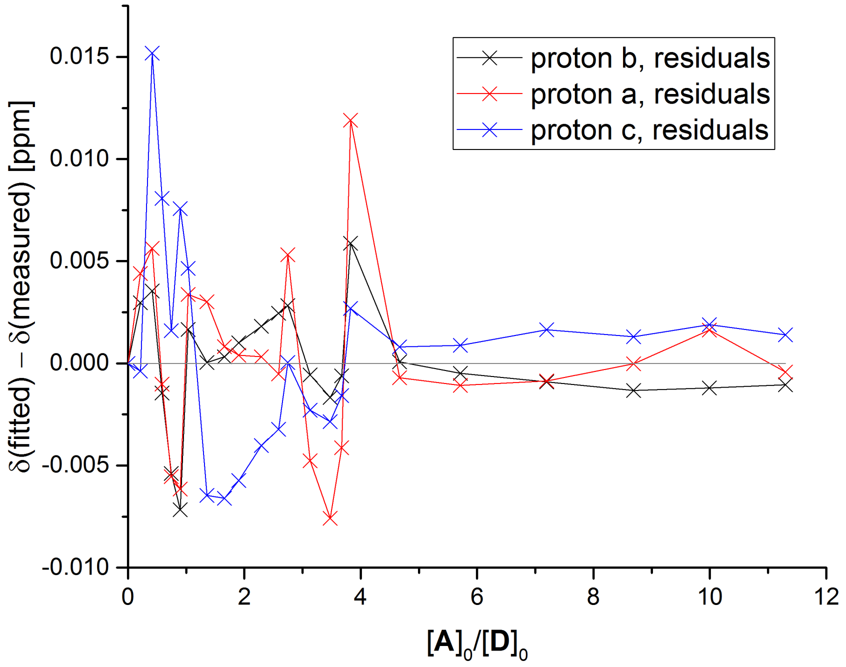

Figure S 5. Binding isotherms of the titration experiment between 1b and 2a, fitted to a 1:2 binding model and the corresponding residuals between fitted and measured values (Experiment 2).

3.1.3 Data of experiment 3

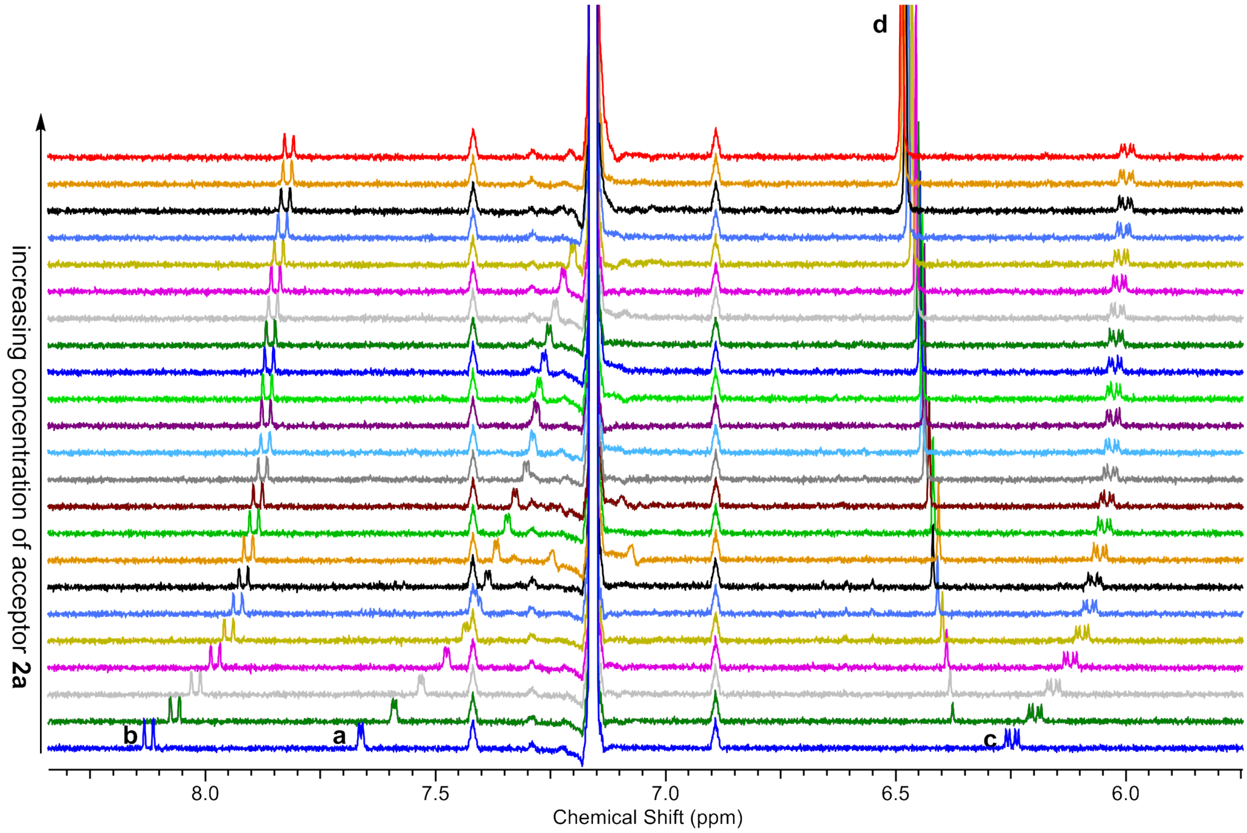

Figure S 6. ^{1}H NMR spectra for the titration of 1b with 2a (Experiment 3).

Table S 4. Measured data of the titration experiment between 1b and 2a, fitted to a non-linear 1:2 binding model (Experiment 3).

K_{11} [L mol^{-1}]	K_{12} [L mol^{-1}]		δ_H [ppm]	δ_{HG} [ppm]	δ_{HG2} [ppm]	RMS	Cov
2.41×10^4	526	proton b	8.132	7.896	7.800	1.44×10^{-3}	3.35×10^{-4}
**K_{11} error [%]**	**K_{12} error [%]**	proton a	7.666	7.365	7.045	2.39×10^{-3}	2.97×10^{-4}
4.03	1.69	proton c	6.260	6.051	5.993	1.74×10^{-3}	7.19×10^{-4}
		total				1.90×10^{-3}	2.58×10^{-4}

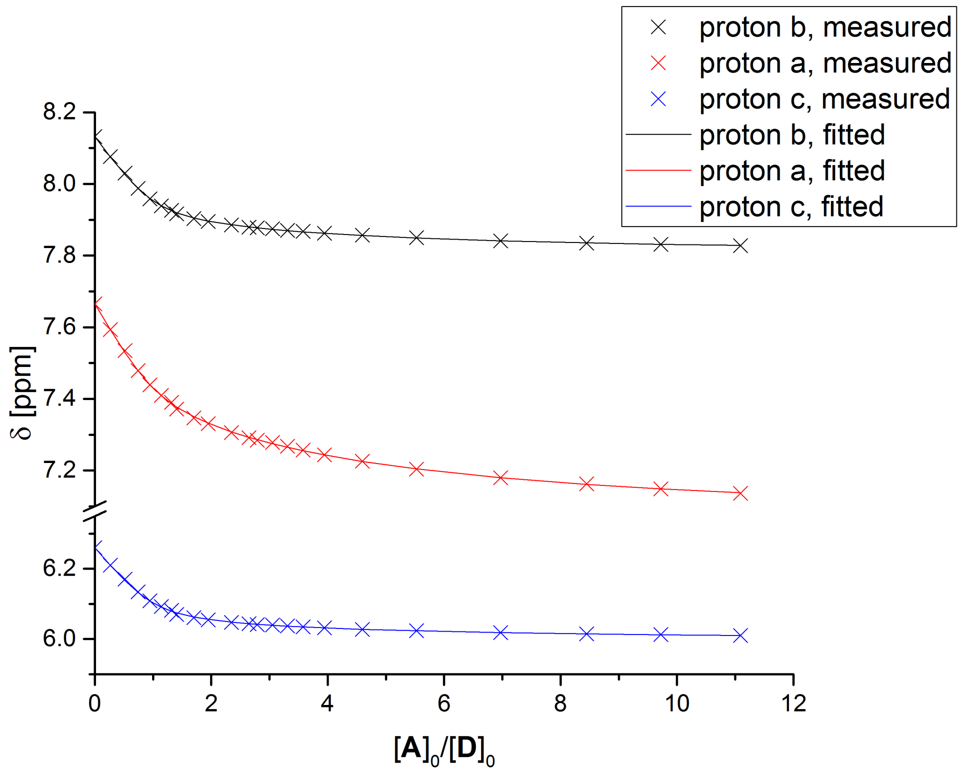

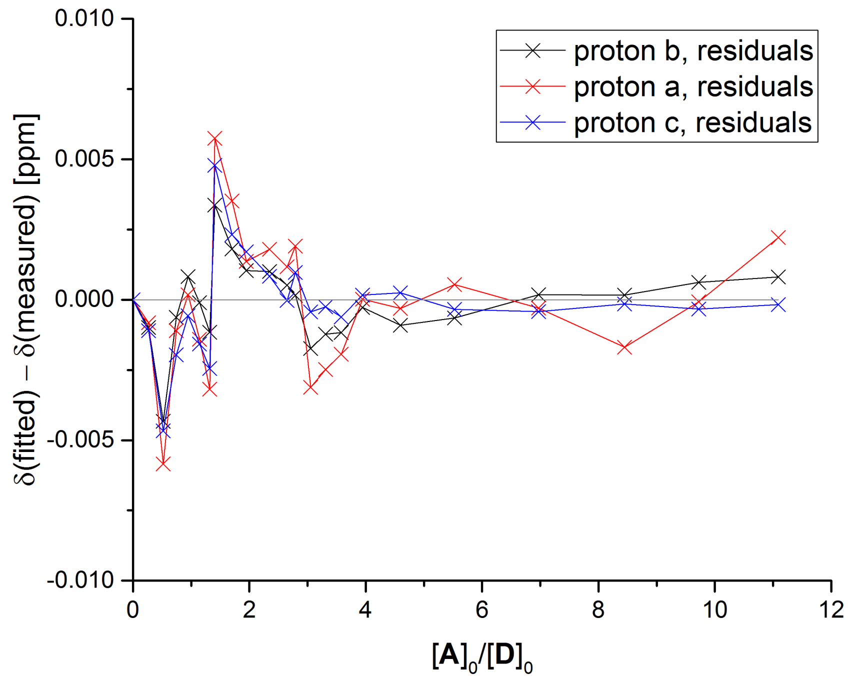

Figure S 7. Binding isotherms of the titration experiment between 1b and 2a, fitted to a 1:2 binding model, and the corresponding residuals between fitted and measured values (Experiment 3).

3.2 Titration of pyridinate 1b with copper pyrazolate 2b

3.2.1 Data of experiment 1

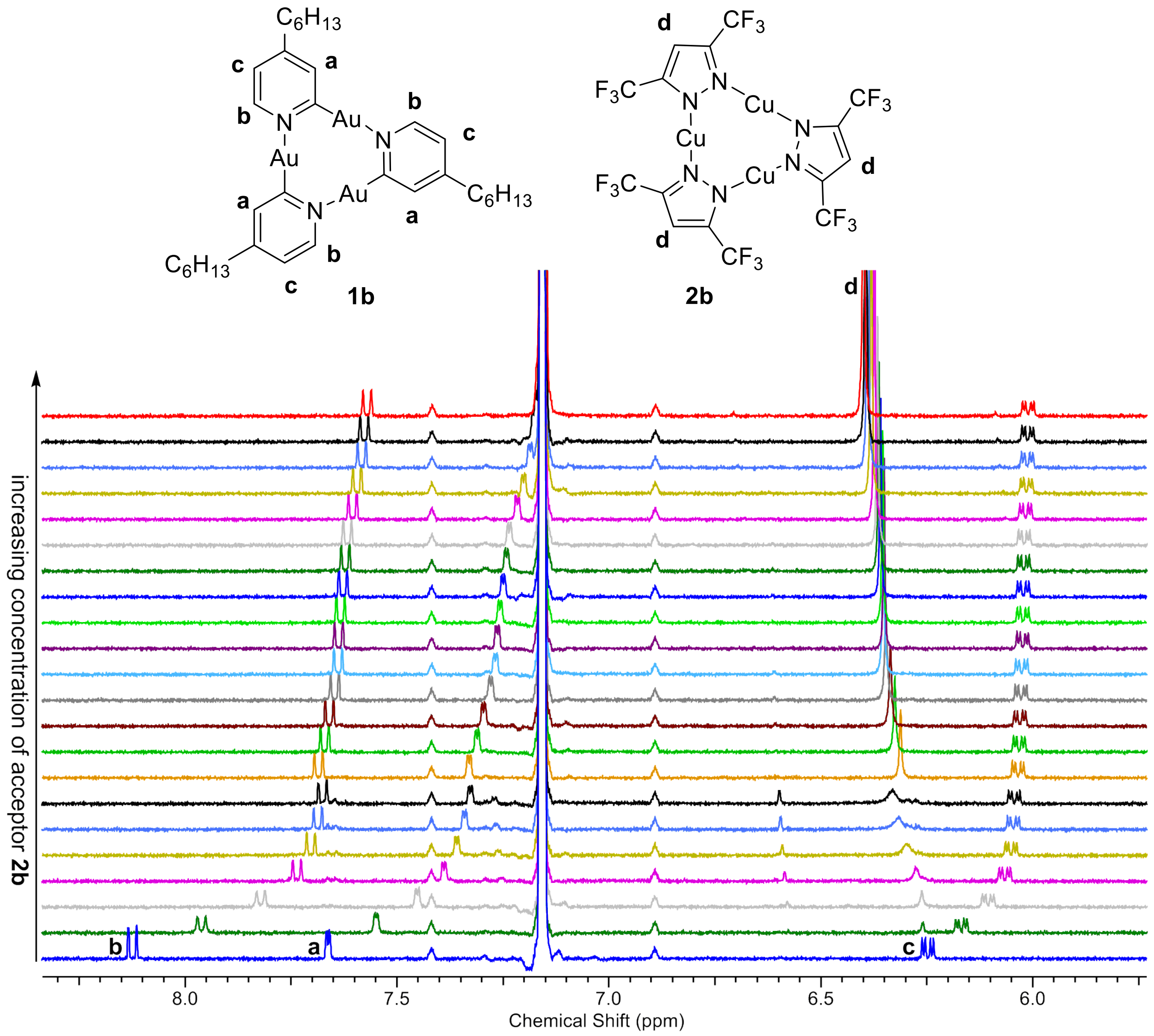

Figure S 8. ^{1}H NMR spectra for the titration of 1b with 2b (Experiment 1).

Table S 5. Measured data of the titration experiment between 1b and 2b, fitted to a non-linear 1:2 binding model (Experiment 1).

K_{11} [L mol^{-1}]	K_{12} [L mol^{-1}]		δ_H [ppm]	δ_{HG} [ppm]	δ_{HG2} [ppm]	RMS	Cov
1.00×10^5	225	proton b	8.134	7.704	7.540	5.66×10^{-3}	1.93×10^{-3}
**K_{11} error [%]**	**K_{12} error [%]**	proton a	7.663	7.358	7.099	3.63×10^{-3}	9.43×10^{-4}
28.23	3.78	proton c	6.261	6.055	6.010	2.94×10^{-3}	2.74×10^{-3}
		total				4.24×10^{-3}	8.49×10^{-4}

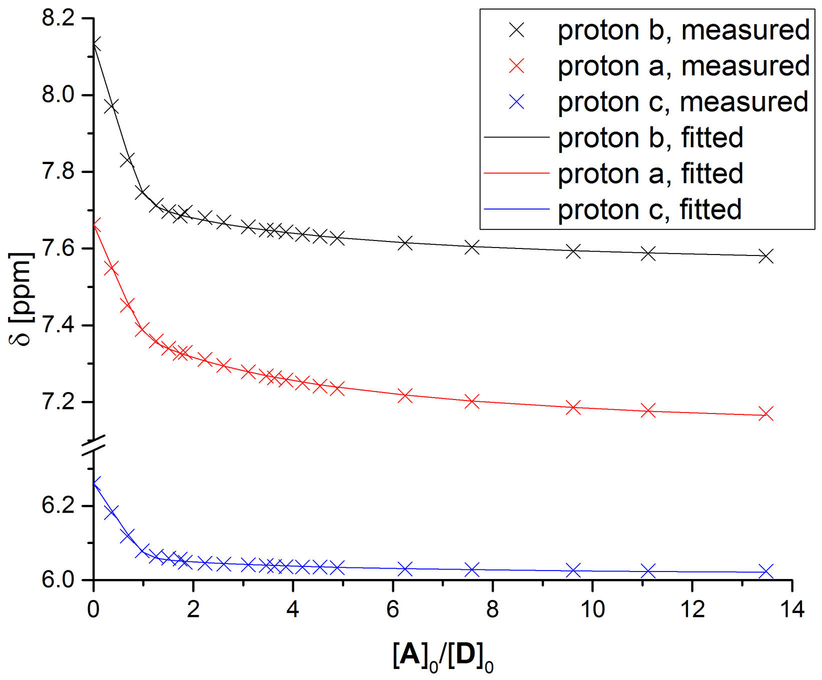

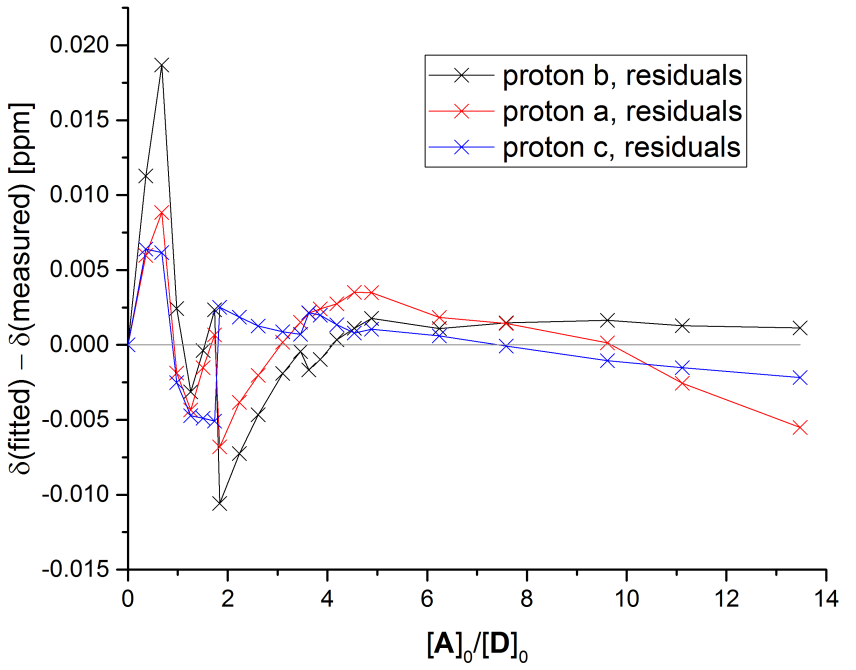

Figure S 9. Binding isotherms of the titration experiment between 1b and 2b, fitted to a 1:2 binding model, and the corresponding residuals between fitted and measured values (Experiment 1).

3.2.2 Data of experiment 2

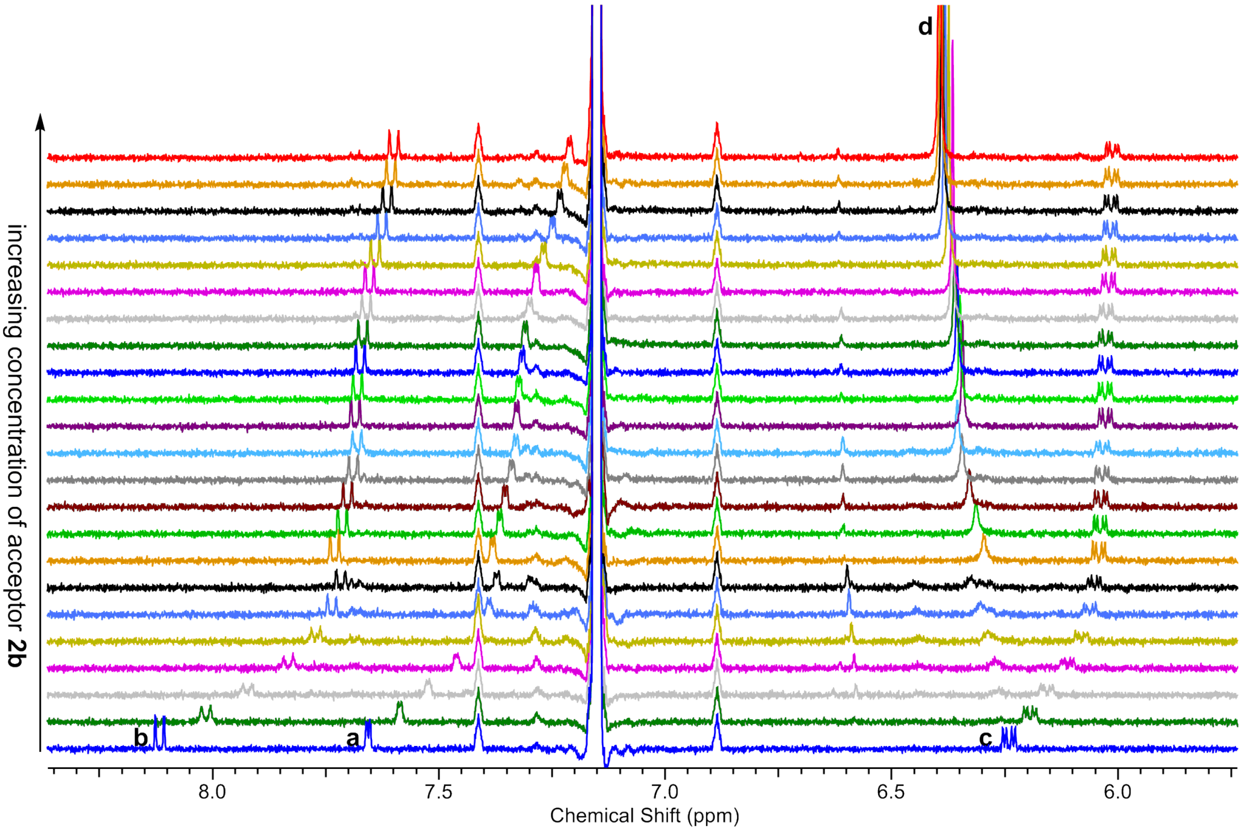

Figure S 10. ^{1}**H NMR spectra for the titration of 1b with 2b (Experiment 2).**

Table S 6. Measured data of the titration experiment between 1b and 2b, fitted to a non-linear 1:2 binding model (Experiment 2).

K_{11} [L mol^{-1}]	K_{12} [L mol^{-1}]		δ_H [ppm]	δ_{HG} [ppm]	δ_{HG2} [ppm]	RMS	Cov
1.65×10^{5}	341	proton b	8.127	7.727	7.541	3.38×10^{-3}	7.02×10^{-4}
**K_{11} error [%]**	**K_{12} error [%]**	proton a	7.661	7.383	7.119	2.49×10^{-3}	5.15×10^{-4}
17.8	3.45	proton c	6.254	6.059	5.999	4.90×10^{-3}	6.63×10^{-3}
		total				3.73×10^{-3}	7.78×10^{-4}

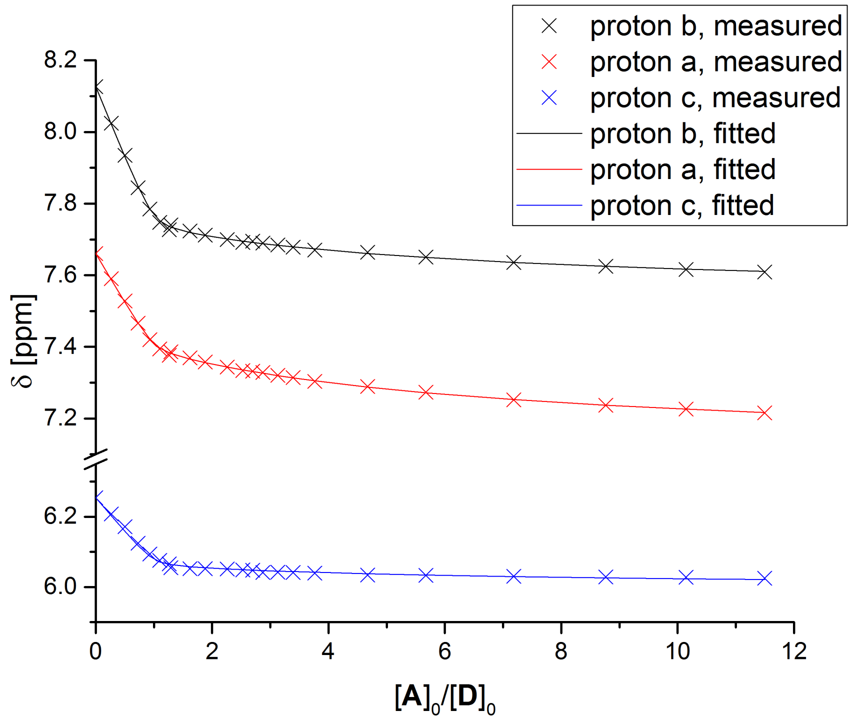

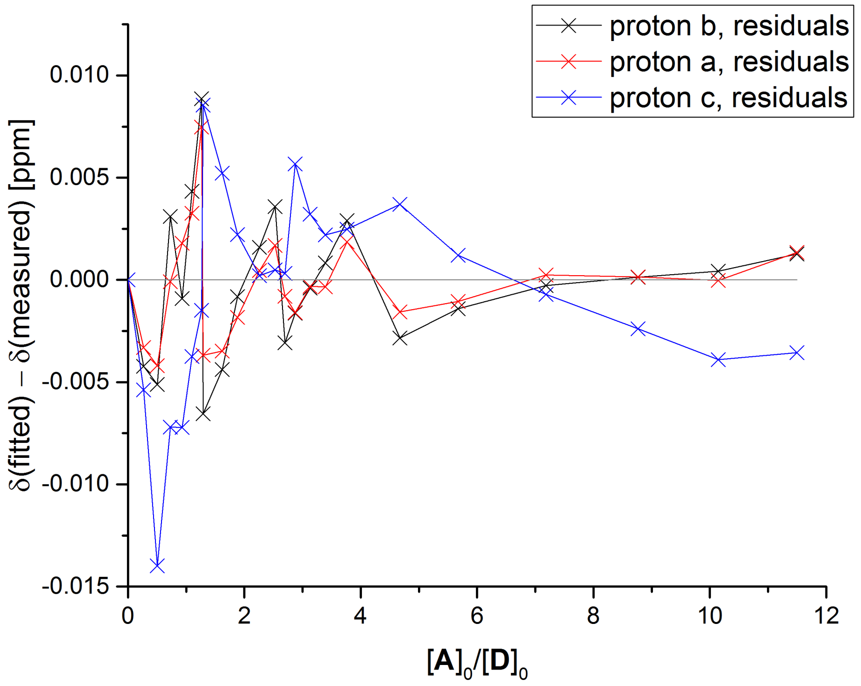

Figure S 11. Binding isotherms of the titration experiment between 1b and 2b, fitted to a 1:2 binding model, and the corresponding residuals between fitted and measured values (Experiment 2).

3.2.3 Data of experiment 3

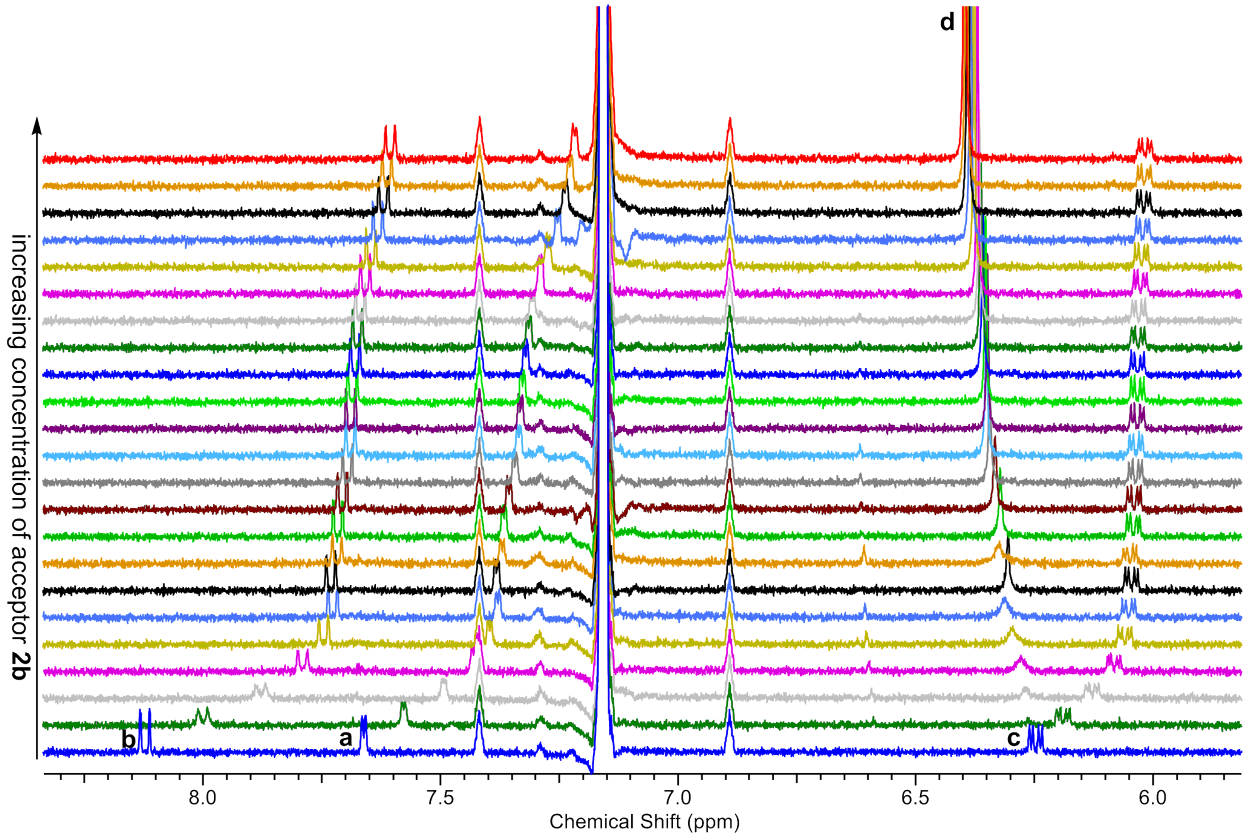

Figure S 12. ¹H NMR spectra for the titration of 1b with 2b (Experiment 3).

Table S 7. Measured data of the titration experiment between 1b and 2b, fitted to a non-linear 1:2 binding model (Experiment 3).

K_{11} [L mol^{-1}]	K_{12} [L mol^{-1}]		δ_H [ppm]	δ_{HG} [ppm]	δ_{HG2} [ppm]	RMS	Cov
2.05×10^5	214	proton b	8.132	7.728	7.506	5.98×10^{-3}	2.46×10^{-3}
**K_{11} error [%]**	**K_{12} error [%]**	proton a	7.666	7.377	7.057	4.43×10^{-3}	1.69×10^{-3}
25.8	3.76	proton c	6.260	6.055	6.003	2.22×10^{-3}	1.58×10^{-3}
		total				4.44×10^{-3}	1.19×10^{-3}

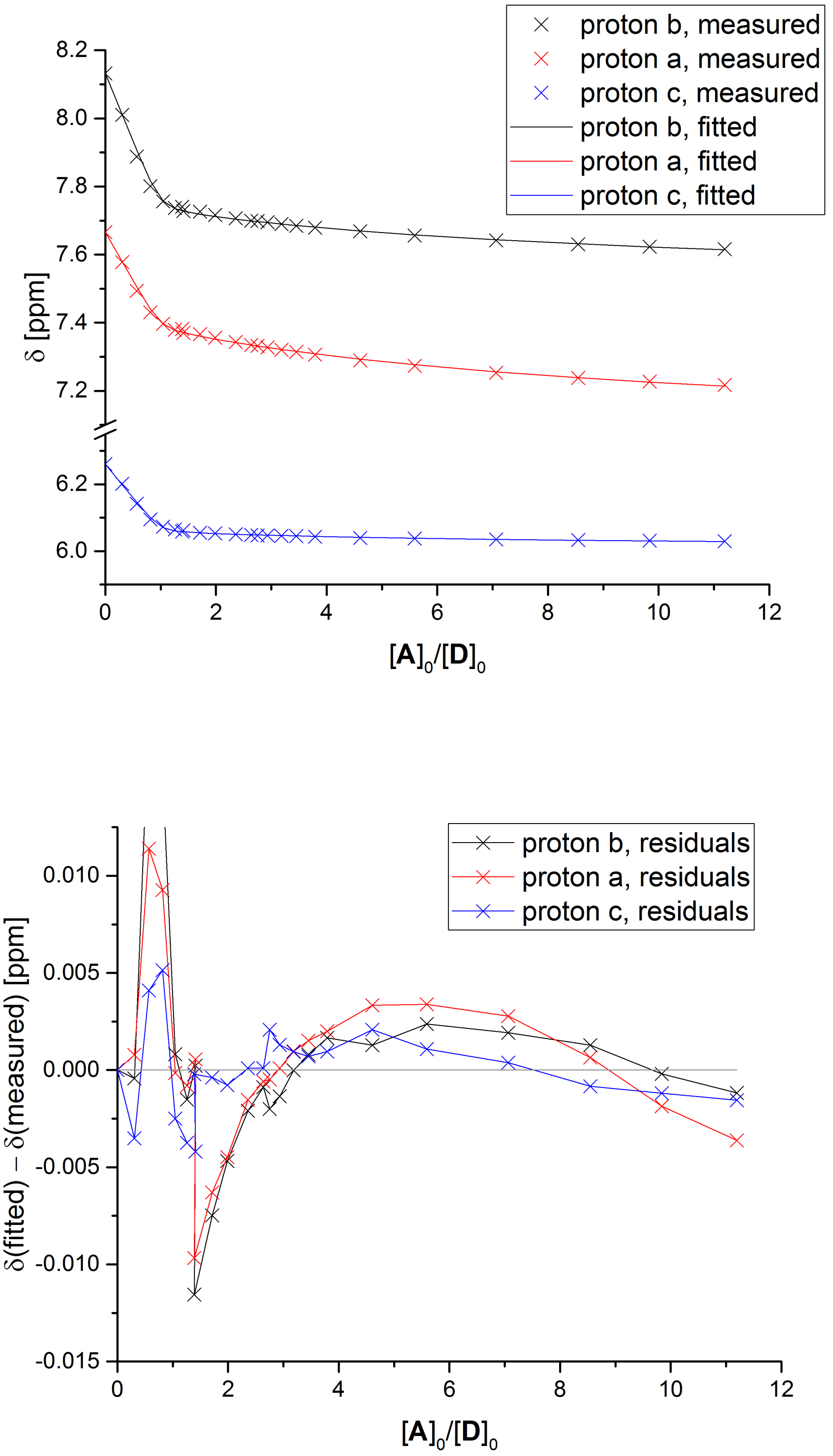

Figure S 13. Binding isotherms of the titration experiment between 1b and 2b, fitted to a 1:2 binding model, and the corresponding residuals between fitted and measured values (Experiment 3).

3.3 Titration of pyridinate 1b with silver pyrazolate 2c

3.3.1 Data of experiment 1

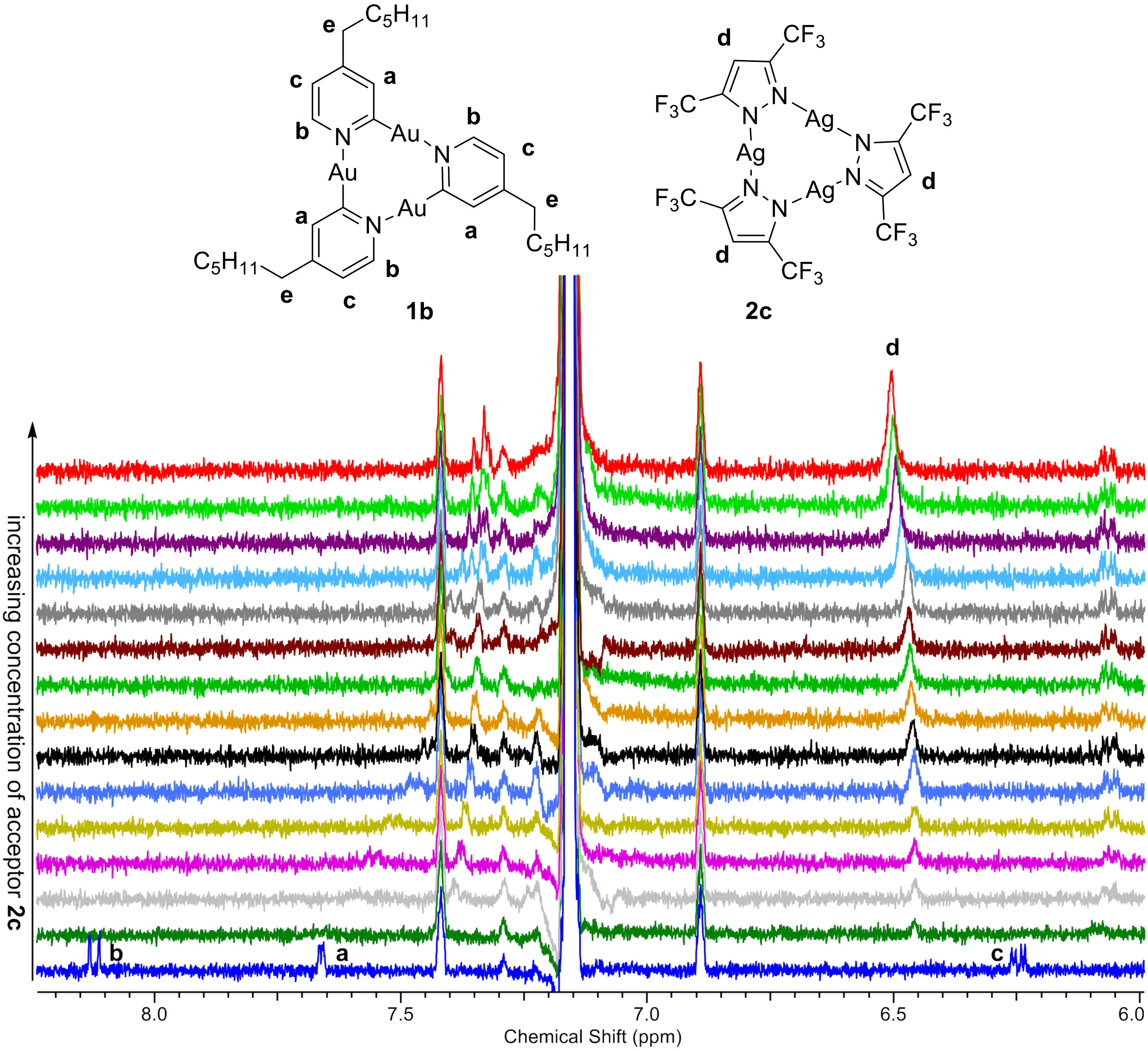

Figure S 14. ^{1}H NMR spectra for the titration of 1b with 2c (Experiment 1).

Table S 8. Measured data of the titration experiment between 1b and 2c, fitted to a non-linear 1:2 binding model (Experiment 1).

K_{11} [L mol^{-1}]	K_{12} [L mol^{-1}]		δ_H [ppm]	δ_{HG} [ppm]	δ_{HG2} [ppm]	RMS	Cov
2.91×10^{5}	1.42×10^{3}	proton b					
**K_{11} error [%]**	**K_{12} error [%]**	proton a	7.662	7.357	7.289	2.26×10^{-3}	7.75×10^{-4}
19.8	15.9	proton c					

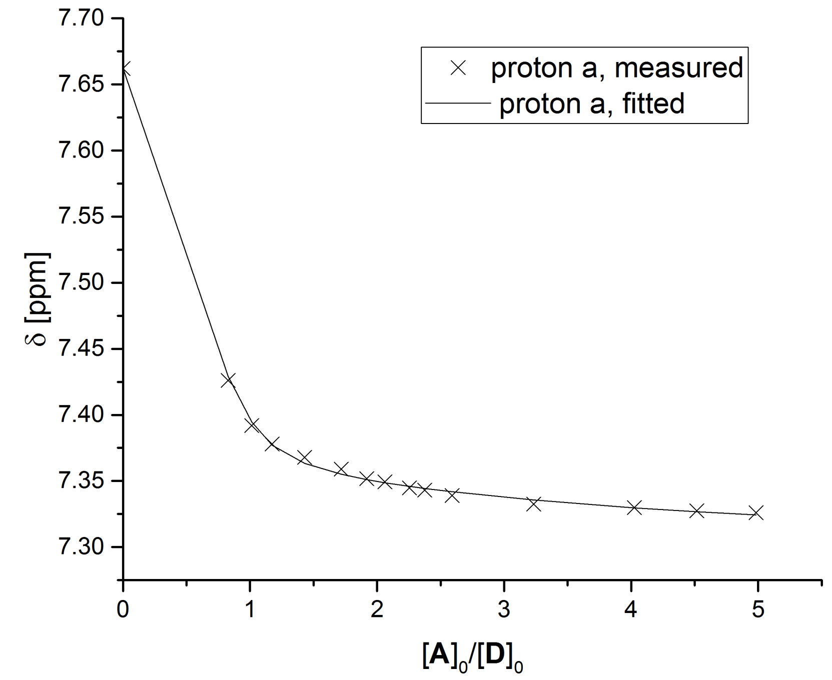

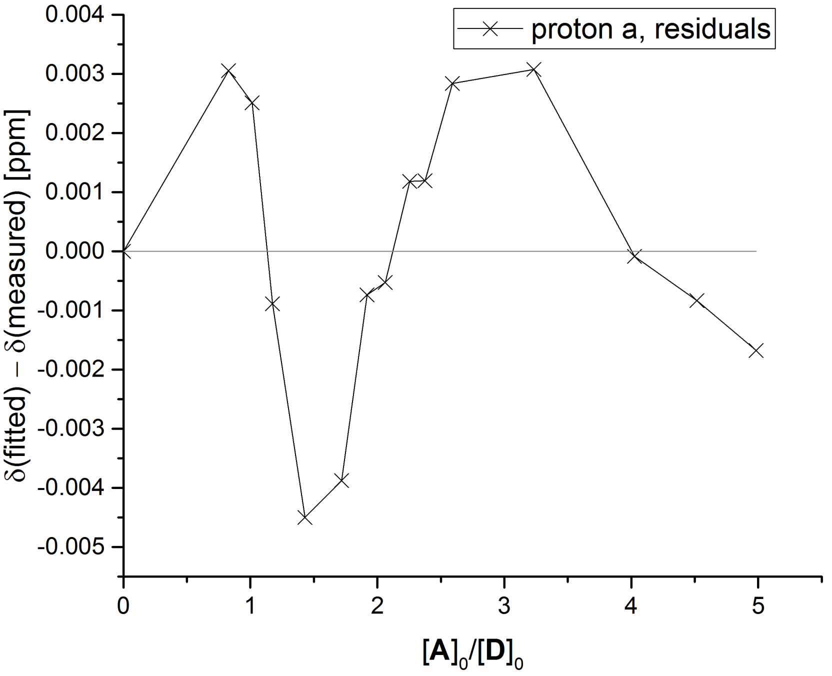

Figure S 15. Binding isotherms of the titration experiment between 1b and 2c, fitted to a 1:2 binding model, and the corresponding residuals between fitted and measured values (Experiment 1).

3.3.2 Data of experiment 2

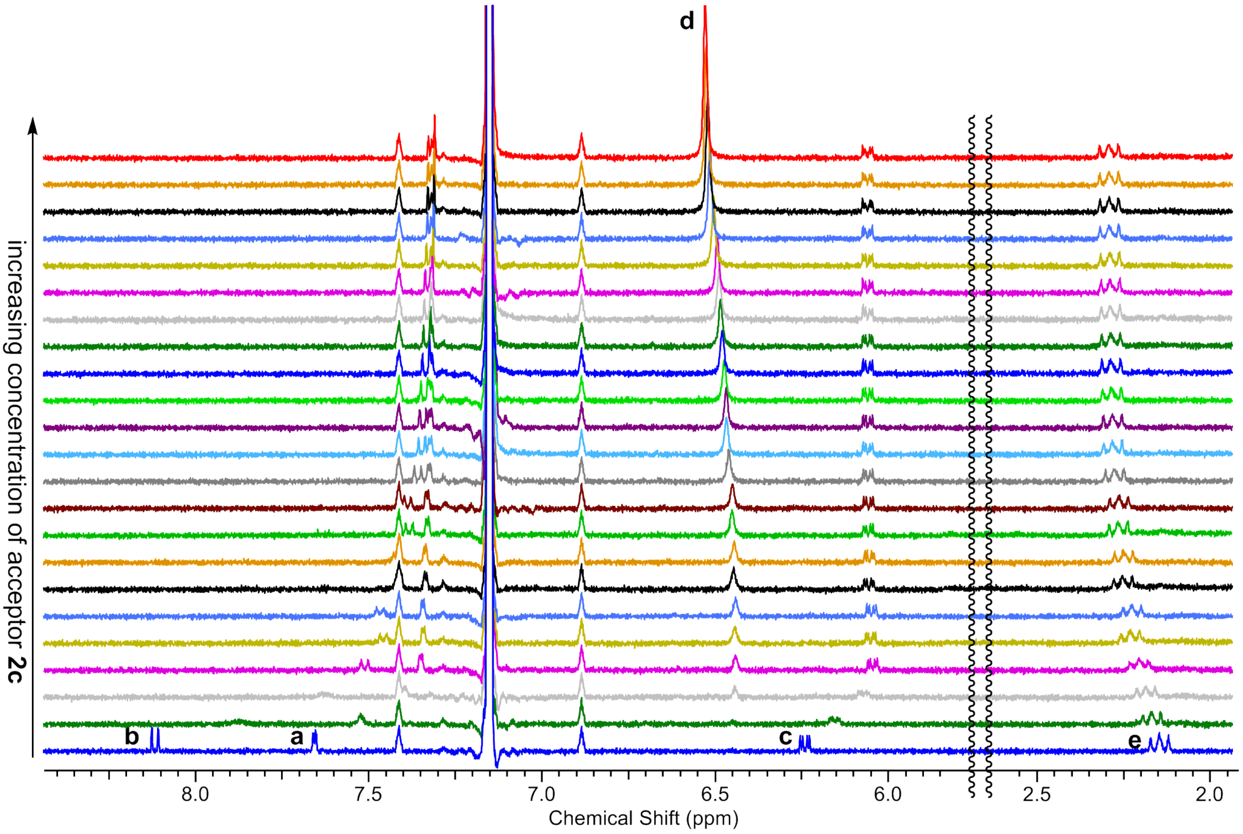

Figure S 16. **^{1}H NMR spectra for the titration of 1b with 2c (Experiment 2).**

Table S 9. Measured data of the titration experiment between 1b and 2c, fitted to a non-linear 1:2 binding model (Experiment 2).

K_{11} [L/mol^{-1}]	K_{12} [L/mol^{-1}]		δ_H [ppm]	δ_{HG} [ppm]	δ_{HG2} [ppm]	RMS	Cov
2.90×10^5	5.12×10^3	proton b	8.126	7.441	7.321	1.35×10^{-2}	4.90×10^{-2}
**K_{11} error [%]**	**K_{12} error [%]**	proton a	7.661	7.317	7.324	1.23×10^{-2}	2.45×10^{-2}
36.7	26.9	proton c	6.254	6.082	6.082	9.58×10^{-3}	5.25×10^{-2}
		proton e	2.172	2.245	2.329	5.34×10^{-3}	1.55×10^{-2}
		total				1.07×10^{-2}	1.21×10^{-3}

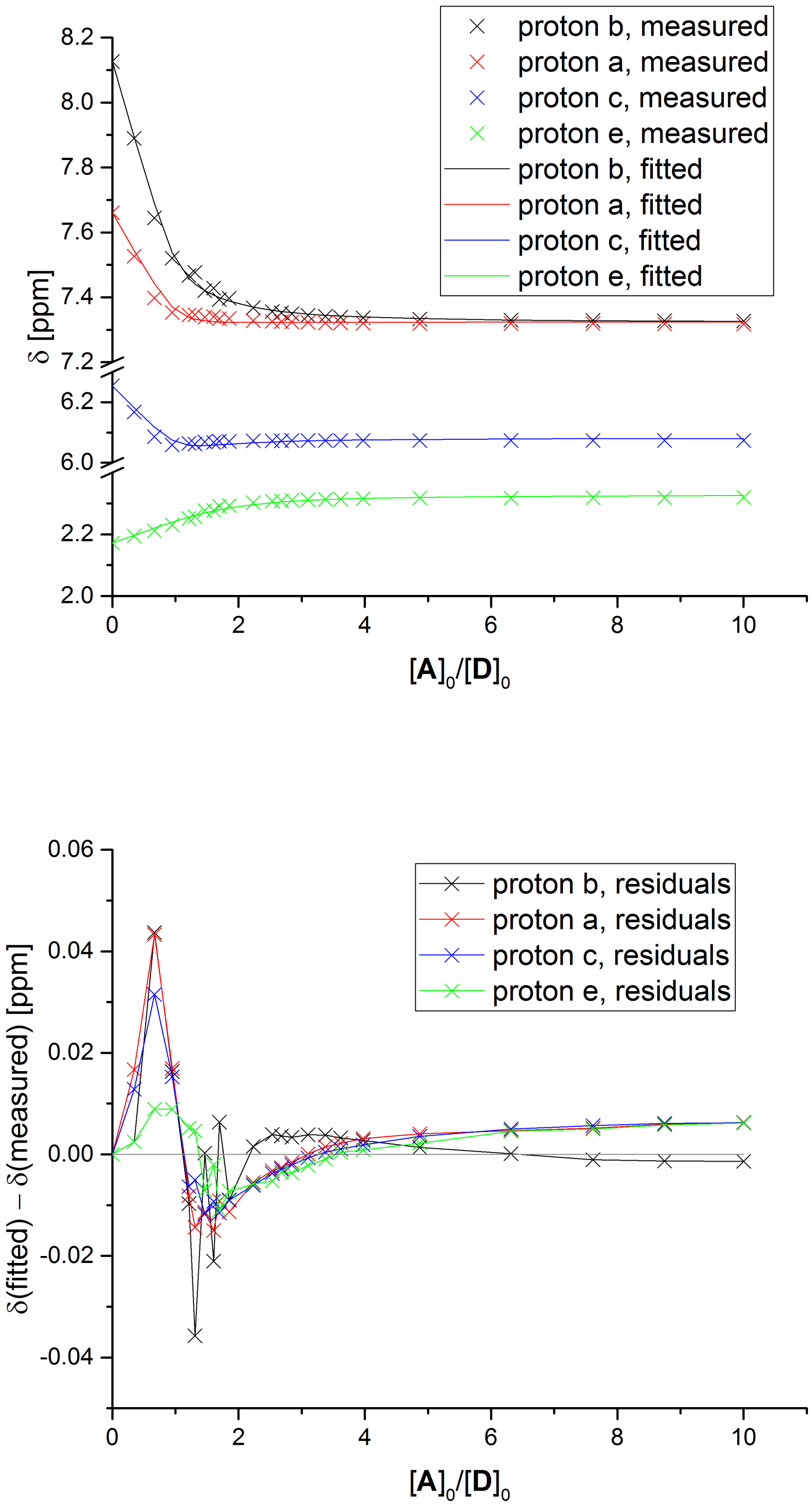

Figure S 17. Binding isotherms of the titration experiment between 1b and 2c, fitted to a 1:2 binding model, and the corresponding residuals between fitted and measured values (Experiment 2).

3.3.3 Data of experiment 3

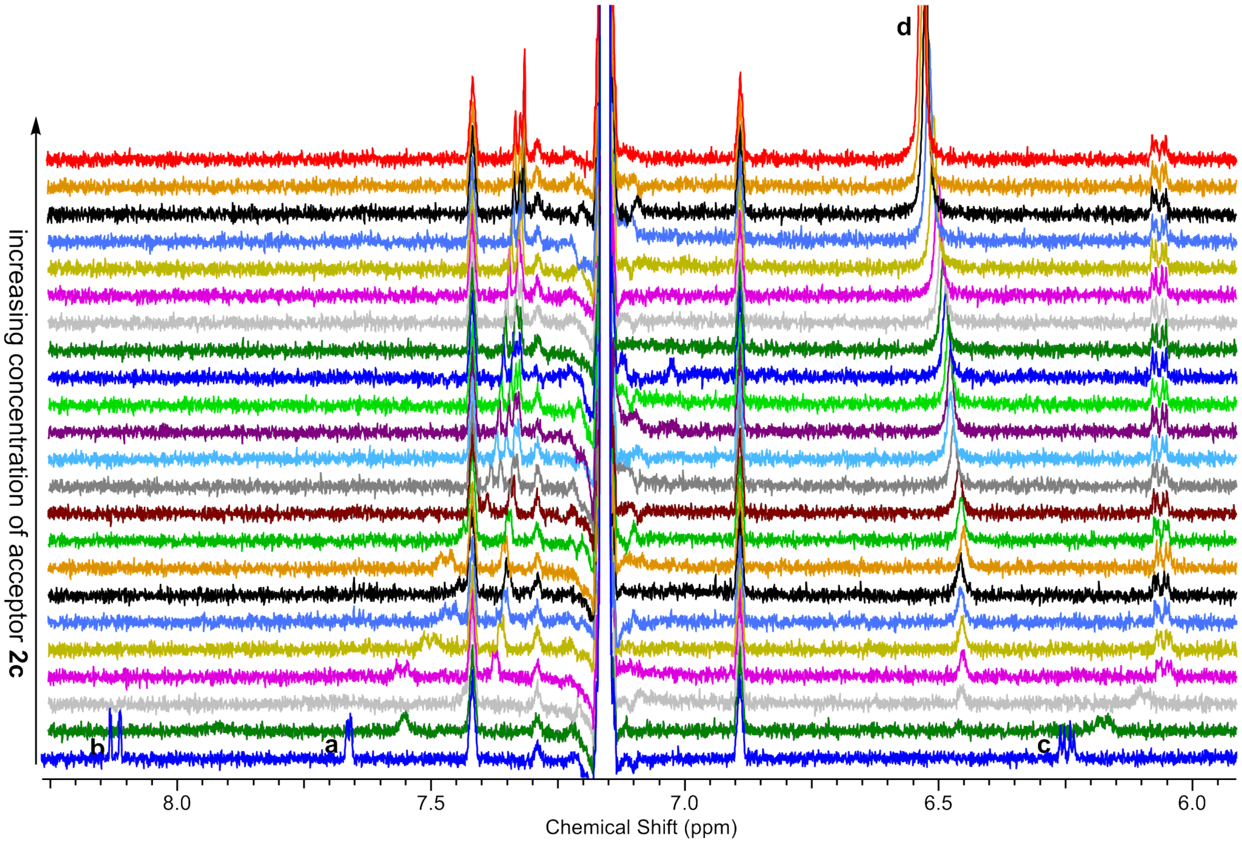

Figure S 18. **¹H NMR spectra for the titration of 1b with 2c (Experiment 3).**

Table S 10. Measured data of the titration experiment between 1b and 2c, fitted to a non-linear 1:2 binding model (Experiment 3).

K_{11} [L mol^{-1}]	K_{12} [L mol^{-1}]		δ_H [ppm]	δ_{HG} [ppm]	δ_{HG2} [ppm]	RMS	Cov
2.70×10^5	2.72×10^3	proton b	8.122	7.423	7.305	1.87×10^{-2}	8.87×10^{-3}
**K_{11} error [%]**	**K_{12} error [%]**	proton a	7.662	7.323	7.327	1.49×10^{-2}	3.38×10^{-2}
43.8	35.8	proton c	6.260	6.047	6.093	1.20×10^{-2}	7.70×10^{-2}
		total				1.54×10^{-2}	3.89×10^{-3}

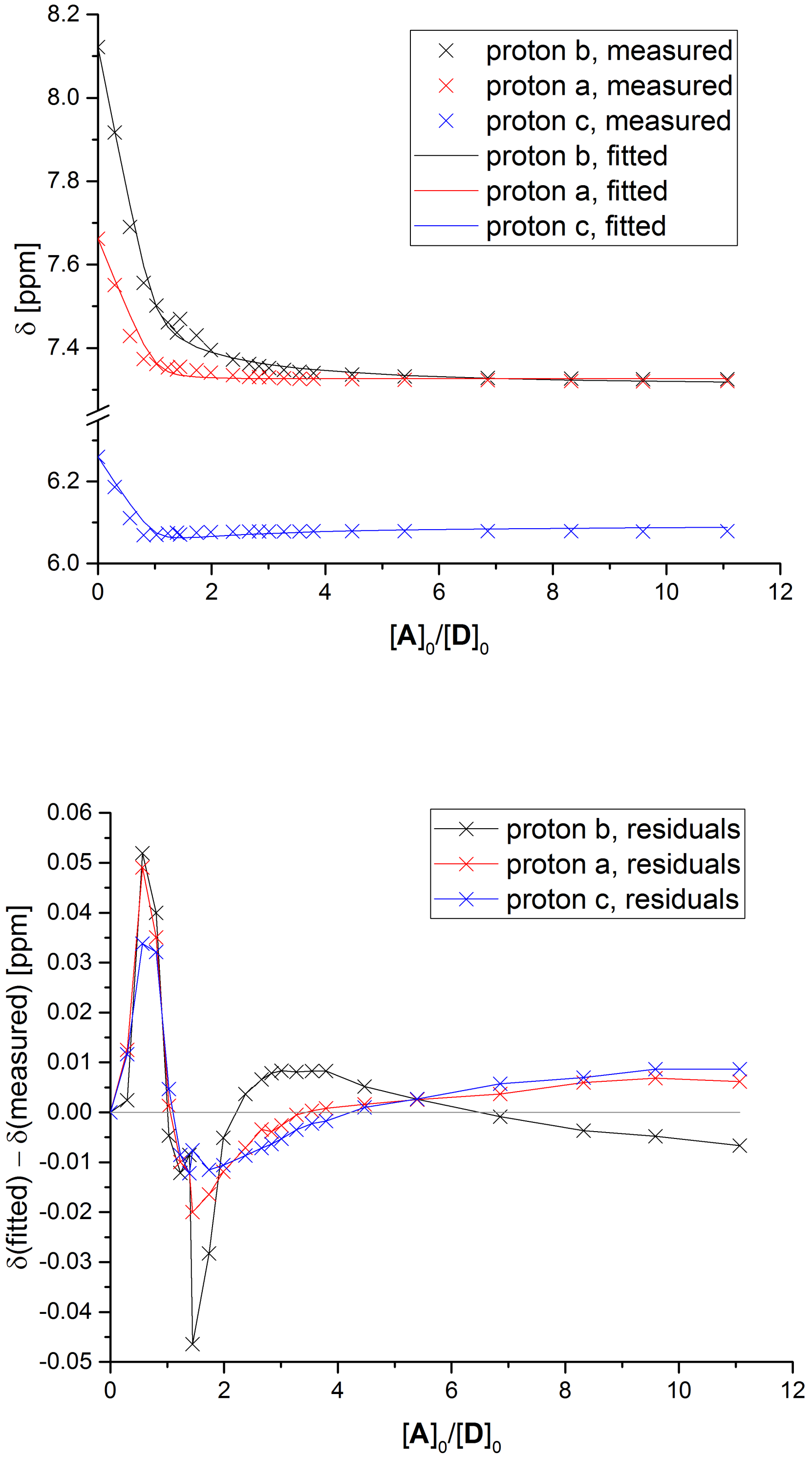

Figure S 19. Binding isotherms of the titration experiment between 1b and 2c, fitted to a 1:2 binding model, and the corresponding residuals between fitted and measured values (Experiment 3).

3.4 Titration of pyridinate 1d with silver pyrazolate 2c

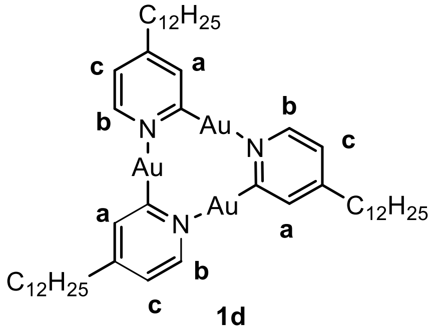

1d

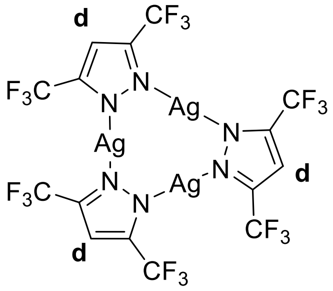

2c

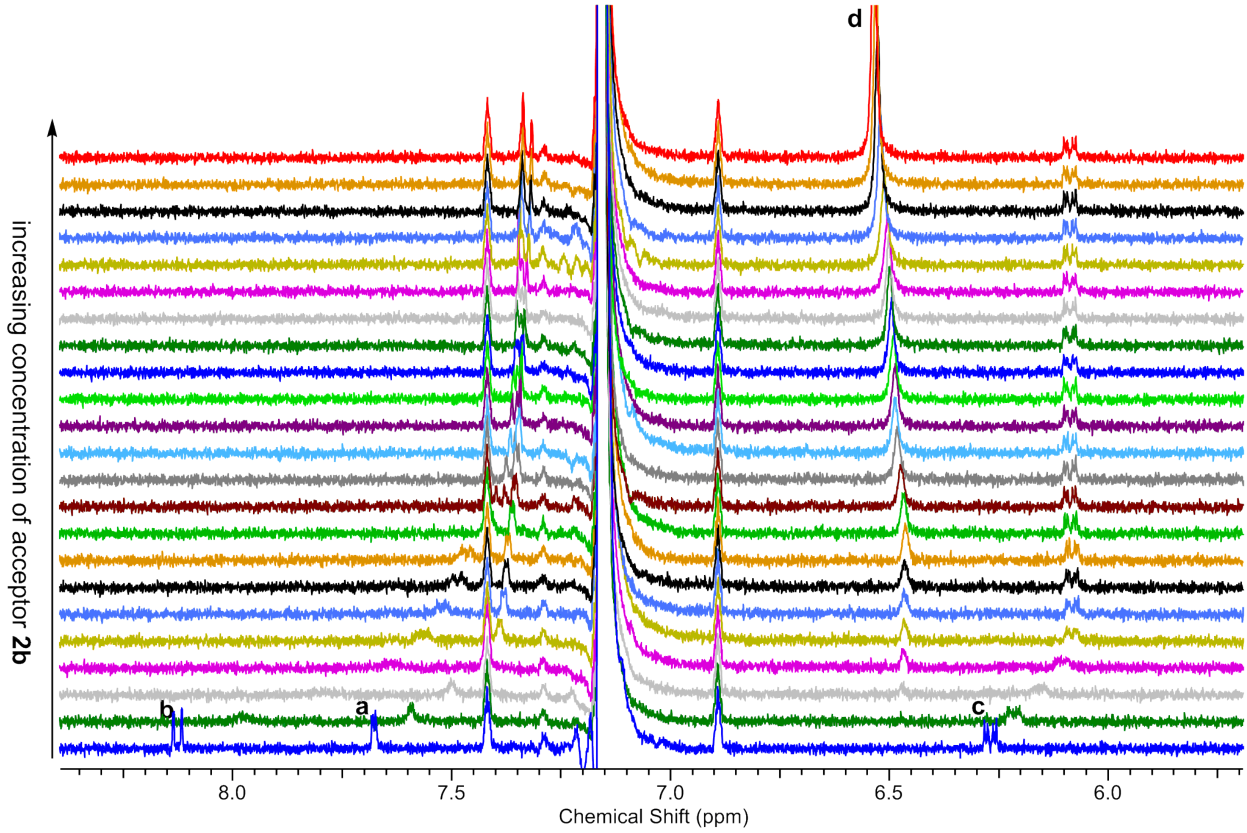

Figure S 20. ^{1}**H NMR spectra for the titration of 1d with 2c.**

Table S 11. Measured data of the titration experiment between 1d and 2c, fitted to a non-linear 1:2 binding model.

**K_{11} [L mol^{-1}]**	**K_{12} [L mol^{-1}]**		**δ_H [ppm]**	**δ_{HG} [ppm]**	**δ_{HG2} [ppm]**	**RMS**	**Cov**
5.81×10^5	9.43×10^3	proton b	8.136	7.473	7.331	1.52×10^{-2}	4.95×10^{-3}
**K_{11} error [%]**	**K_{12} error [%]**	proton a	7.677	7.342	7.344	8.99×10^{-3}	1.10×10^{-2}
32.3	32.7	proton c	6.282	6.062	6.108	6.80×10^{-3}	2.21×10^{-2}
		total				1.09×10^{-2}	1.91×10^{-3}

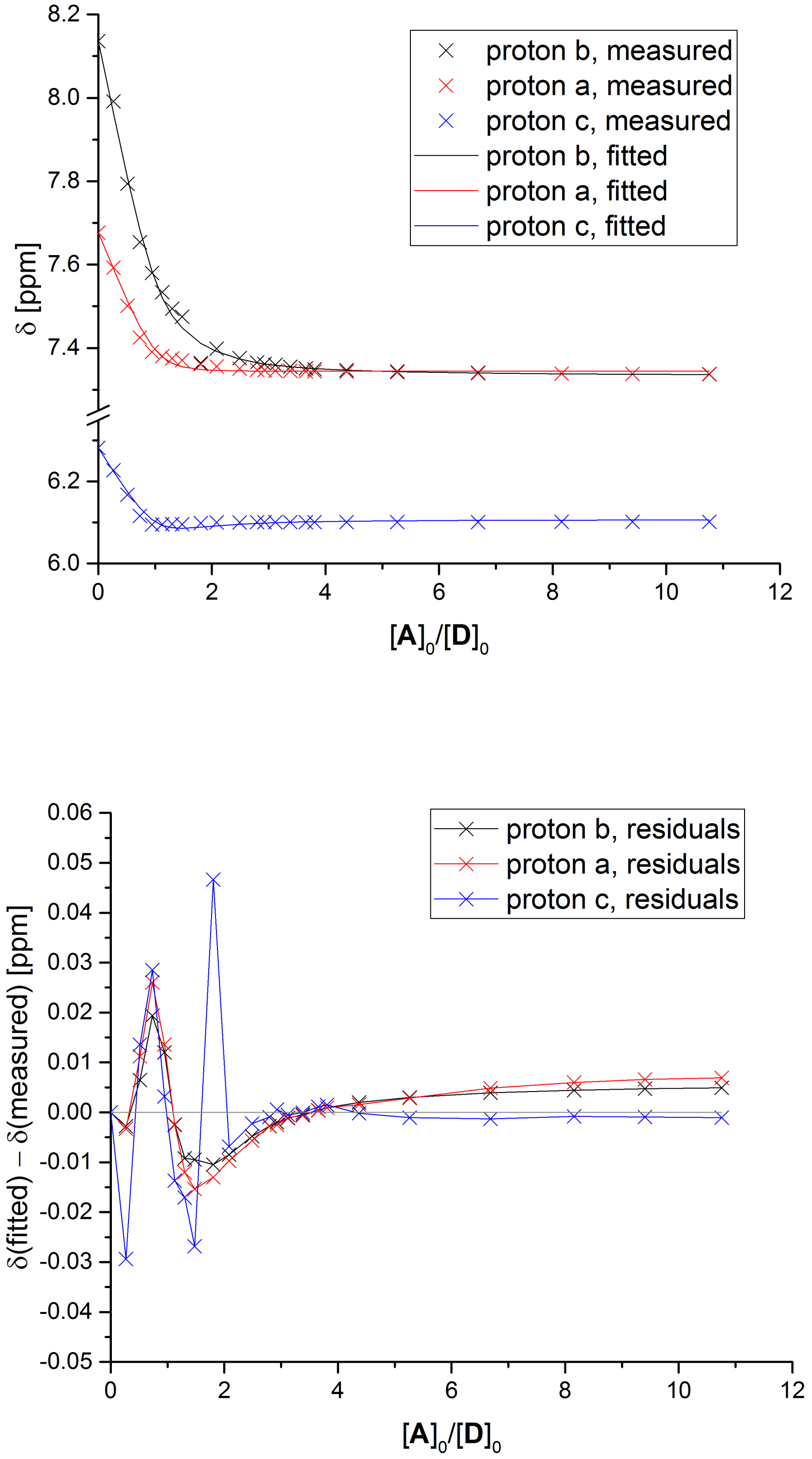

Figure S 21. Binding isotherms of the titration experiment between 1b and 2c, fitted to a 1:2 binding model, and the corresponding residuals between fitted and measured values.

4 Stability of pyridinate 1b

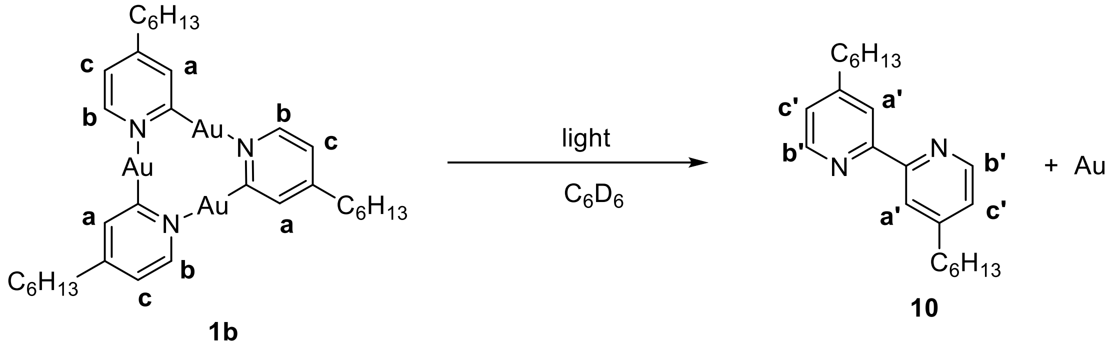

Scheme S 1. Decomposition of pyridinate 1b in solution under sunlight exposition, forming 2,2'-bipyridine 10 and colloidal gold.

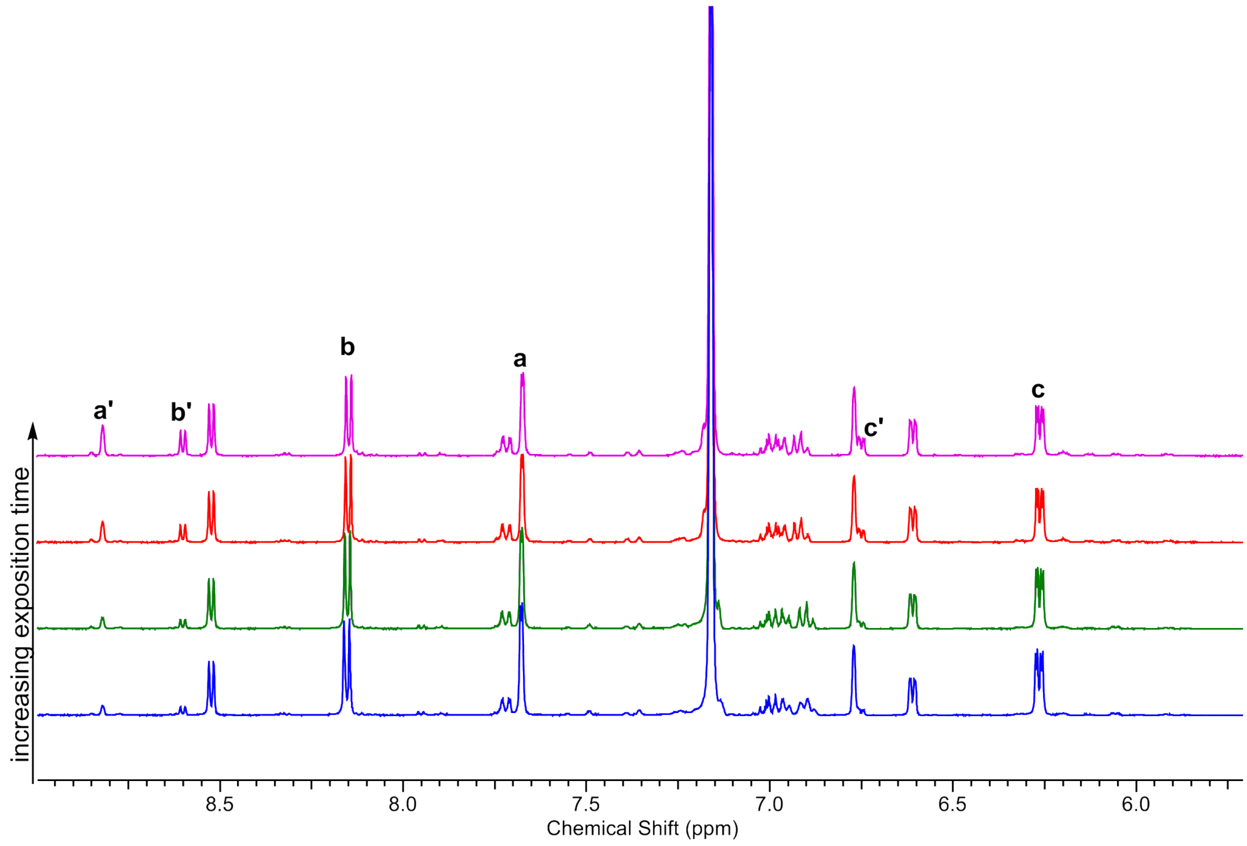

Figure S 22. ^{1}H NMR spectra of pyridinate 1b over a period of three days, showing successive decomposition to 2,2'-bipyridine 10 due to sunlight exposure.

5 NMR spectra

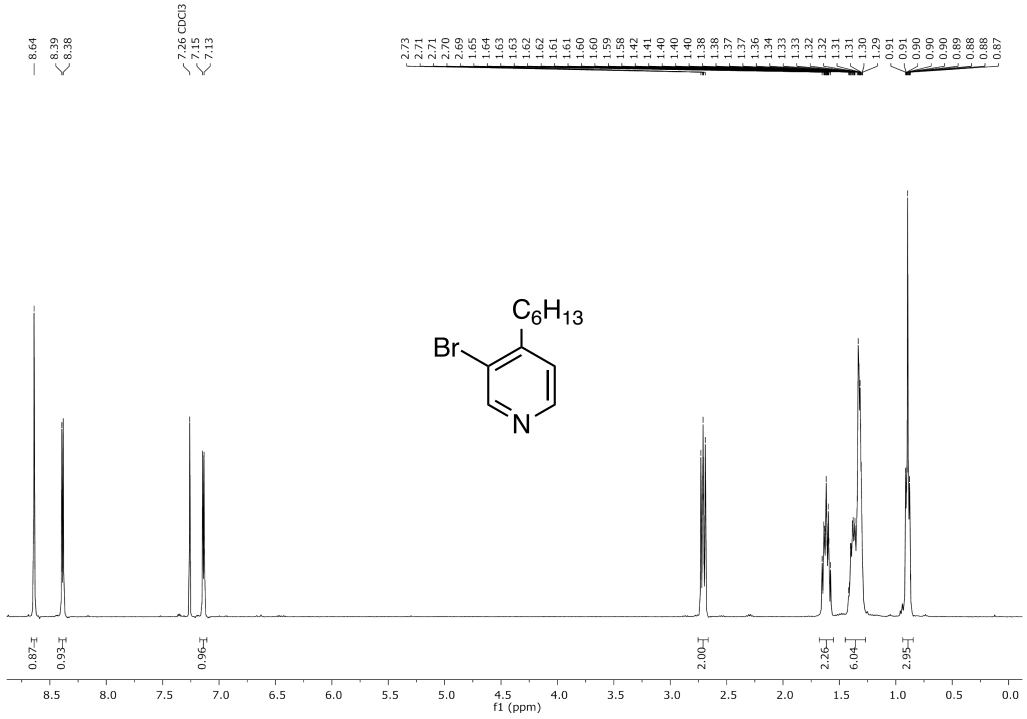

Figure S 23. ^{1}H NMR spectrum of 5a in $CDCl_3$ (400 MHz).

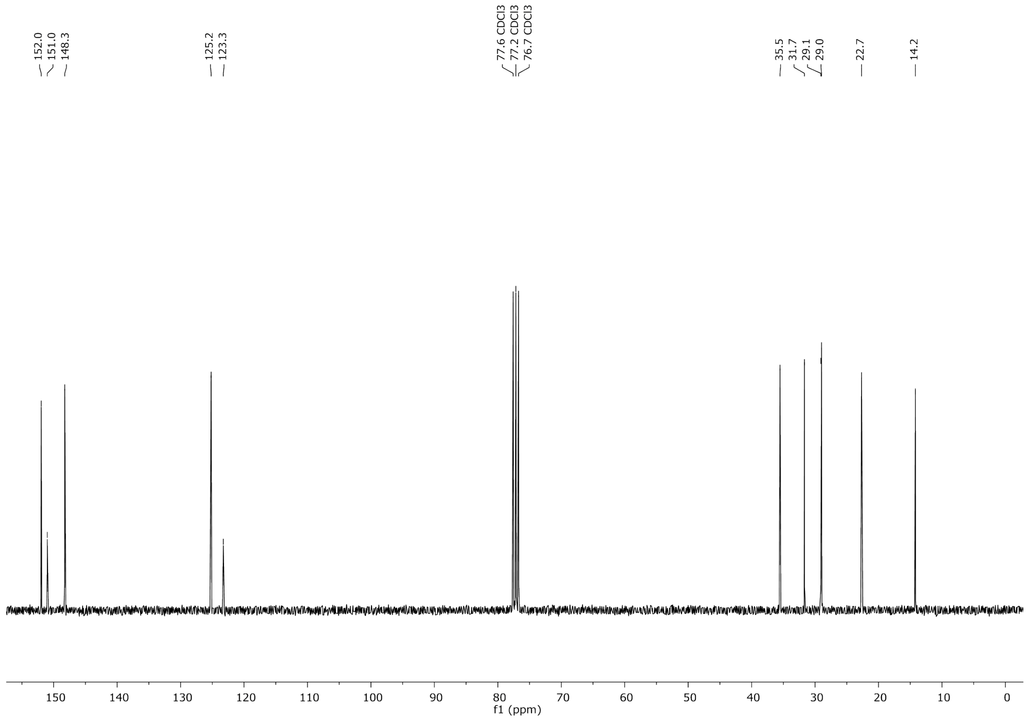

Figure S 24. ^{13}C NMR spectrum of 5a in $CDCl_3$ (101 MHz).

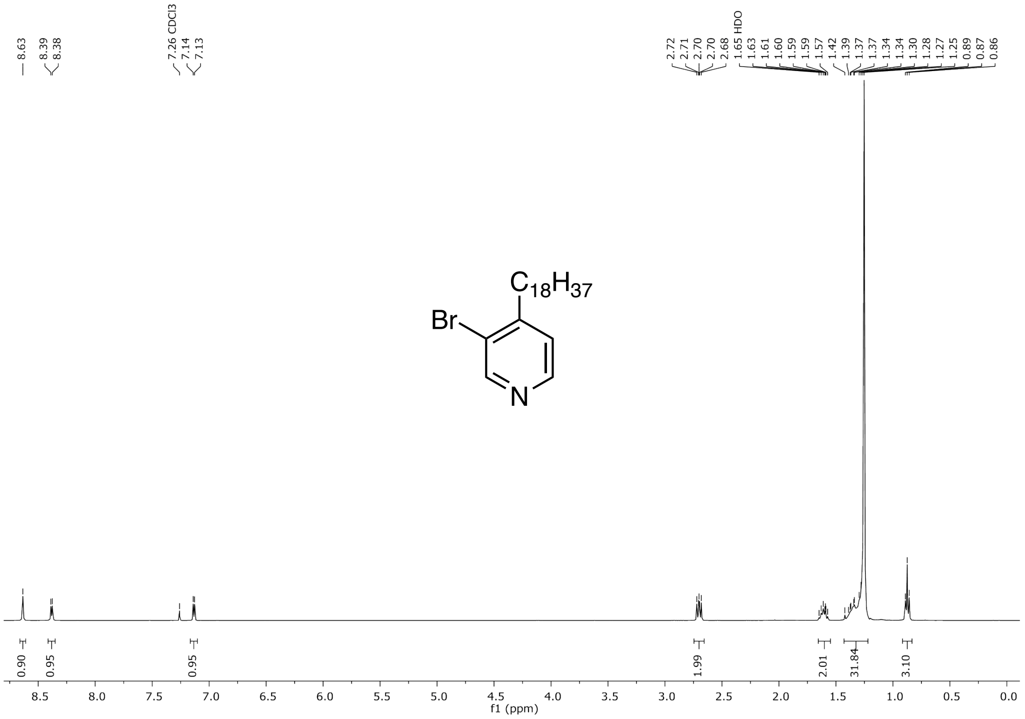

Figure S 25. ^{1}H NMR spectrum of 5b in $CDCl_3$ (500 MHz).

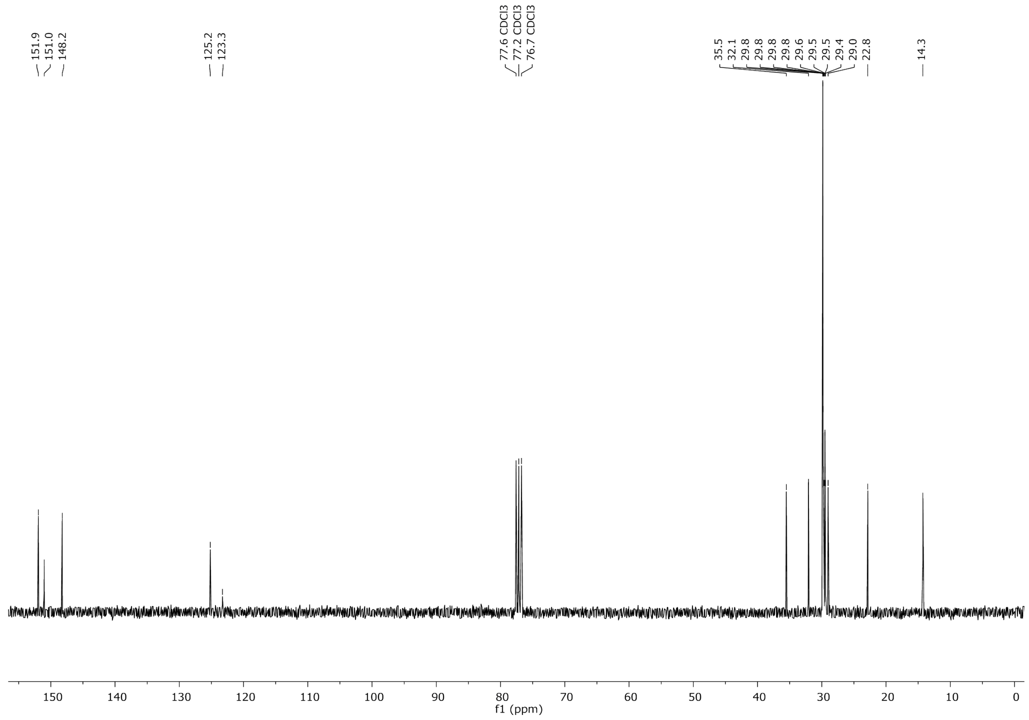

Figure S 26. ^{13}C NMR spectrum of 5b in $CDCl_3$ (126 MHz).

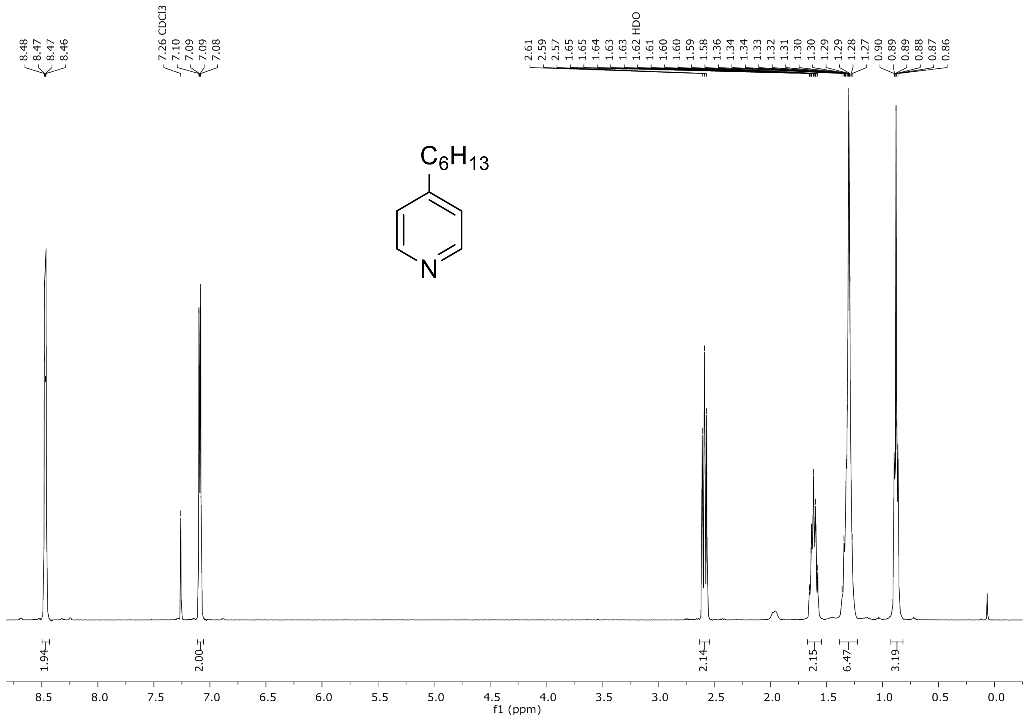

Figure S 27. **^{1}H NMR spectrum of 6b in $CDCl_3$ (400 MHz).**

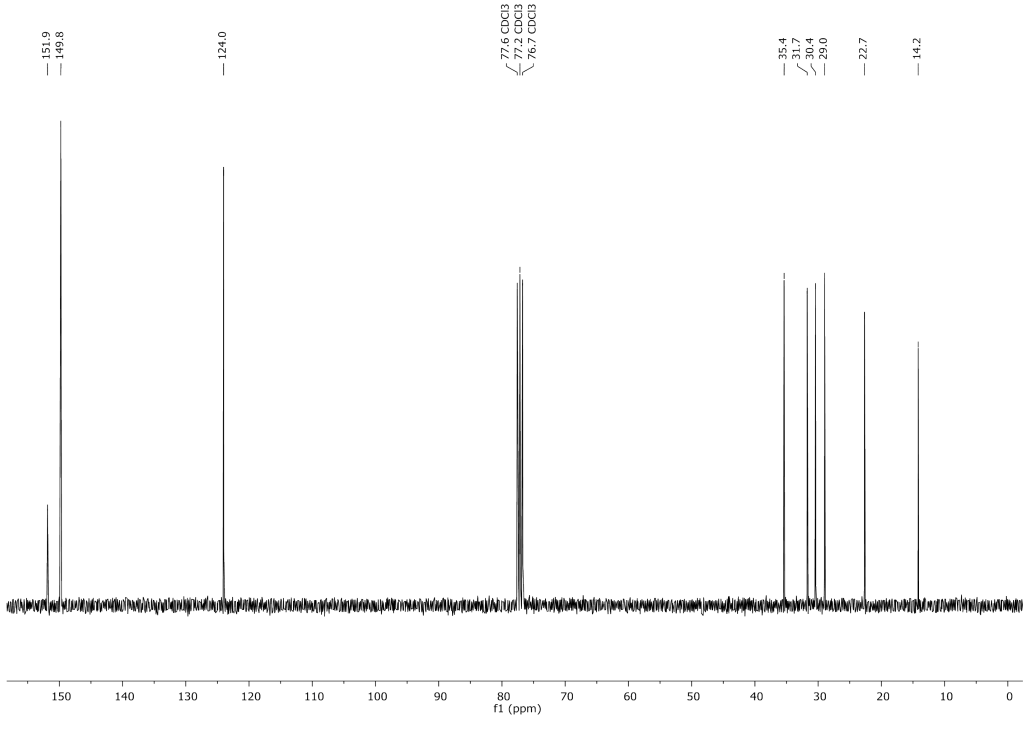

Figure S 28. **^{13}C NMR spectrum of 6b in $CDCl_3$ (76 MHz).**

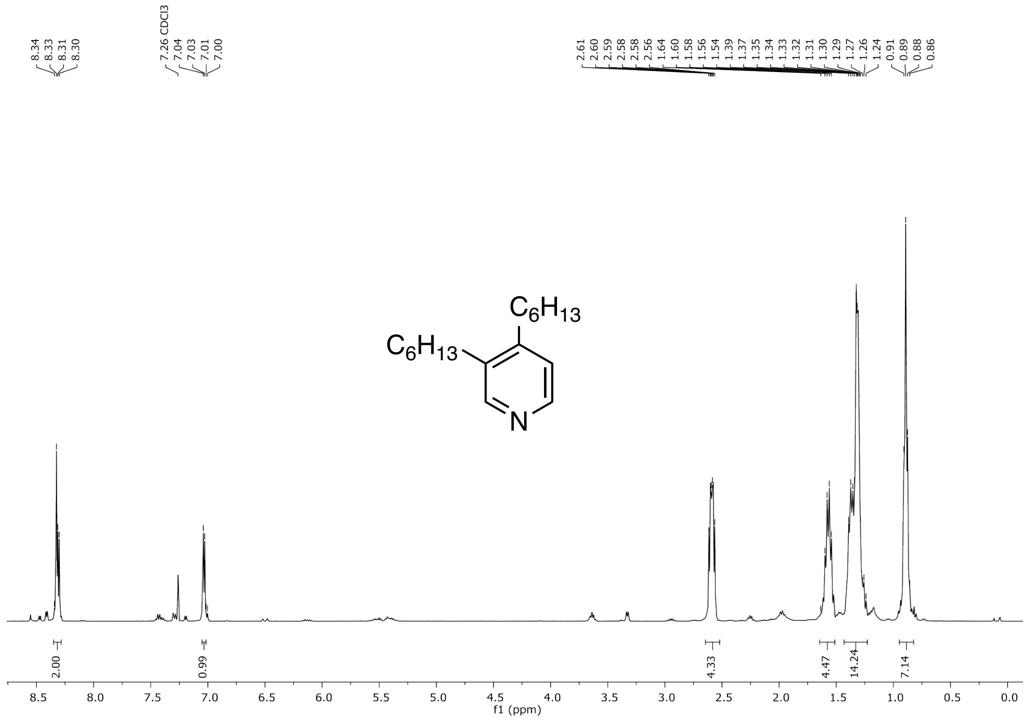

Figure S 29. ^{1}H NMR spectrum of 6c in $CDCl_3$ (400 MHz).

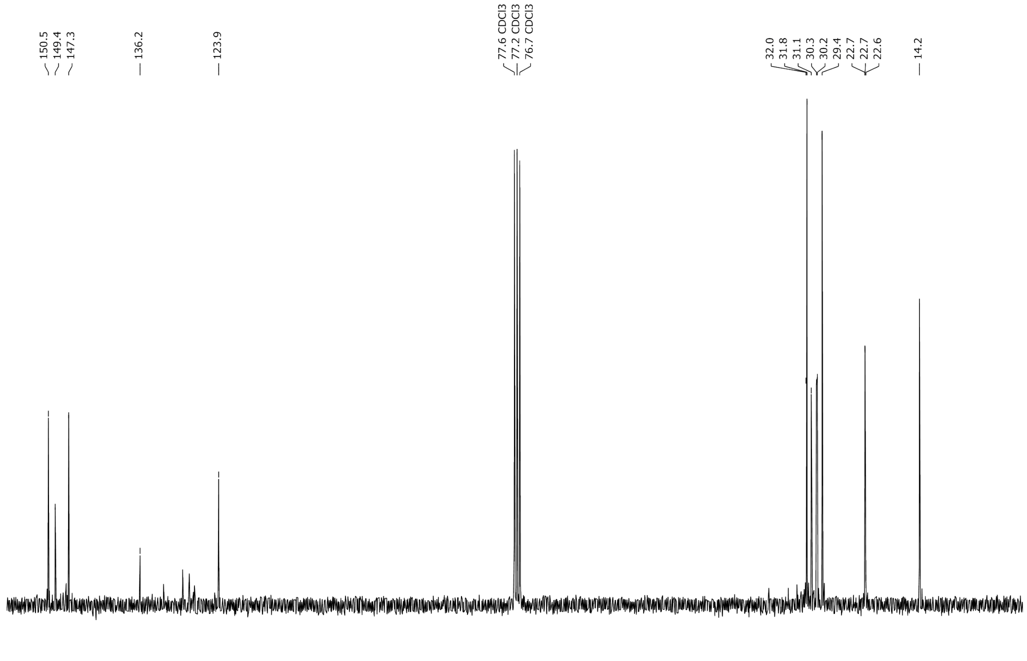

Figure S 30. ^{13}C NMR spectrum of 6c in $CDCl_3$ (76 MHz).

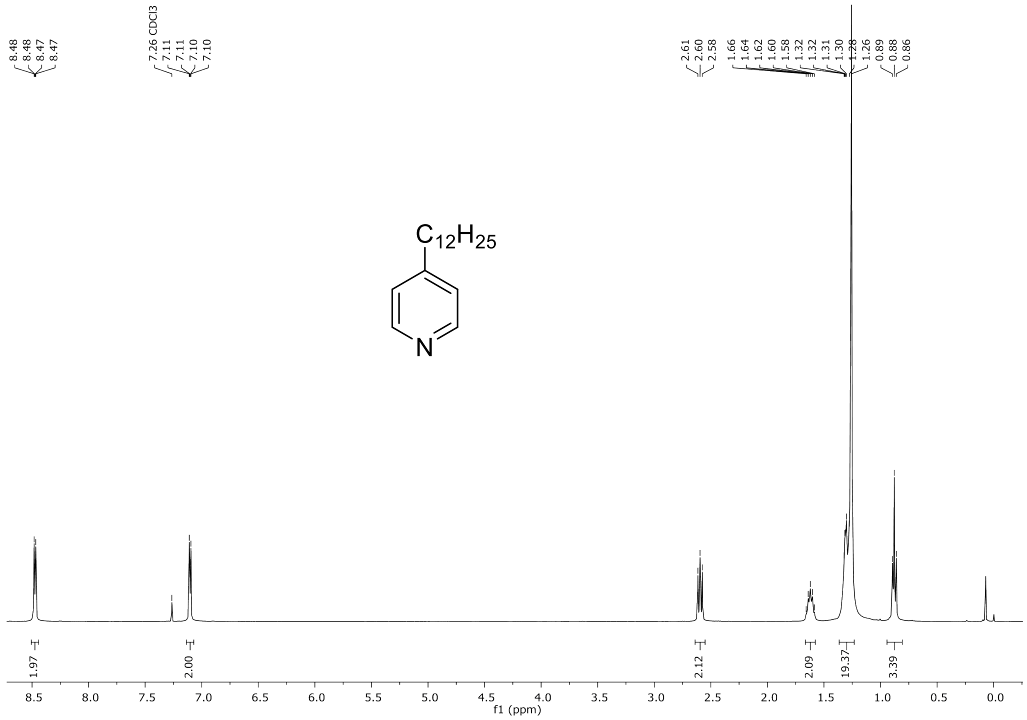

Figure S 31. ^{1}H NMR spectrum of 6d in $CDCl_3$ (400 MHz).

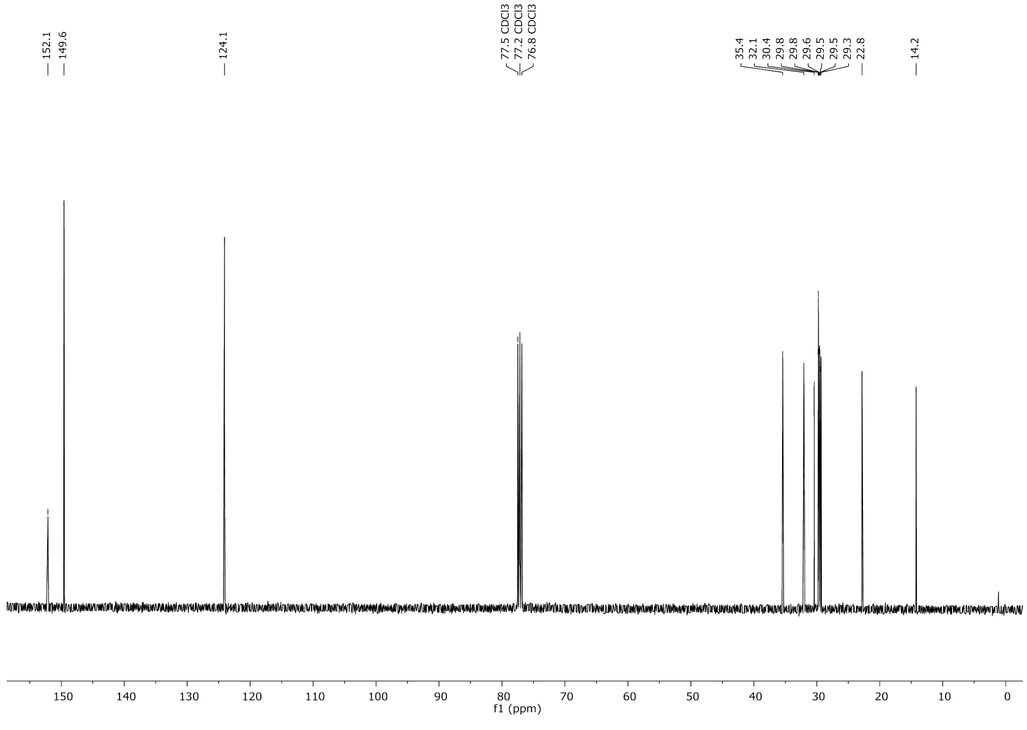

Figure S 32. ^{13}C NMR spectrum of 6d in $CDCl_3$ (101 MHz).

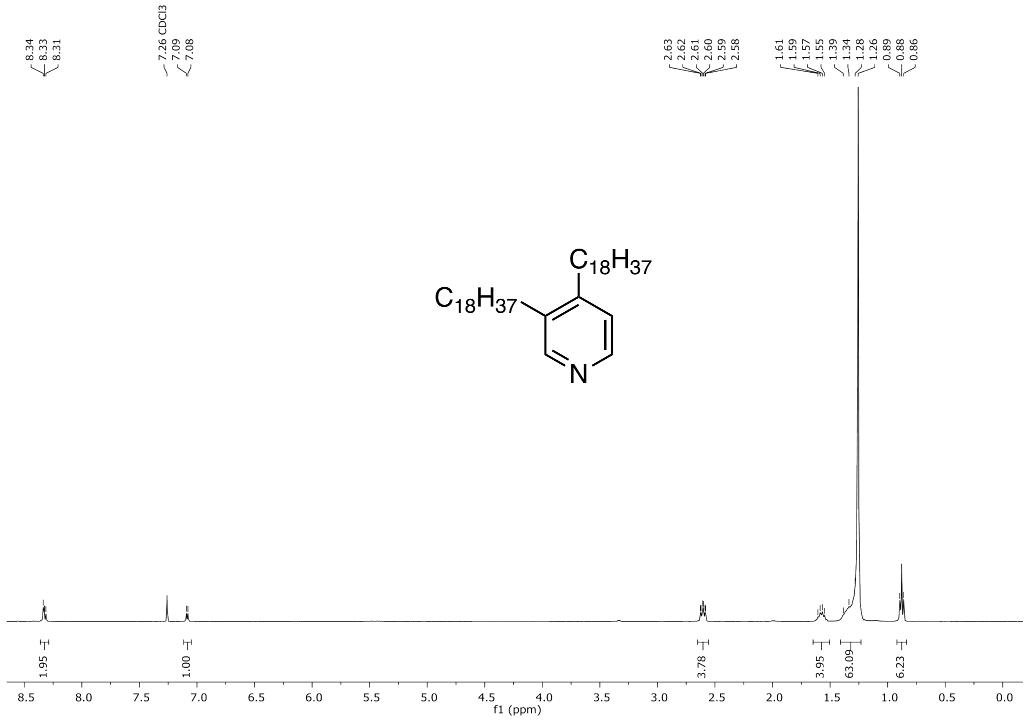

Figure S 33. ^{1}H **NMR spectrum of 6f in** $CDCl_3$ **(400 MHz).**

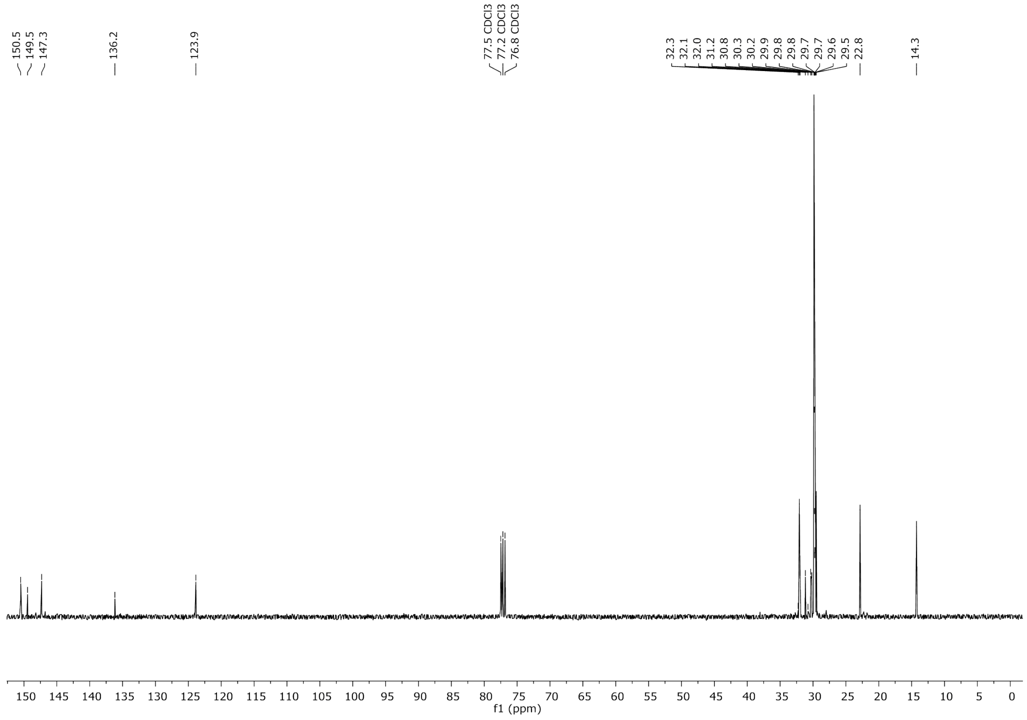

Figure S 34. ^{13}C **NMR spectrum of 6f in** $CDCl_3$ **(101 MHz).**

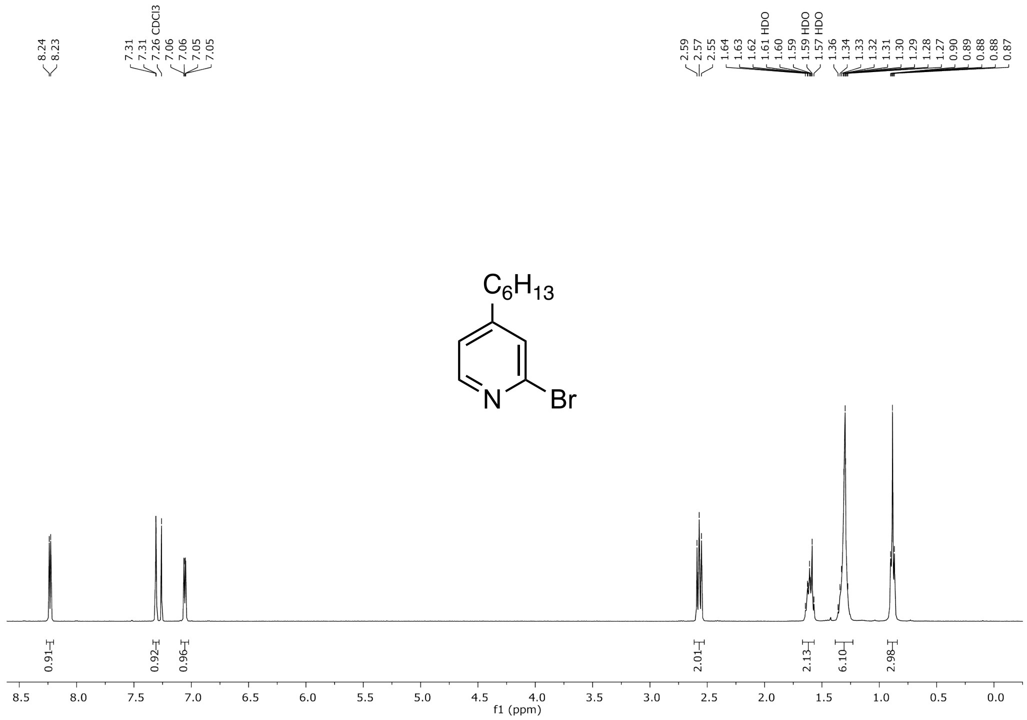

Figure S 35. ^{1}H NMR spectrum of 3b in $CDCl_3$ (400 MHz).

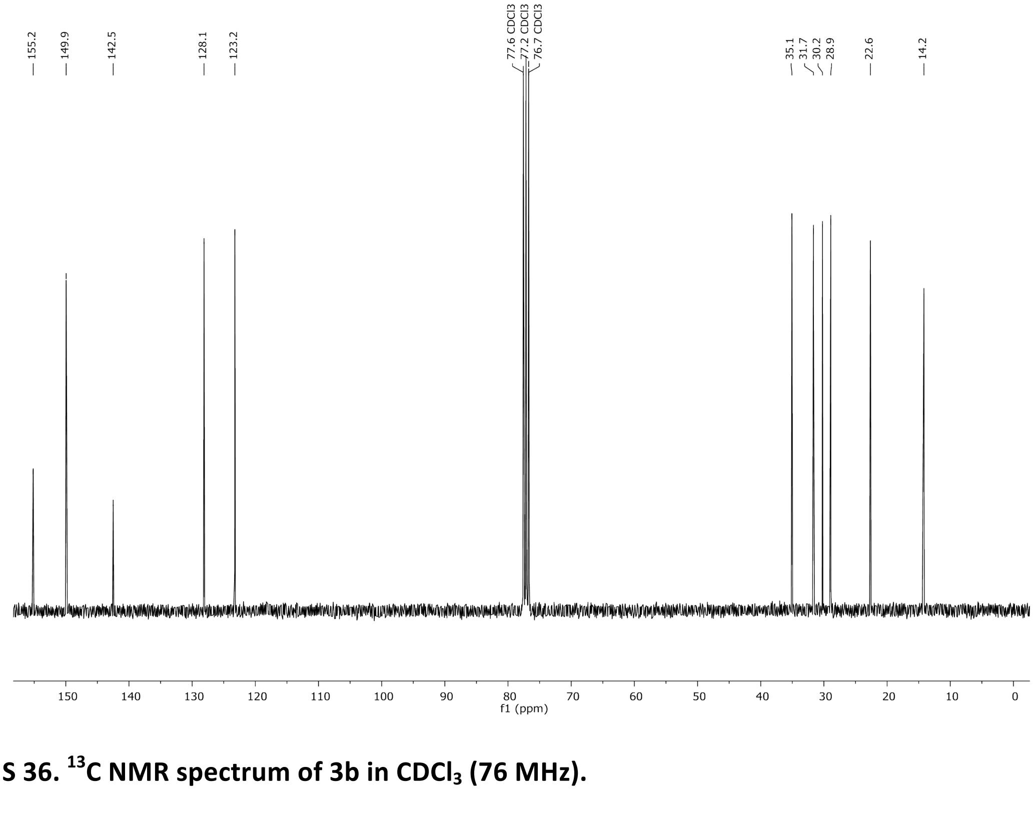

Figure S 36. ^{13}C NMR spectrum of 3b in $CDCl_3$ (76 MHz).

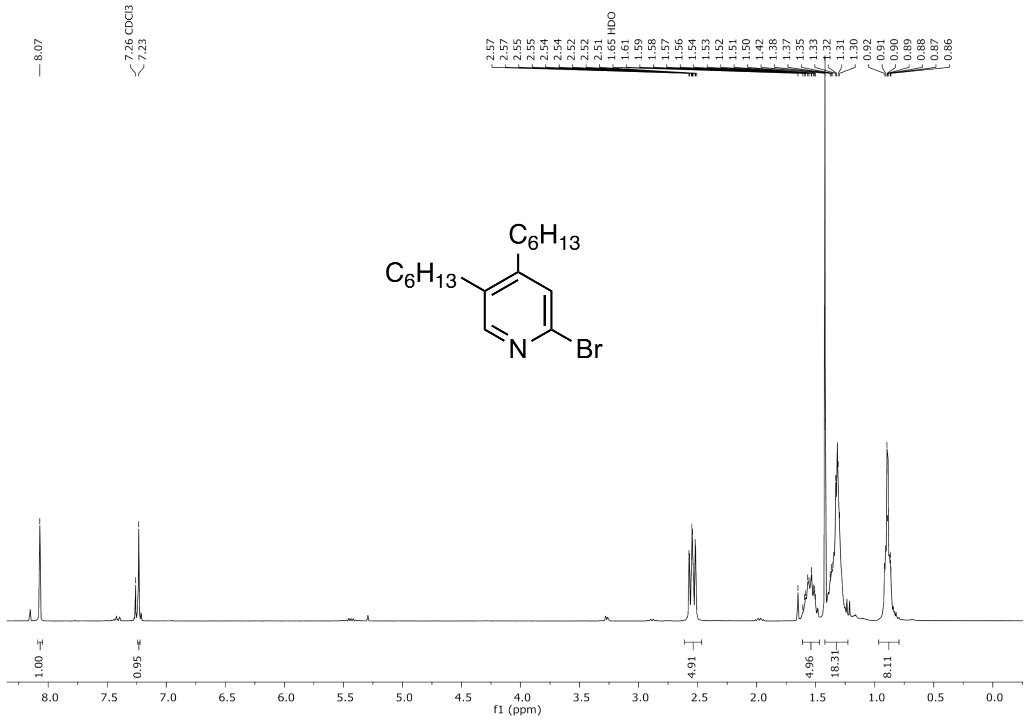

Figure S 37. **^{1}H NMR spectrum of 3c in $CDCl_3$ (300 MHz).**

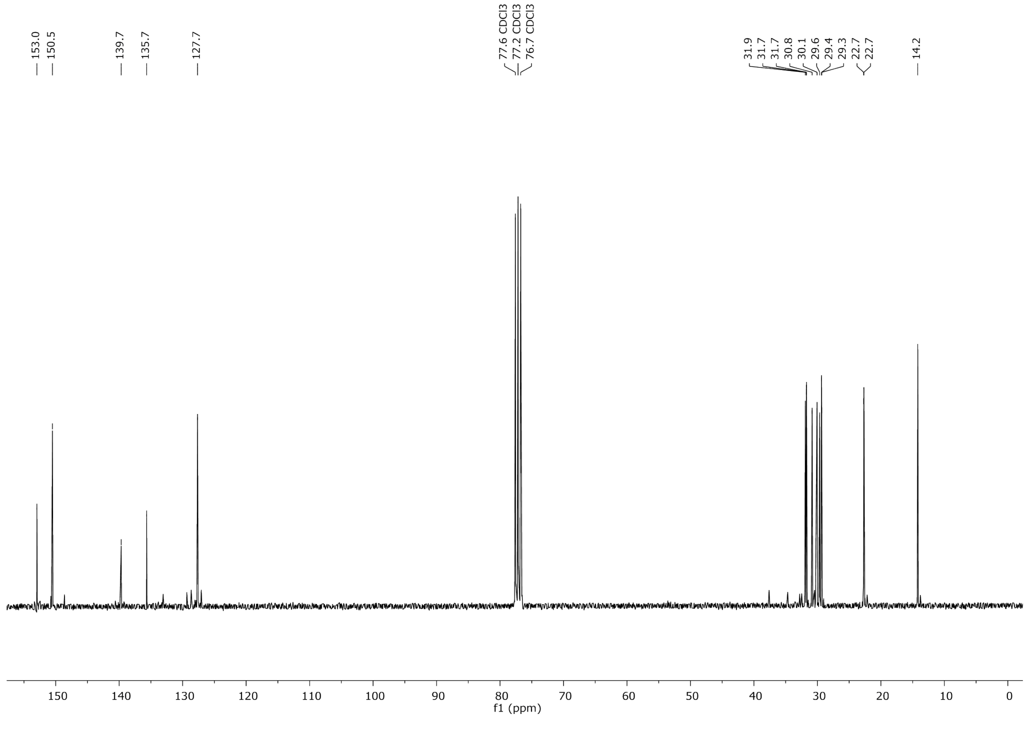

Figure S 38. **^{13}C NMR spectrum of 3c in $CDCl_3$ (76 MHz).**

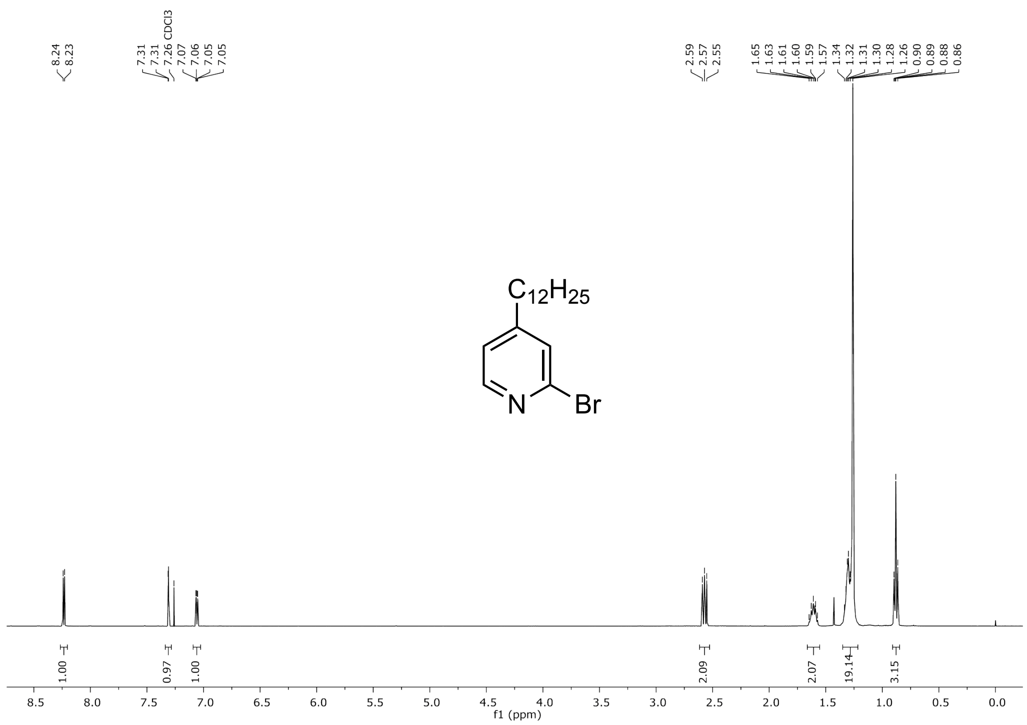

Figure S 39. ^{1}H NMR spectrum of 3d in $CDCl_3$ (400 MHz).

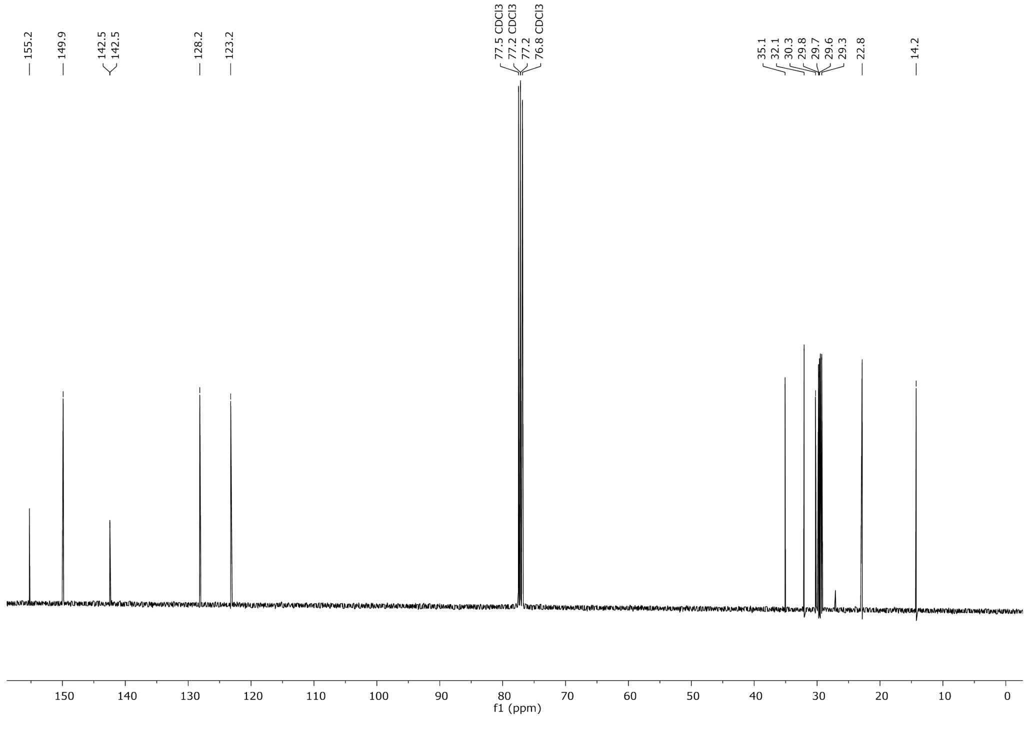

Figure S 40. ^{13}C NMR spectrum of 3d in $CDCl_3$ (101 MHz).

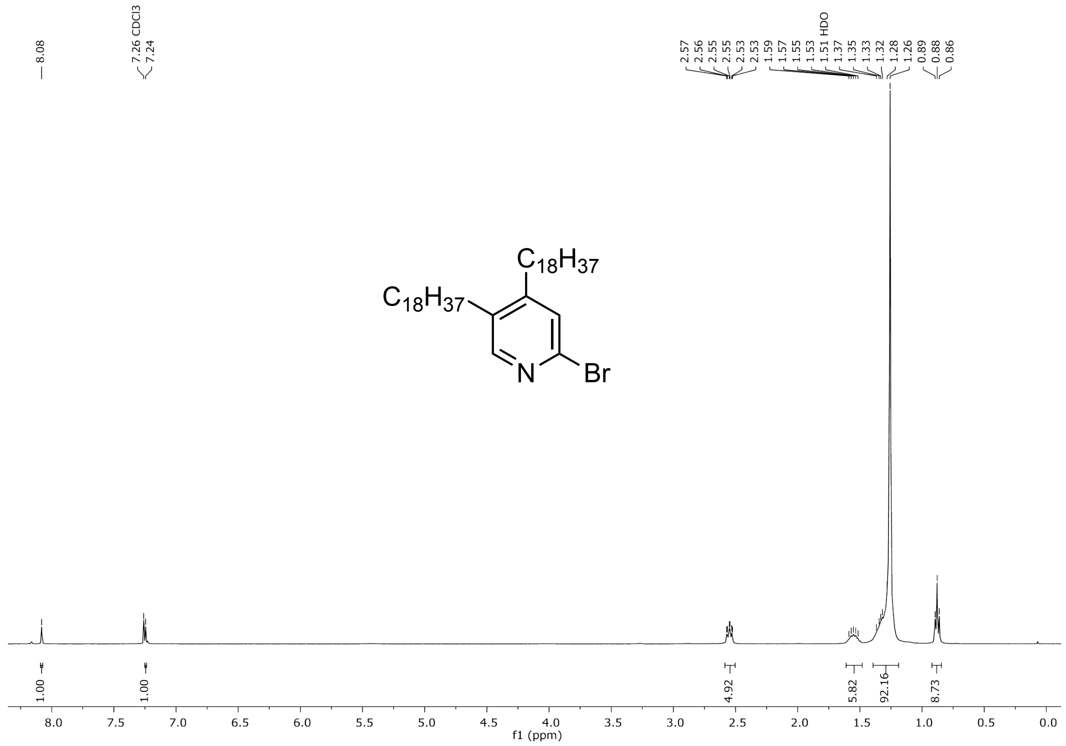

Figure S 41. ^{1}**H NMR spectrum of 3f in** $CDCl_3$ **(400 MHz).**

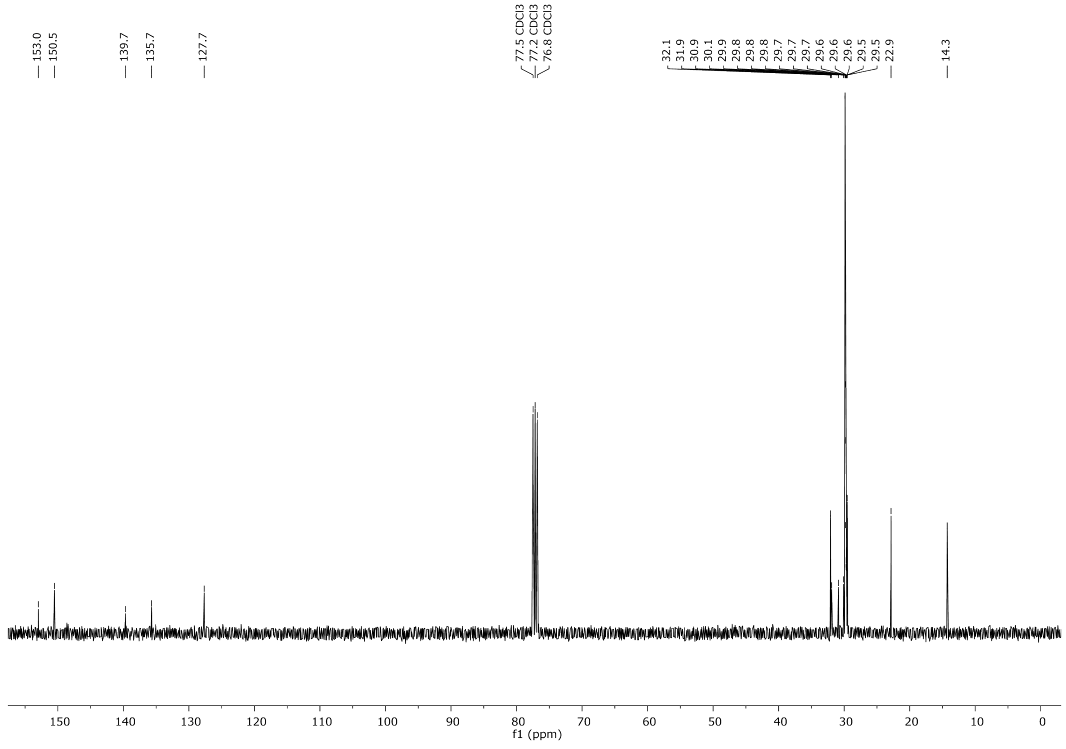

Figure S 42. ^{13}**C NMR spectrum of 3f in** $CDCl_3$ **(101 MHz).**

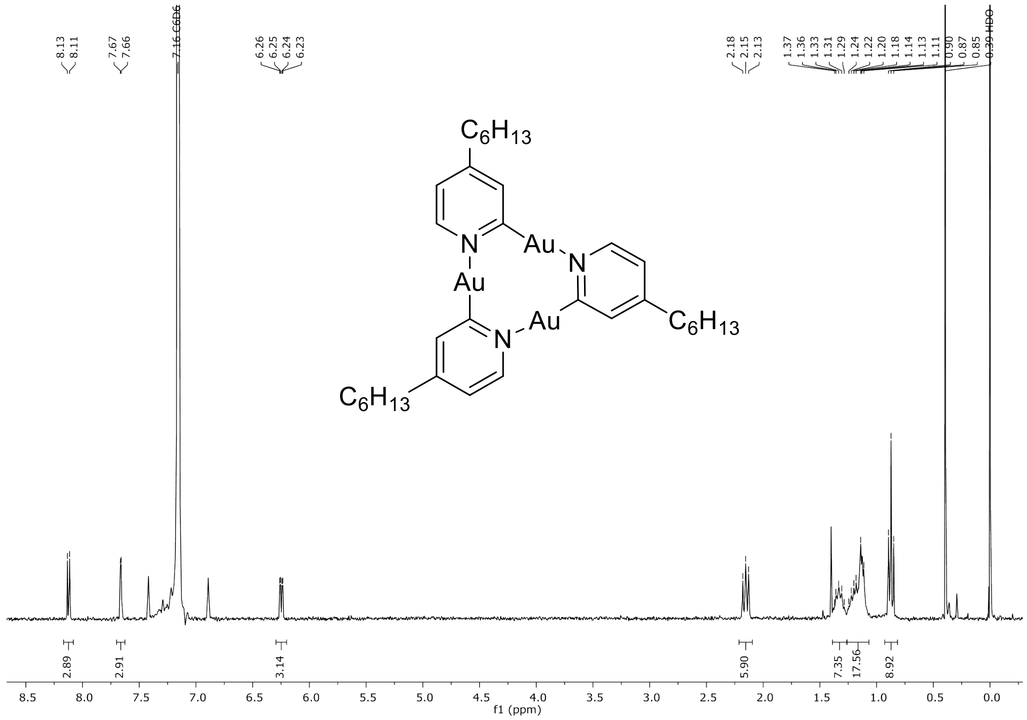

Figure S 43. ^{1}H NMR spectrum of 1b in $CDCl_3$ (400 MHz).

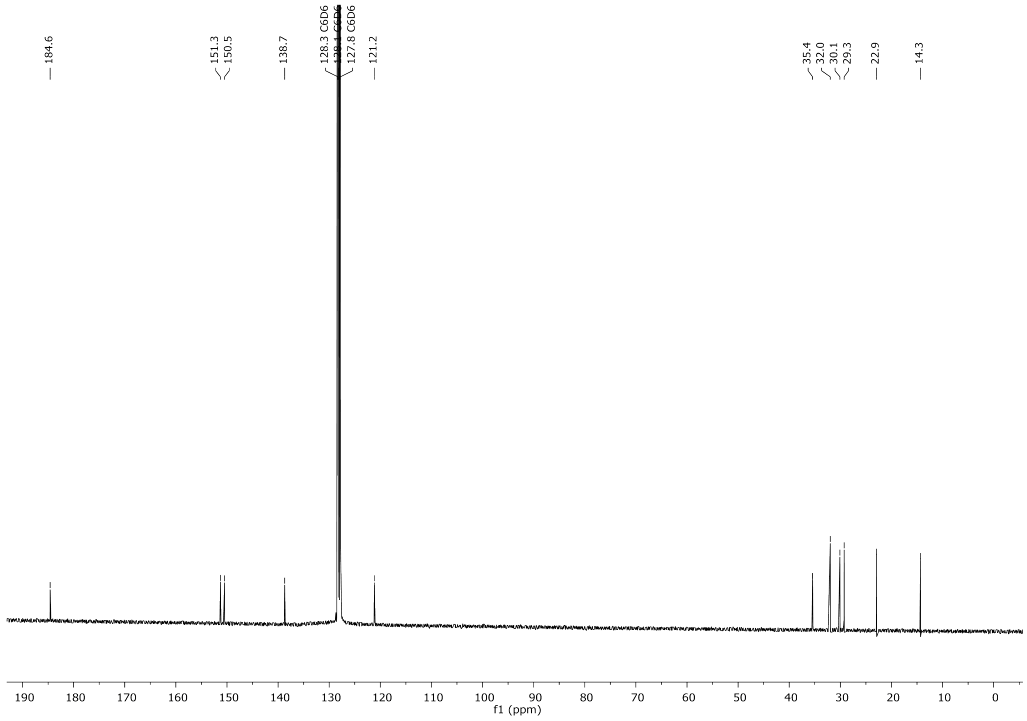

Figure S 44. ^{13}C NMR spectrum of 1b in $CDCl_3$ (101 MHz).

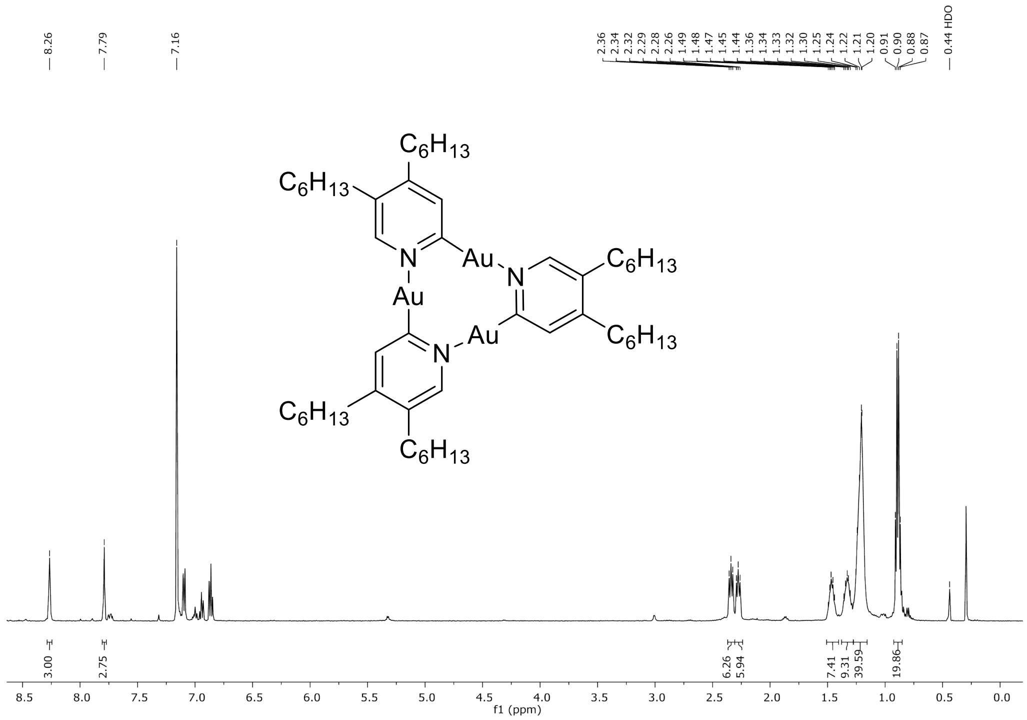

Figure S 45. ^{1}H NMR spectrum of 1c in $CDCl_3$ (500 MHz).

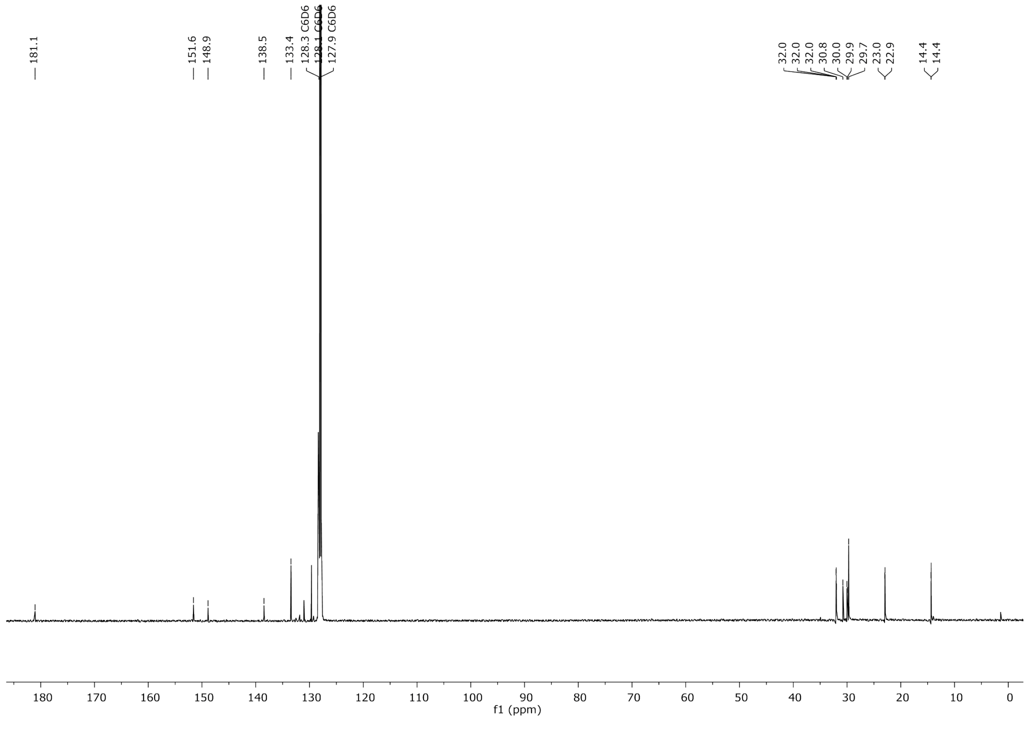

Figure S 46. ^{13}C NMR spectrum of 1c in $CDCl_3$ (126 MHz).

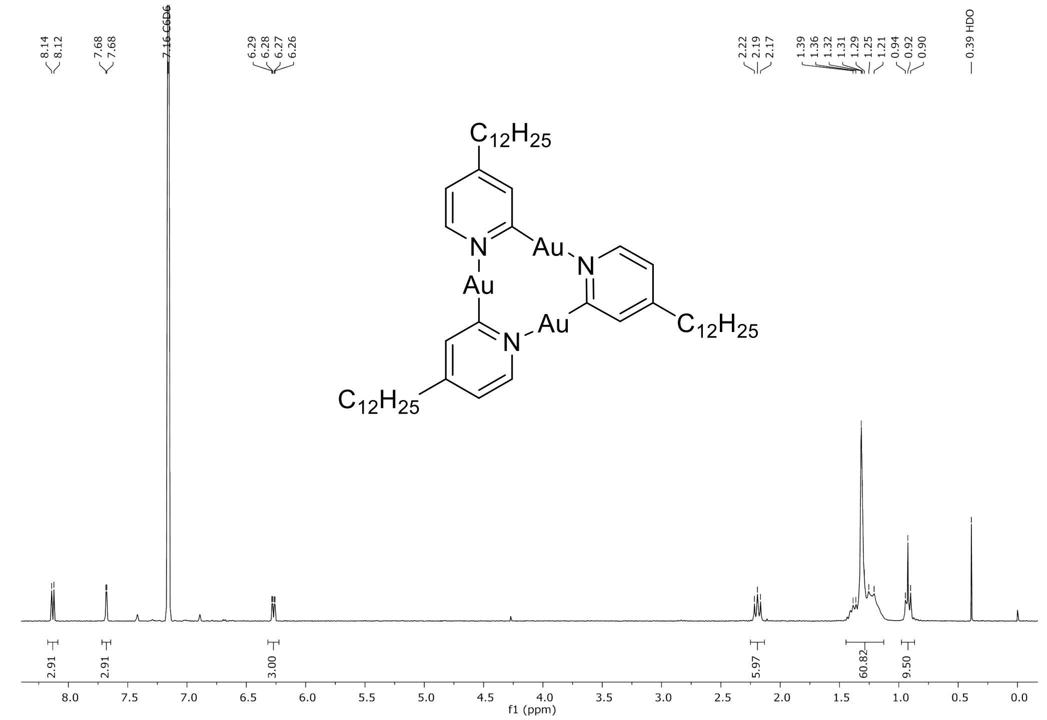

Figure S 47. ^{1}H NMR spectrum of 1d in $CDCl_3$ (400 MHz).

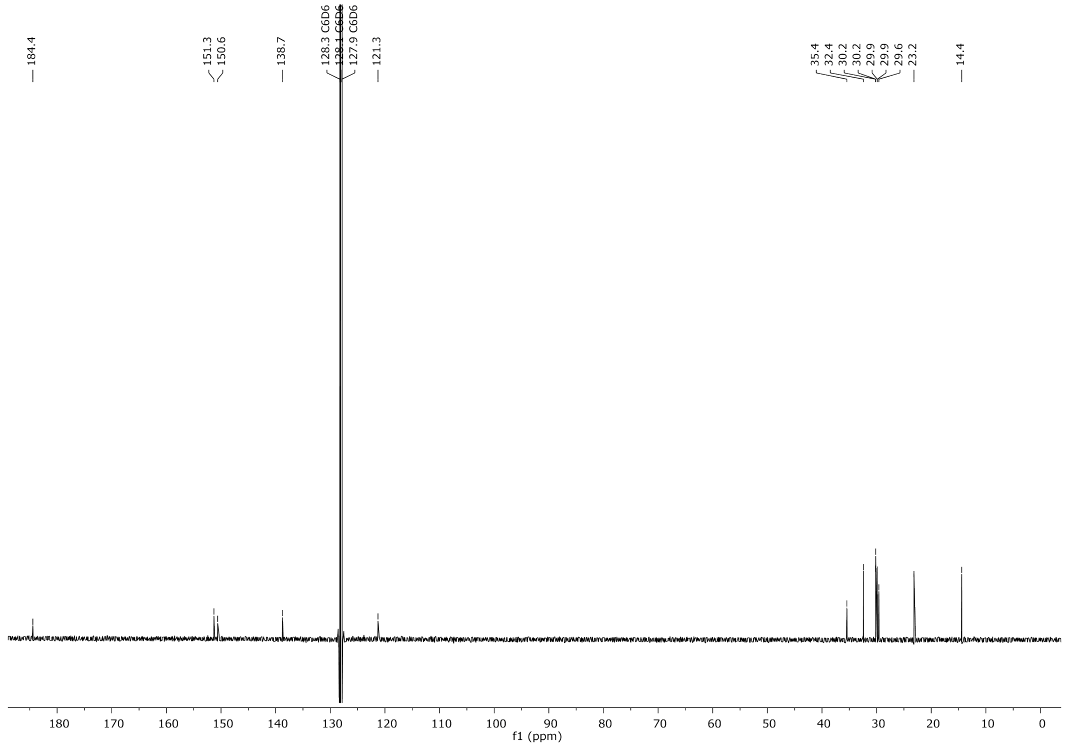

Figure S 48. ^{13}C NMR spectrum of 1d in $CDCl_3$ (101 MHz).

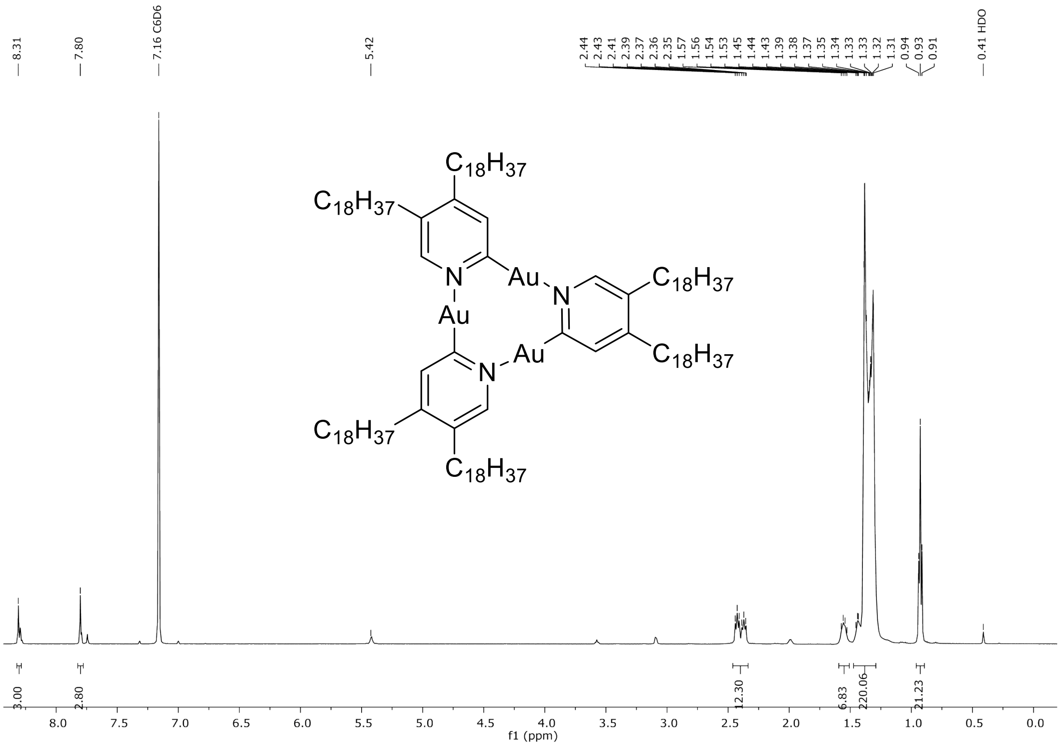

Figure S 49. ^{1}H NMR spectrum of 1e in $CDCl_3$ (500 MHz) at 313 K.

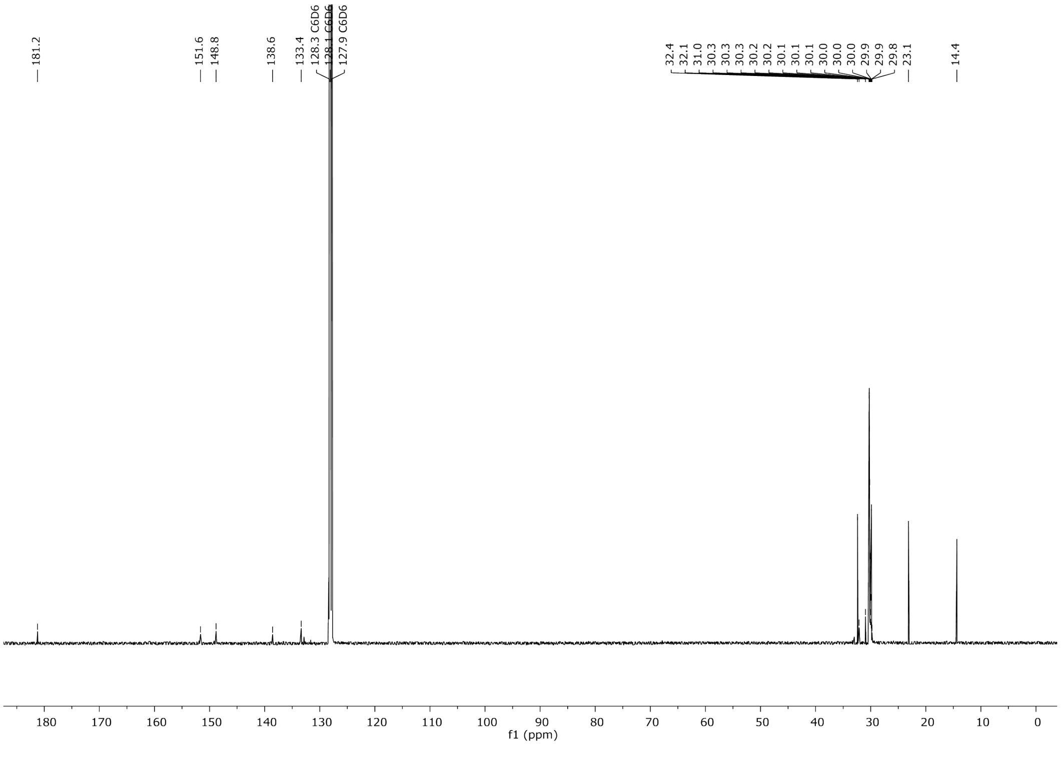

Figure S 50. ^{13}C NMR spectrum of 1e in $CDCl_3$ (126 MHz) at 313 K.

6 UV/Vis spectroscopy

Solutions of donor pyridinate **1d**, acceptor pyrazolates **2** and equal mixtures of both, donor and acceptor, were recorded by UV/Vis spectroscopy in *n*-hexanes, with concentrations between 1.0 x 10^{-6} and 1.0 x 10^{-4} mol/L. All spectra were measured at ten different concentrations, each was normalized to ε_λ, using the absorbance E_λ and the concentration *c*. The solutions containing both the donor and the acceptor in equal concentrations were normalized to the concentration of pyridinate **1d**.

$$\varepsilon_\lambda = \frac{E_\lambda}{c}$$

The analyzed samples were colorless, and no absorbance above λ = 400 nm was visible. A Charge-Transfer (CT) can be observed at λ = 250–350 nm.

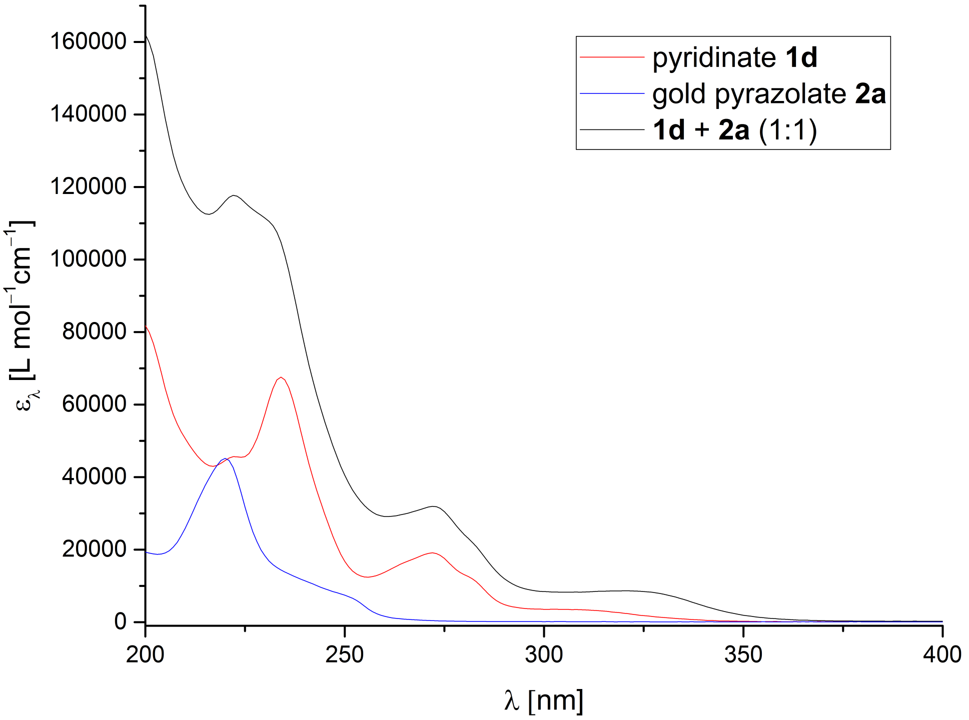

Figure S 51: UV/Vis spectra of pyridinate 1d (red), gold pyrazolate 2a (blue) and the 1:1 mixture of donor and acceptor (black).

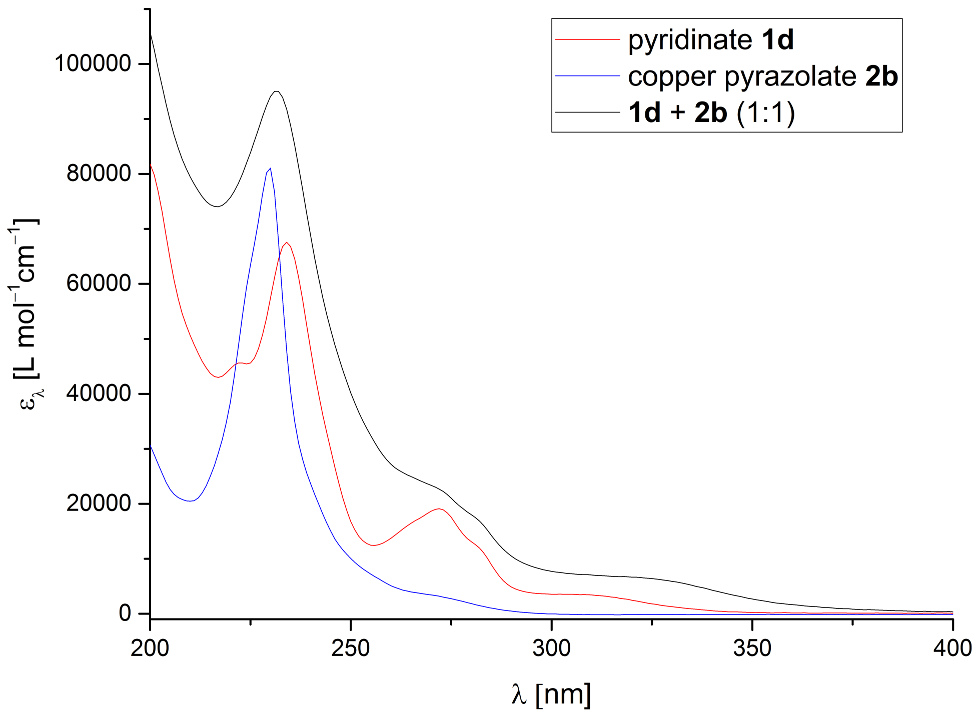

Figure S 52 UV/Vis spectra of pyridinate 1d (red), copper pyrazolate 2b (blue) and the 1:1 mixture of donor and acceptor (black).

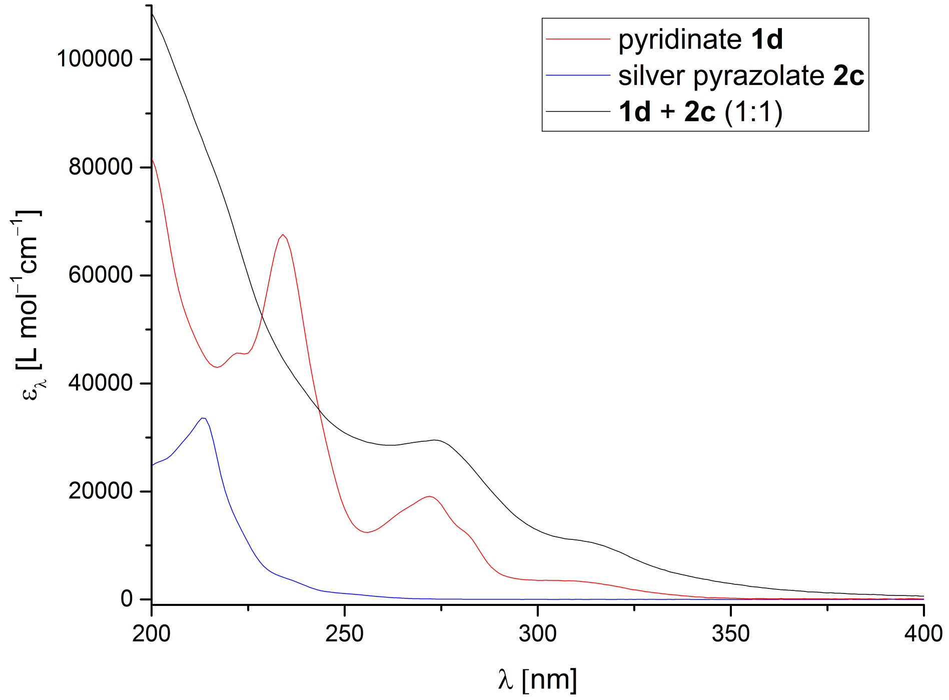

Figure S 53 UV/Vis spectra of pyridinate 1d (red), silver pyrazolate 2c (blue) and the 1:1 mixture of donor and acceptor (black).

7 Computational studies

7.1 General remarks

Our standard procedure for obtaining free energies ΔG is subdivided into three steps.[12] In step one, the electronic association energy E_a is computed from equilibrium structures in gas phase. All molecules are optimized in gas phase employing the density functional PBEh-3c.[13] This composite method is designed to yield accurate molecular geometries and makes use of a modified double-zeta (DZ) basis set[14] (dubbed def2-mSVP) balanced with the D3-dispersion[15] correction, including the three body contributions (D3^{ATM}) and a geometrical counter-poise correction (gCP).[16] Single-point energies are calculated employing the hybrid functional PBE0[17,18]-D3^{ATM} combined with the quadruple-zeta basis set (QZ). At this QZ basis set level the basis set superposition errors are typically in the range of less than 2% of ΔE_a and can therefore be discarded.[19] To account for physically correct description of the long range correlation (mainly London dispersion) the pairwise additive D3 dispersion correction with Becke-Johnson damping[20,21] is used for all single-point calculations. The Axilrod-Teller-Muto type three-body[22,23] dispersion energy is always included and many-body dispersion contributions beyond the three body term are neglected. The electronic energy comprises of

$$E = E_{el}^{DFT} + E_{disp}^{2} + E_{disp}^{3}$$

where E is the electronic energy of one species involved. The association energy ΔE_a in gas phase is calculated in the supramolecular approach

$$\Delta E_a = E_{product} - (E_{reactant1} + E_{reactant2})$$

where E denotes the total electronic energy of the species. In step two, the thermo-statistical corrections from energy to free energy $G_{\mathrm{RRHO}}^{\mathrm{T}}$ are calculated for each molecule.[12] The thermo-statistical corrections are obtained in gas phase at a given temperature T and normal pressure of 1 atm, including the zero-point vibrational energy. The computation of harmonic frequencies is carried out with the global hybrid PBEh-3c and for the larger systems with our newly developed tight binding method GFN-xTB.[24] The third step is the calculation of the solvation free energies $\delta G_{\mathrm{solv}}^{\mathrm{T}}(\mathrm{X})$ for each molecule at a given temperature T and a solvent X. To get the solvation free energies COSMO-RS[25–27] is used in a black boxed manner and all results are implicitly converted to standard state conditions. The final free energy ΔG_a is the sum of the three contributions:

$$\Delta G_a = \Delta E_a + \Delta G_{RRHO}^{T} + \Delta\delta G_{solv}^{T}(X)$$

7.2 Technical details

All DFT calculations were performed using the TURBOMOLE 7.0.2[28,29] program package. The resolution-of-identity (RI) approximation for the Coulomb integrals was generally applied[30] using matching default auxiliary basis sets.[31] For the integration of the exchange-correlation contribution the numerical quadrature grids m3 in case of PBEh-3c and m4 in case of PBE0-D3^{ATM} and PW6B95[32]-D3^{ATM} were employed. Single-point energies are calculated with PBE0-D3^{ATM}/(def2-QZ-f,-g) and PW6B95-D3^{ATM} /def2-QZVP. The default convergence criteria for single-point energies as well as for geometry optimizations were used [10^7 E_h for energies and 10^5 E_h/Bohr for gradients]. The vibrational frequencies of PBEh-3c were scaled with a factor of 0.95.[13] The conductor like screening model for real solvents (COSMO-RS) was used as implemented in COSMOtherm2016 employing the BP_TZVP_C30_1401-parametrization. For each solvation free energy value two single-point calculations with BP86[33,34]/def-TZVP (one in the gas-phase and one in an ideal conductor) have to be performed on the optimized structures. These calculations are then used as input for the COSMOtherm program. The solvent for the calculations was benzene and the temperature was set to 298.15 K. All visualizations were done with USCF Chimera[35] version 1.10.2.

The DLPNO-CCSD(T)[36,37] reference was calculated with the ORCA 4.0.0[38,39] implementation, i.e. employing the sparse maps infrastructure "TightPNO" setup (full LMP2 guess used, TCutPairs 1e-5 E_h, TCutPNO 1e-7, TCutDO 5e-3) and ORCA "TightSCF". The employed basis sets were: def2-TZVPP[14], def2-TZVPP/C[40] with corresponding ECPs for Ag and Au [Def2-ECP][41]; def2-QZVPP[14], def2-QZVPP/C[40] with corresponding ECPs for Ag and Au [Def2-ECP][41]. The ECP parameters for Au [Def2-ECP], Ag [Def2-ECP] have been obtained from TURBOMOLE 7.0.2. The CBS extrapolation has been performed with optimized exponents as proposed by Neese and Valeev.[42] The electronic association energy ΔE_a includes the deformation energy as well (6.8 kcal mol^{-1}, mostly from the Ag complex (**2c**)), estimated error of the final ΔE_a= +/− 0.5 kcal mol^{-1}.

Table S 12. DLPNO-CCSD(T)/CBS(TZ/QZ) values for 1f•2c (all energies in kcal mol^{-1}).

**1f** (1:1) with Acceptor **2c**	ΔE_{int}/CBS(TZ/QZ)	ΔE_{deform}/CBS(TZ/QZ)	ΔE_a (**1f•2c**) /CBS(TZ/QZ)
**2c** (M = Ag)	−45.9	6.8	−39.15

Table S 13. D-A Free binding energies calculated with PW6B95-D3/QZ (all energies in kcal mol^{-1}).

**1f** (1:1) with Acceptor **2**	Δ*E*(PW6B95-D3/QZ)	ΔG_{RRHO} (PBEh-3c)	$\Delta\delta G_{solv}$	ΔG_a (**1f 2**)
**2a** (M = Au)	−35.1	17.4	4.6	−13.2
**2b** (M = Cu)	−42.5	17.5	6.1	18.9
**2c** (M = Ag)	−43.7	17.2	6.0	−20.5

Table S 14: Dispersion-corrected interaction energies (all energies in kcal mol^{-1}).

**1f** (1:1) with Acceptor **2**	Δ*E*(D3(BJ)^{ATM}) (PBE0)	ΔE(DFT-D4 v 0.1)(PBE0) [a]	Δ*E*(PBE0/QZ-f-g)	Δ*E*(PBE0-D3/QZ-f-g)	ΔE(PBE0-(D4v0.1)/QZ-f-g)
**2a** (M = Au)	−31.8	−35.5	−0.3	−32.1	−35.8
**2b** (M = Cu)	−31.2	−34.9	−6.8	−38.0	−41.7
**2c** (M = Ag)	−31.3	−34.6	−8.3	−39.5	−42.9

[a] D4 v0.1 is a preliminary DFTD4 in-house development version that takes into account the influence of the electronic structure on the dynamic polarizability and thus the dispersion energy.[43]

Table S 15 Atomic C6(AA) coefficients of the metal centers calculated using D3 and D4.

**Molecule**	C6(AA)/(au · bohr^6) D3(PBE0)[ATM, BJ]	C6(AA)/(au · bohr^6) D4(PBE0)
**1f** (M = Au)	317.2	306.3
**2a** (M = Au)	317.2	303.7
**2b** (M = Cu)	175.0	160.6
**2c** (M = Ag)	268.6	244.6

Table S 16. D-A Free binding energies calculated with PBE0-D3/QZ and thermostatistical contributions obtained with GFN-xTB (all energies in kcal mol^{-1}).

**1f** (1:1) with Acceptor **2**	ΔE(PBE0-D3/QZ-f-g)	ΔG_{RRHO} (GFN-xTB)	$\Delta\delta G_{solv}$	ΔG_a (**1f•2**)
**2a** (M = Au)	−32.1	22.3	4.6	−5.2
**2b** (M = Cu)	−38.0	Could not be obtained	6.1	–
**2c** (M = Ag)	−39.5	18.4	6.0	−15.2

Table S 17. A-D-A Free binding energies calculated with PBE0-D3/QZ and thermostatistical contributions obtained with GFN-xTB (all energies in kcal mol^{-1}).

**1f • 2** with Acceptor to **2•1f•2**	ΔE(PBE0-D3/QZ-f-g)	ΔG_{RRHO} (GFN-xTB)	$\Delta\delta G_{solv}$	ΔG_a (**2•1f•2**)
**2a** (M = Au)	−30.3	14.1	4.9	−11.4
**2b** (M = Cu)	−35.1	Could not be obtained	5.9	–
**2c** (M = Ag)	−36.4	11.0	4.9	−20.6

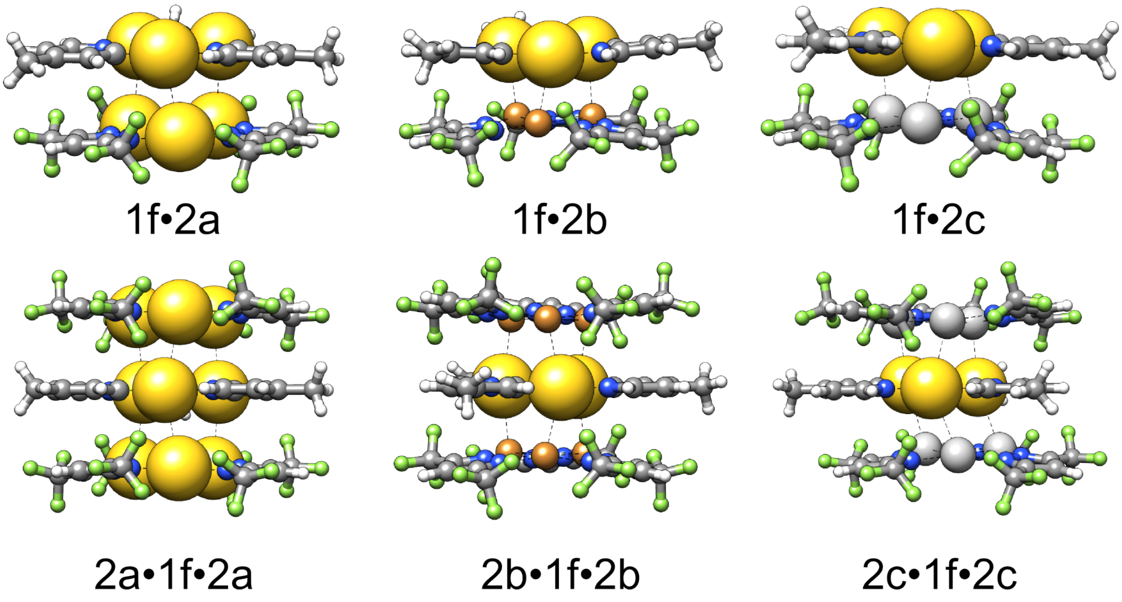

Figure S 54. Structures of stacked CTCs.

7.2.1 Qualitative evaluation of Charge-Transfer from the gold pyridinate complex 1f on a molecular extended graphite surface

To obtain a qualitative statement about the possible CT from **1f** onto the HOPG, the complex **1f** on a molecular extended graphite surface was optimized with PBE-D3/def2-SVP.[44] The CT was then calculated from the Natural Population Analysis, as well as from the total density and produced the same conclusion that only a charge of −0.00988 is transferred onto the molecular graphene surface. In general CT interactions are 'overestimated with GGAs' therefore we qualitatively concluded that for the donor **1f** CT is negligible small.

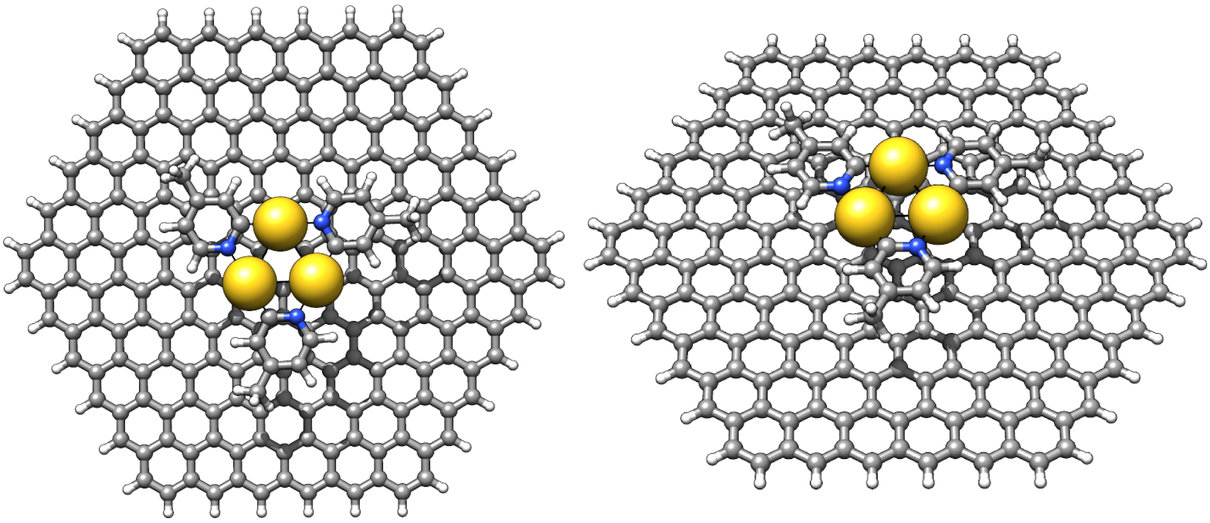

Figure S 55: CTC 1f on molecular graphite surface.

7.2.2 Association of 1f • 2a on a molecular extended graphite surface

The association of **1f** with **2a** on a molecular extended graphite surface in the gas phase was investigated. The structures were optimized with the global hybrid composite scheme PBEh-3c, and thermostatistical contributions were obtained with GFN-xTB.

For the complex on the graphite surface no charge transfer was found. A small dipole moment (0.13 debye) orthogonal to the graphite surface was found (for **1f** on the surface), which indicates only a (very) small polarization.

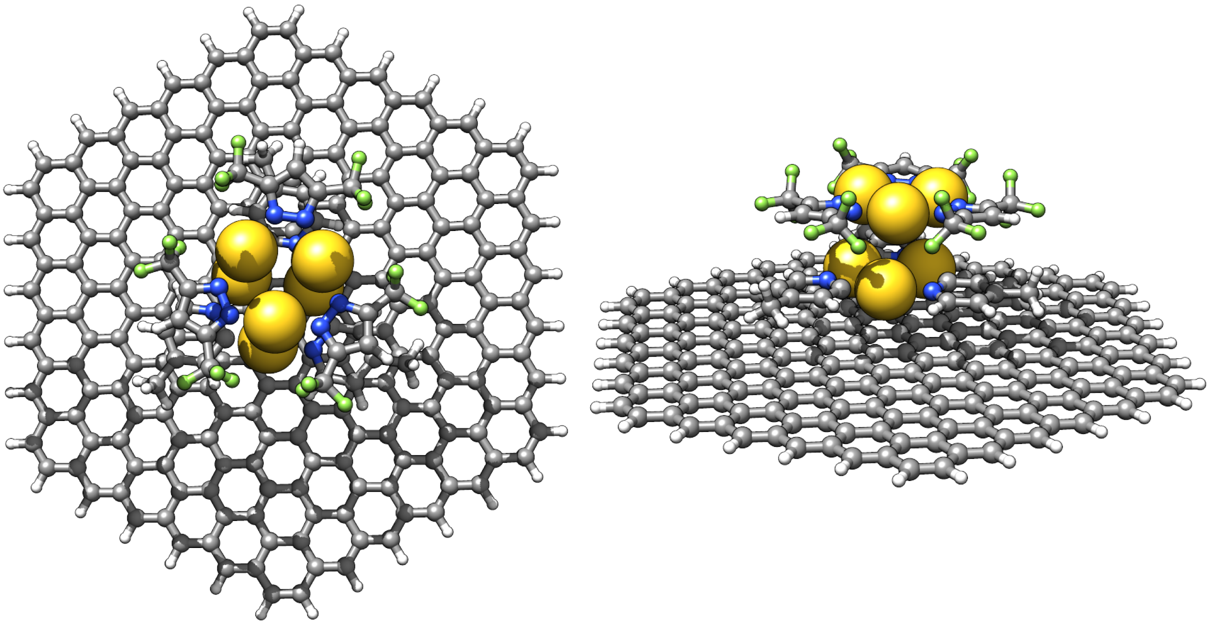

Figure S 56: Complex 1f • 2a on molecular graphite surface.

Listed below are the Cartesian coordinates of the optimized structures at the level of PBEh-3c.

1f-on molecular graphene surface:

294

C 11.3445368 -7.3819521 0.3530914

C 12.0545368 -6.1521921 0.3530914

C 11.3445368 -4.9224321 0.3530914

C 12.0545368 -3.6926821 0.3530914

C 11.3445368 -2.4629221 0.3530914

C 12.0545368 -1.2331721 0.3530914

C 11.3445368 -0.0034121 0.3530914

C 12.0545368 1.2263479 0.3530914

C 11.3445368 2.4560979 0.3530914

C 12.0545368 3.6858579 0.3530914

C 11.3445368 4.9156179 0.3530914

C 12.0545368 6.1453679 0.3530914

C 11.3445368 7.3751279 0.3530914

C 9.2145368 -8.6117021 0.3530914

C 9.9245368 -7.3819521 0.3530914

C 9.2145368 -6.1521921 0.3530914

C 9.9245368 -4.9224321 0.3530914

C 9.2145368 -3.6926821 0.3530914

C 9.9245368 -2.4629221 0.3530914

C 9.2145368 -1.2331721 0.3530914

C 9.9245368 -0.0034121 0.3530914

C 9.2145368 1.2263479 0.3530914

C 9.9245368 2.4560979 0.3530914

C 9.2145368 3.6858579 0.3530914

C 9.9245368 4.9156179 0.3530914

C 9.2145368 6.1453679 0.3530914

C 9.9245368 7.3751279 0.3530914

C 9.2145368 8.6048779 0.3530914

C 7.0845368 -9.8414621 0.3530914

C 7.7945368 -8.6117021 0.3530914

C 7.0845368 -7.3819521 0.3530914

C 7.7945368 -6.1521921 0.3530914

C 7.0845368 -4.9224321 0.3530914

C 7.7945368 -3.6926821 0.3530914

C 7.0845368 -2.4629221 0.3530914

C 7.7945368 -1.2331721 0.3530914

C 7.0845368 -0.0034121 0.3530914

C 7.7945368 1.2263479 0.3530914

C 7.0845368 2.4560979 0.3530914

C 7.7945368 3.6858579 0.3530914

C 7.0845368 4.9156179 0.3530914

C 7.7945368 6.1453679 0.3530914

C 7.0845368 7.3751279 0.3530914

C 7.7945368 8.6048779 0.3530914

C 7.0845368 9.8346379 0.3530914

C 4.9545368 -11.0712121 0.3530914

C 5.6645368 -9.8414621 0.3530914

C 4.9545368 -8.6117021 0.3530914

C 5.6645368 -7.3819521 0.3530914

C 4.9545368 -6.1521921 0.3530914

C 5.6645368 -4.9224321 0.3530914

C 4.9545368 -3.6926821 0.3530914

C 5.6645368 -2.4629221 0.3530914

C 4.9545368 -1.2331721 0.3530914

C 5.6645368 -0.0034121 0.3530914

C 4.9545368 1.2263479 0.3530914

C 5.6645368 2.4560979 0.3530914

C 4.9545368 3.6858579 0.3530914

C 5.6645368 4.9156179 0.3530914

C	4.9545368	6.1453679	0.3530914
C	5.6645368	7.3751279	0.3530914
C	4.9545368	8.6048779	0.3530914
C	5.6645368	9.8346379	0.3530914
C	4.9545368	11.0643979	0.3530914
C	2.8245368	-12.3009721	0.3530914
C	3.5345368	-11.0712121	0.3530914
C	2.8245368	-9.8414621	0.3530914
C	3.5345368	-8.6117021	0.3530914
C	2.8245368	-7.3819521	0.3530914
C	3.5345368	-6.1521921	0.3530914
C	2.8245368	-4.9224321	0.3530914
C	3.5345368	-3.6926821	0.3530914
C	2.8245368	-2.4629221	0.3530914
C	3.5345368	-1.2331721	0.3530914
C	2.8245368	-0.0034121	0.3530914
C	3.5345368	1.2263479	0.3530914
C	2.8245368	2.4560979	0.3530914
C	3.5345368	3.6858579	0.3530914
C	2.8245368	4.9156179	0.3530914
C	3.5345368	6.1453679	0.3530914
C	2.8245368	7.3751279	0.3530914
C	3.5345368	8.6048779	0.3530914
C	2.8245368	9.8346379	0.3530914
C	3.5345368	11.0643979	0.3530914
C	2.8245368	12.2941479	0.3530914
C	0.6945368	-13.5307321	0.3530914
C	1.4045368	-12.3009721	0.3530914
C	0.6945368	-11.0712121	0.3530914
C	1.4045368	-9.8414621	0.3530914
C	0.6945368	-8.6117021	0.3530914
C	1.4045368	-7.3819521	0.3530914
C	0.6945368	-6.1521921	0.3530914
C	1.4045368	-4.9224321	0.3530914
C	0.6945368	-3.6926821	0.3530914
C	1.4045368	-2.4629221	0.3530914
C	0.6945368	-1.2331721	0.3530914
C	1.4045368	-0.0034121	0.3530914
C	0.6945368	1.2263479	0.3530914
C	1.4045368	2.4560979	0.3530914
C	0.6945368	3.6858579	0.3530914
C	1.4045368	4.9156179	0.3530914
C	0.6945368	6.1453679	0.3530914
C	1.4045368	7.3751279	0.3530914
C	0.6945368	8.6048779	0.3530914
C	1.4045368	9.8346379	0.3530914
C	0.6945368	11.0643979	0.3530914
C	1.4045368	12.2941479	0.3530914
C	0.6945368	13.5239079	0.3530914
C	-0.7254632	-13.5307321	0.3530914
C	-1.4354632	-12.3009721	0.3530914
C	-0.7254632	-11.0712121	0.3530914
C	-1.4354632	-9.8414621	0.3530914
C	-0.7254632	-8.6117021	0.3530914
C	-1.4354632	-7.3819521	0.3530914
C	-0.7254632	-6.1521921	0.3530914
C	-1.4354632	-4.9224321	0.3530914
C	-0.7254632	-3.6926821	0.3530914
C	-1.4354632	-2.4629221	0.3530914
C	-0.7254632	-1.2331721	0.3530914
C	-1.4354632	-0.0034121	0.3530914
C	-0.7254632	1.2263479	0.3530914

C	-1.4354632	2.4560979	0.3530914
C	-0.7254632	3.6858579	0.3530914
C	-1.4354632	4.9156179	0.3530914
C	-0.7254632	6.1453679	0.3530914
C	-1.4354632	7.3751279	0.3530914
C	-0.7254632	8.6048779	0.3530914
C	-1.4354632	9.8346379	0.3530914
C	-0.7254632	11.0643979	0.3530914
C	-1.4354632	12.2941479	0.3530914
C	-0.7254632	13.5239079	0.3530914
C	-2.8554632	-12.3009721	0.3530914
C	-3.5654632	-11.0712121	0.3530914
C	-2.8554632	-9.8414621	0.3530914
C	-3.5654632	-8.6117021	0.3530914
C	-2.8554632	-7.3819521	0.3530914
C	-3.5654632	-6.1521921	0.3530914
C	-2.8554632	-4.9224321	0.3530914
C	-3.5654632	-3.6926821	0.3530914
C	-2.8554632	-2.4629221	0.3530914
C	-3.5654632	-1.2331721	0.3530914
C	-2.8554632	-0.0034121	0.3530914
C	-3.5654632	1.2263479	0.3530914
C	-2.8554632	2.4560979	0.3530914
C	-3.5654632	3.6858579	0.3530914
C	-2.8554632	4.9156179	0.3530914
C	-3.5654632	6.1453679	0.3530914
C	-2.8554632	7.3751279	0.3530914
C	-3.5654632	8.6048779	0.3530914
C	-2.8554632	9.8346379	0.3530914
C	-3.5654632	11.0643979	0.3530914
C	-2.8554632	12.2941479	0.3530914
C	-4.9854632	-11.0712121	0.3530914
C	-5.6954632	-9.8414621	0.3530914
C	-4.9854632	-8.6117021	0.3530914
C	-5.6954632	-7.3819521	0.3530914
C	-4.9854632	-6.1521921	0.3530914
C	-5.6954632	-4.9224321	0.3530914
C	-4.9854632	-3.6926821	0.3530914
C	-5.6954632	-2.4629221	0.3530914
C	-4.9854632	-1.2331721	0.3530914
C	-5.6954632	-0.0034121	0.3530914
C	-4.9854632	1.2263479	0.3530914
C	-5.6954632	2.4560979	0.3530914
C	-4.9854632	3.6858579	0.3530914
C	-5.6954632	4.9156179	0.3530914
C	-4.9854632	6.1453679	0.3530914
C	-5.6954632	7.3751279	0.3530914
C	-4.9854632	8.6048779	0.3530914
C	-5.6954632	9.8346379	0.3530914
C	-4.9854632	11.0643979	0.3530914
C	-7.1154632	-9.8414621	0.3530914
C	-7.8254632	-8.6117021	0.3530914
C	-7.1154632	-7.3819521	0.3530914
C	-7.8254632	-6.1521921	0.3530914
C	-7.1154632	-4.9224321	0.3530914
C	-7.8254632	-3.6926821	0.3530914
C	-7.1154632	-2.4629221	0.3530914
C	-7.8254632	-1.2331721	0.3530914
C	-7.1154632	-0.0034121	0.3530914
C	-7.8254632	1.2263479	0.3530914
C	-7.1154632	2.4560979	0.3530914
C	-7.8254632	3.6858579	0.3530914

C	-7.1154632	4.9156179	0.3530914
C	-7.8254632	6.1453679	0.3530914
C	-7.1154632	7.3751279	0.3530914
C	-7.8254632	8.6048779	0.3530914
C	-7.1154632	9.8346379	0.3530914
C	-9.2454632	-8.6117021	0.3530914
C	-9.9554632	-7.3819521	0.3530914
C	-9.2454632	-6.1521921	0.3530914
C	-9.9554632	-4.9224321	0.3530914
C	-9.2454632	-3.6926821	0.3530914
C	-9.9554632	-2.4629221	0.3530914
C	-9.2454632	-1.2331721	0.3530914
C	-9.9554632	-0.0034121	0.3530914
C	-9.2454632	1.2263479	0.3530914
C	-9.9554632	2.4560979	0.3530914
C	-9.2454632	3.6858579	0.3530914
C	-9.9554632	4.9156179	0.3530914
C	-9.2454632	6.1453679	0.3530914
C	-9.9554632	7.3751279	0.3530914
C	-9.2454632	8.6048779	0.3530914
C	-11.3754632	-7.3819521	0.3530914
C	-12.0854632	-6.1521921	0.3530914
C	-11.3754632	-4.9224321	0.3530914
C	-12.0854632	-3.6926821	0.3530914
C	-11.3754632	-2.4629221	0.3530914
C	-12.0854632	-1.2331721	0.3530914
C	-11.3754632	-0.0034121	0.3530914
C	-12.0854632	1.2263479	0.3530914
C	-11.3754632	2.4560979	0.3530914
C	-12.0854632	3.6858579	0.3530914
C	-11.3754632	4.9156179	0.3530914

C	-12.0854632	6.1453679	0.3530914
C	-11.3754632	7.3751279	0.3530914
H	1.2105368	14.4176479	0.3530914
H	-1.2414632	14.4176479	0.3530914
H	3.3405368	13.1878879	0.3530914
H	5.4705368	11.9581379	0.3530914
H	7.6005368	10.7283779	0.3530914
H	9.7305368	9.4986179	0.3530914
H	11.8605368	8.2688679	0.3530914
H	13.0865368	6.1453679	0.3530914
H	13.0865368	3.6858579	0.3530914
H	13.0865368	1.2263479	0.3530914
H	13.0865368	-1.2331721	0.3530914
H	13.0865368	-3.6926821	0.3530914
H	13.0865368	-6.1521921	0.3530914
H	11.8605368	-8.2756921	0.3530914
H	9.7305368	-9.5054421	0.3530914
H	5.4705368	-11.9649521	0.3530914
H	3.3405368	-13.1947121	0.3530914
H	1.2105368	-14.4244721	0.3530914
H	-1.2414632	-14.4244721	0.3530914
H	-3.3714632	-13.1947121	0.3530914
H	-5.5014632	-11.9649521	0.3530914
H	-9.7614632	-9.5054421	0.3530914
H	-11.8914632	-8.2756921	0.3530914
H	-13.1174632	-6.1521921	0.3530914
H	-13.1174632	-3.6926821	0.3530914
H	-13.1174632	-1.2331721	0.3530914
H	-13.1174632	1.2263479	0.3530914
H	-13.1174632	6.1453679	0.3530914
H	-9.7614632	9.4986179	0.3530914

H -5.5014632 11.9581379 0.3530914

H -3.3714632 13.1878879 0.3530914

H 7.6005368 -10.7352021 0.3530914

H -7.6314632 -10.7352021 0.3530914

H -13.1174632 3.6858579 0.3530914

H -7.6314632 10.7283779 0.3530914

H -11.8914632 8.2688679 0.3530914

C 2.9041132 0.6867667 -3.0545400

N 2.8932979 -0.6859260 -3.0537656

C 4.0563254 -1.3825242 -3.0351572

C 5.3037454 -0.7649096 -3.0275880

C 5.3743296 0.6443214 -3.0398941

C 4.1565394 1.3443354 -3.0469686

Au 1.0415473 -1.6997792 -3.0517293

Au 1.1655791 1.6824785 -3.0440013

Au -1.8324861 0.0997593 -3.0414537

N -1.8752782 -2.0123046 -3.0429019

C -0.6910320 -2.7062744 -3.0541133

C -0.7451984 -4.1201161 -3.0471685

C -1.9585695 -4.8271514 -3.0285292

C -3.1448761 -4.0629014 -3.0042386

C -3.0585758 -2.6737131 -3.0130700

C -1.9991452 -6.3306111 -3.0511050

C 6.6964119 1.3611274 -3.0644717

C -1.8355068 2.1037920 -3.0451768

N -0.6397657 2.7781518 -3.0407338

C -0.6148694 4.1335673 -3.0167515

C -1.7713603 4.9080199 -3.0080239

C -3.0287993 4.2675137 -3.0238211

C -3.0294851 2.8628378 -3.0349552

C -4.3079725 5.0584603 -3.0479687

H 0.3838336 4.5958208 -2.9933156

H -1.6925992 6.0048940 -2.9743437

H -3.9881466 2.3222690 -3.0251593

H 0.2044582 -4.6762507 -3.0445681

H -4.1319741 -4.5469254 -2.9641504

H -3.9611725 -2.0440360 -2.9850825

H 3.9597262 -2.4790644 -3.0154817

H 6.2156059 -1.3796790 -2.9970955

H 4.1649304 2.4447958 -3.0344839

H -5.1540643 4.4818387 -2.6270548

H -4.2130218 6.0048772 -2.4808425

H -4.5754036 5.3270437 -4.0924796

H 6.6220078 2.3707432 -2.6167271

H 7.4783519 0.7949402 -2.5221366

H 7.0462698 1.4870143 -4.1115011

H -1.0530952 -6.7709288 -2.6821557

H -2.8318111 -6.7241617 -2.4356333

H -2.1576481 -6.6965510 -4.0882538

Donor 1f:

42

C 2.7586753 0.6409186 0.0000000

N 2.7489893 -0.7107667 0.0000000

C 3.8985805 -1.3980901 0.0000000

C 5.1311477 -0.7932038 0.0000000

C 5.1985168 0.5992906 0.0000000

C 3.9998576 1.2906833 0.0000000

Au 0.9115834 -1.7113960 0.0000000

Au	1.0263207	1.6451524	0.0000000
Au	-1.9379042	0.0662436	0.0000000
N	-1.9900367	-2.0253112	0.0000000
C	-0.8242859	-2.7095422	0.0000000
C	-0.8821642	-4.1093199	0.0000000
C	-2.0802575	-4.8016929	0.0000000
C	-3.2525085	-4.0471023	0.0000000
C	-3.1600718	-2.6772246	0.0000000
C	-2.1309206	-6.2980628	0.0000000
C	6.5197427	1.3036000	0.0000000
C	-1.9343895	2.0686236	0.0000000
N	-0.7589526	2.7360779	0.0000000
C	-0.7385087	4.0753148	0.0000000
C	-1.8786392	4.8403061	0.0000000
C	-3.1182593	4.2024023	0.0000000
C	-3.1176933	2.8186366	0.0000000
C	-4.3888221	4.9944628	0.0000000
H	0.2373497	4.5430022	0.0000000
H	-1.7996144	5.9194242	0.0000000
H	-4.0627029	2.2903475	0.0000000
H	0.0478524	-4.6635777	0.0000000
H	-4.2265645	-4.5182239	0.0000000
H	-4.0530302	-2.0659502	0.0000000
H	3.8156805	-2.4770520	0.0000000
H	6.0261790	-1.4012003	0.0000000
H	4.0148505	2.3732302	0.0000000
H	-5.2656489	4.3482243	0.0000000
H	-4.4481967	5.6387885	0.8770730
H	-4.4481967	5.6387885	-0.8770730
H	6.3984972	2.3860735	0.0000000
H	7.1074324	1.0328571	0.8770730
H	7.1074324	1.0328571	-0.8770730
H	-1.1328483	-6.7342978	0.0000000
H	-2.6592357	-6.6716456	0.8770730
H	-2.6592357	-6.6716456	-0.8770730

Acceptor 2a:

45

N	-2.7152836	0.6651895	0.0000000
N	-2.7152836	-0.6651895	0.0000000
C	-3.9802458	-1.0865168	0.0000000
C	-4.8346832	0.0000000	0.0000000
C	-3.9802458	1.0865168	0.0000000
Au	-0.9814476	-1.6999172	0.0000000
Au	-0.9814476	1.6999172	0.0000000
Au	1.9628953	0.0000000	0.0000000
N	1.9337128	-2.0189098	0.0000000
N	0.7815708	-2.6840993	0.0000000
C	1.0491718	-3.9902524	0.0000000
C	2.4173416	-4.1869584	0.0000000
C	2.9310740	-2.9037356	0.0000000
C	-0.0340473	-5.0132128	0.0000000
C	4.3585933	-2.4771206	0.0000000
C	-4.3245461	-2.5360922	0.0000000
C	-4.3245461	2.5360922	0.0000000
N	1.9337128	2.0189098	0.0000000
N	0.7815708	2.6840993	0.0000000
C	1.0491718	3.9902524	0.0000000

C 2.4173416 4.1869584 0.0000000

C 2.9310740 2.9037356 0.0000000

C 4.3585933 2.4771206 0.0000000

C -0.0340473 5.0132128 0.0000000

H -5.9103689 0.0000000 0.0000000

H 2.9551844 -5.1185296 0.0000000

H 2.9551844 5.1185296 0.0000000

F 5.1540013 3.5442169 0.0000000

F 4.6511144 1.7441197 1.0746789

F 4.6511144 1.7441197 -1.0746789

F 5.1540013 -3.5442169 0.0000000

F 4.6511144 -1.7441197 -1.0746789

F 4.6511144 -1.7441197 1.0746789

F 0.4923812 6.2356045 0.0000000

F -0.8151052 4.9000431 1.0746789

F -0.8151052 4.9000431 -1.0746789

F 0.4923812 -6.2356045 0.0000000

F -0.8151052 -4.9000431 -1.0746789

F -0.8151052 -4.9000431 1.0746789

F -5.6463826 -2.6913876 0.0000000

F -3.8360092 -3.1559234 -1.0746789

F -3.8360092 -3.1559234 1.0746789

F -5.6463826 2.6913876 0.0000000

F -3.8360092 3.1559234 1.0746789

F -3.8360092 3.1559234 -1.0746789

Acceptor 2b:

45

N -2.5682691 0.6656208 0.0000000

N -2.5682691 -0.6656208 0.0000000

C -3.8347611 -1.0828607 0.0000000

C -4.6944980 0.0000000 0.0000000

C -3.8347611 1.0828607 0.0000000

Cu -0.9513894 -1.6478549 0.0000000

Cu -0.9513894 1.6478549 0.0000000

Cu 1.9027789 0.0000000 0.0000000

N 1.8605791 -1.8913759 0.0000000

N 0.7076900 -2.5569967 0.0000000

C 0.9795956 -3.8624309 0.0000000

C 2.3472490 -4.0655545 0.0000000

C 2.8551654 -2.7795701 0.0000000

C -0.1142157 -4.8693444 0.0000000

C 4.2740838 -2.3357585 0.0000000

C -4.1598681 -2.5335859 0.0000000

C -4.1598681 2.5335859 0.0000000

N 1.8605791 1.8913759 0.0000000

N 0.7076900 2.5569967 0.0000000

C 0.9795956 3.8624309 0.0000000

C 2.3472490 4.0655545 0.0000000

C 2.8551654 2.7795701 0.0000000

C 4.2740838 2.3357585 0.0000000

C -0.1142157 4.8693444 0.0000000

H -5.7703048 0.0000000 0.0000000

H 2.8851524 -4.9972306 0.0000000

H 2.8851524 4.9972306 0.0000000

F 5.0936082 3.3834412 0.0000000

F 4.5535037 1.5915435 1.0734898

F 4.5535037 1.5915435 -1.0734898

F 5.0936082 -3.3834412 0.0000000

F 4.5535037 -1.5915435 -1.0734898
F 4.5535037 -1.5915435 1.0734898
F 0.3833419 6.1029147 0.0000000
F -0.8984347 4.7392216 1.0734898
F -0.8984347 4.7392216 -1.0734898
F 0.3833419 -6.1029147 0.0000000
F -0.8984347 -4.7392216 -1.0734898
F -0.8984347 -4.7392216 1.0734898
F -5.4769501 -2.7194735 0.0000000
F -3.6550689 -3.1476781 -1.0734898
F -3.6550689 -3.1476781 1.0734898
F -5.4769501 2.7194735 0.0000000
F -3.6550689 3.1476781 1.0734898
F -3.6550689 3.1476781 -1.0734898

Acceptor 2c:

45

N -2.8038123 0.6641871 0.0000000
N -2.8038123 -0.6641871 0.0000000
C -4.0693438 -1.0826358 0.0000000
C -4.9301135 0.0000000 0.0000000
C -4.0693438 1.0826358 0.0000000
Ag -1.0257863 -1.7767139 0.0000000
Ag -1.0257863 1.7767139 0.0000000
Ag 2.0515725 0.0000000 0.0000000
N 1.9771090 -2.0960792 0.0000000
N 0.8267033 -2.7602662 0.0000000
C 1.0970818 -4.0654730 0.0000000
C 2.4650567 -4.2696035 0.0000000
C 2.9722620 -2.9828372 0.0000000
C 0.0004463 -5.0688853 0.0000000
C 4.3895603 -2.5348291 0.0000000
C -4.3900066 -2.5340561 0.0000000
C -4.3900066 2.5340561 0.0000000
N 1.9771090 2.0960792 0.0000000
N 0.8267033 2.7602662 0.0000000
C 1.0970818 4.0654730 0.0000000
C 2.4650567 4.2696035 0.0000000
C 2.9722620 2.9828372 0.0000000
C 4.3895603 2.5348291 0.0000000
C 0.0004463 5.0688853 0.0000000
H -6.0060043 0.0000000 0.0000000
H 3.0030021 -5.2013523 0.0000000
H 3.0030021 5.2013523 0.0000000
F 5.2148084 3.5771491 0.0000000
F 4.6664923 1.7866542 1.0732117
F 4.6664923 1.7866542 -1.0732117
F 5.2148084 -3.5771491 0.0000000
F 4.6664923 -1.7866542 -1.0732117
F 4.6664923 -1.7866542 1.0732117
F 0.4904978 6.3047311 0.0000000
F -0.7859582 4.9346280 1.0732117
F -0.7859582 4.9346280 -1.0732117
F 0.4904978 -6.3047311 0.0000000
F -0.7859582 -4.9346280 -1.0732117
F -0.7859582 -4.9346280 1.0732117
F -5.7053062 -2.7275820 0.0000000
F -3.8805341 -3.1479738 -1.0732117
F -3.8805341 -3.1479738 1.0732117
F -5.7053062 2.7275820 0.0000000
F -3.8805341 3.1479738 1.0732117

F -3.8805341 3.1479738 -1.0732117

1f•2a:

87

C 2.8170249 0.2852113 1.7764889
N 2.6438309 -1.0556747 1.8143584
C 3.6998020 -1.8772437 1.8459387
C 4.9969720 -1.4261563 1.8177123
C 5.2312774 -0.0545824 1.7560543
C 4.1256526 0.7792313 1.7467380
Au 0.6986733 -1.8228315 1.7587588
Au 1.2167825 1.4908784 1.7938101
Au -1.9120528 0.2823386 1.7922935
N -2.2196542 -1.7863204 1.8163520
C -1.1455982 -2.6057654 1.7503917
C -1.3725242 -3.9852850 1.6969824
C -2.6473112 -4.5260850 1.7103941
C -3.7171225 -3.6388444 1.8013511
C -3.4587659 -2.2906821 1.8523523
C -2.8781132 -6.0023084 1.6262540
C 6.6258114 0.4852919 1.6985788
C -1.6680596 2.2710023 1.8141938
N -0.4204137 2.7907000 1.8647769
C -0.2370328 4.1152583 1.9203470
C -1.2761840 5.0134674 1.9051797
C -2.5810139 4.5317713 1.8318913
C -2.7501839 3.1577888 1.7977713
C -3.7455616 5.4706560 1.7875408
H 0.7872620 4.4596115 1.9690583
H -1.0670073 6.0743949 1.9419824
H -3.7520792 2.7508815 1.7503096
H -0.5190890 -4.6482693 1.6365049
H -4.7407569 -3.9887352 1.8235051
H -4.2693470 -1.5770595 1.9145061
H 3.4864863 -2.9370065 1.8855001
H 5.8115629 -2.1380752 1.8351308
H 4.2736323 1.8507845 1.7087152
H -4.6779192 4.9464598 1.5844735
H -3.8589705 5.9960017 2.7372150
H -3.6070443 6.2294041 1.0161037
H 6.6353551 1.5548935 1.4956201
H 7.1462394 0.3212723 2.6435677
H 7.2068818 -0.0135781 0.9217822
H -1.9577962 -6.5420158 1.4092946
H -3.2769734 -6.3885769 2.5656483
H -3.6039854 -6.2408074 0.8476301
N -1.1126530 2.5879952 -1.5559854
N -2.1693260 1.7831793 -1.5311534
C -3.2721510 2.5313530 -1.5537402
C -2.9270749 3.8683433 -1.6007860
C -1.5443061 3.8493477 -1.6007416
Au -1.9416431 -0.2291839 -1.5217112
Au 0.7595544 1.8161694 -1.5043281
Au 1.1804261 -1.5456443 -1.5455150
N -0.4486021 -2.7481756 -1.5934729
N -1.6736626 -2.2346695 -1.6158232
C -2.5499736 -3.2375576 -1.6905789
C -1.8752250 -4.4444668 -1.7136671
C -0.5451478 -4.0763803 -1.6478095
C -4.0158177 -3.0185553 -1.8374466
C 0.6535264 -4.9586737 -1.6260948

C	-4.6349464	1.9335134	-1.5208301
C	-0.6241673	5.0118655	-1.7419195
N	2.7840659	-0.3095063	-1.6006214
N	2.6158506	1.0075858	-1.5568707
C	3.8148947	1.5888289	-1.5898590
C	4.7993261	0.6221882	-1.6632185
C	4.0913874	-0.5657651	-1.6675544
C	4.6358161	-1.9421166	-1.8331125
C	3.9794359	3.0674125	-1.5411386
H	-3.5796541	4.7228850	-1.6314383
H	-2.2888795	-5.4357590	-1.7707810
H	5.8651147	0.7604635	-1.7077902
F	5.9434909	-1.9495024	-1.5647702
F	4.0495435	-2.8319019	-1.0332449
F	4.4776998	-2.3771138	-3.0850702
F	0.2795444	-6.2371084	-1.6935987
F	1.4697198	-4.7159793	-2.6521955
F	1.3677834	-4.8052432	-0.5076373
F	5.2740928	3.3836401	-1.5933761
F	3.4810772	3.5898014	-0.4171954
F	3.3689608	3.6706739	-2.5615706
F	-4.6730705	-4.1517272	-1.5801661
F	-4.3264578	-2.6445191	-3.0805255
F	-4.4850541	-2.0788517	-1.0177602
F	-5.5562812	2.8972275	-1.5559380
F	-4.8490476	1.1241546	-2.5586830
F	-4.8411213	1.2177819	-0.4119169
F	-1.2717313	6.1428692	-1.4528589
F	0.4379983	4.9330598	-0.9413710
F	-0.1660019	5.1166657	-2.9911911

1f•2b:

87

C	2.6868970	0.9654925	1.6945617
N	2.8294093	-0.3784042	1.6538773
C	4.0452383	-0.9309279	1.7191513
C	5.1954222	-0.1874291	1.8442201
C	5.1013723	1.1994314	1.9092672
C	3.8322413	1.7520568	1.8296846
Au	1.1096890	-1.5741693	1.6066848
Au	0.8531806	1.7679698	1.6190059
Au	-1.9145962	-0.1294194	1.6383746
N	-1.7358473	-2.2170468	1.6671925
C	-0.5000978	-2.7630940	1.6749196
C	-0.3860418	-4.1520005	1.7697822
C	-1.4953666	-4.9792526	1.8390970
C	-2.7462682	-4.3682928	1.8127504
C	-2.8200811	-2.9985245	1.7290504
C	-1.3620586	-6.4678793	1.9078975
C	6.3203017	2.0590904	2.0276899
C	-2.1350108	1.8605296	1.7044534
N	-1.0441454	2.6561386	1.6569317
C	-1.1757860	3.9869090	1.7051296
C	-2.3953590	4.6107145	1.8142987
C	-3.5495757	3.8346211	1.8837733
C	-3.3910884	2.4594002	1.8277955
C	-4.9014455	4.4673816	1.9862876
H	-0.2642673	4.5668999	1.6543541
H	-2.4409138	5.6912342	1.8456302
H	-4.2660352	1.8240582	1.8763843
H	0.6030909	-4.5913324	1.7851740

H -3.6576241 -4.9496089 1.8572743

H -3.7796440 -2.4995539 1.7113986

H 4.0911921 -2.0107814 1.6726260

H 6.1524682 -0.6895246 1.8939701

H 3.7202000 2.8279715 1.8738726

H -5.6613054 3.7463259 2.2813190

H -4.9062837 5.2826073 2.7096745

H -5.1987227 4.8845282 1.0232580

H 6.1030619 2.9921001 2.5455390

H 7.1219690 1.5513899 2.5624505

H 6.6970013 2.3154735 1.0364439

H -0.3625311 -6.7677828 2.2172122

H -2.0790979 -6.9019952 2.6041360

H -1.5497938 -6.9096996 0.9281834

N -0.2227605 2.6382699 -1.4879556

N -1.4762716 2.2030364 -1.5163831

C -2.2792482 3.2510446 -1.7046608

C -1.5329607 4.4125502 -1.8019488

C -0.2345876 3.9622047 -1.6561573

Cu -1.8792709 0.3494064 -1.1896617

Cu 1.2339298 1.4122991 -1.2141018

Cu 0.5939032 -1.8052675 -1.2164803

N -1.2139977 -2.3850729 -1.5398925

N -2.2214231 -1.5231474 -1.4684885

C -3.3626406 -2.1975844 -1.6229971

C -3.1006053 -3.5421340 -1.8050634

C -1.7195936 -3.6026921 -1.7427353

C -4.6812904 -1.5191414 -1.5703776

C -0.8575494 -4.7914068 -1.9594290

C -3.7454813 3.0986375 -1.8793960

C 1.0204976 4.7538272 -1.6533990

N 2.3897663 -1.1645124 -1.4844313

N 2.6423710 0.1384148 -1.5260472

C 3.9544661 0.3047031 -1.6970211

C 4.5863514 -0.9247015 -1.7688302

C 3.5435381 -1.8202429 -1.6265760

C 3.5986123 -3.3030328 -1.6037770

C 4.5660094 1.6450959 -1.8779424

H -1.8802964 5.4187230 -1.9581931

H -3.7981035 -4.3464327 -1.9601719

H 5.6334911 -1.1304343 -1.9055227

F 4.8495465 -3.7302395 -1.7786015

F 3.1667206 -3.7992094 -0.4347375

F 2.8427666 -3.8486632 -2.5583225

F 0.2441555 -4.7637550 -1.2055413

F -1.5243160 -5.9132247 -1.6667230

F -0.4585426 -4.8909177 -3.2299069

F 3.9425940 2.5874688 -1.1664931

F 4.5362676 2.0353176 -3.1547990

F 5.8493923 1.6313927 -1.5022969

F -5.6671778 -2.3998186 -1.7456218

F -4.8029460 -0.5782290 -2.5081318

F -4.8867850 -0.9188849 -0.3886455

F -4.2463184 2.1160695 -1.1261345

F -4.3798411 4.2289082 -1.5510310

F -4.0667523 2.8217147 -3.1457533

F 0.7672344 6.0374749 -1.9095150

F 1.6441954 4.6997760 -0.4667859

F 1.8892028 4.3168052 -2.5668556

1f•2c:

87

C	2.7693421	-0.2386315	1.7866157
N	2.3385237	-1.5195229	1.8018458
C	3.2176256	-2.5284007	1.8011732
C	4.5771650	-2.3301631	1.7916522
C	5.0729697	-1.0286038	1.7891829
C	4.1461238	0.0010432	1.7890911
Au	0.2809339	-1.8946693	1.8131566
Au	1.4339232	1.2546550	1.7543095
Au	-1.8684117	0.6768873	1.7435760
N	-2.5738568	-1.2919619	1.7818458
C	-1.6802855	-2.3042383	1.8392149
C	-2.1617297	-3.6138100	1.9077970
C	-3.5169015	-3.9021572	1.9024187
C	-4.3957472	-2.8244876	1.8261494
C	-3.8869497	-1.5490901	1.7714164
C	-4.0252297	-5.3078063	1.9642765
C	6.5455720	-0.7662979	1.7678281
C	-1.2438214	2.5804531	1.7525709
N	0.0808857	2.8495895	1.7469211
C	0.5135607	4.1156127	1.7416476
C	-0.3394401	5.1931870	1.7499196
C	-1.7139830	4.9697764	1.7694283
C	-2.1405265	3.6514936	1.7717400
C	-2.6808183	6.1113286	1.7575885
H	1.5852994	4.2618813	1.7248915
H	0.0677621	6.1955689	1.7367162
H	-3.2020057	3.4391744	1.7739385
H	-1.4483986	-4.4270477	1.9512288
H	-5.4673124	-2.9725964	1.8035896
H	-4.5479981	-0.6949814	1.7096549
H	2.8074869	-3.5292401	1.8014693
H	5.2400744	-3.1851078	1.7799562
H	4.4941097	1.0260109	1.7742010
H	-3.7057228	5.7718674	1.8945961
H	-2.4533486	6.8297777	2.5452671
H	-2.6315910	6.6446328	0.8075044
H	6.7646127	0.2994336	1.7641309
H	7.0333117	-1.2084297	2.6373711
H	7.0044149	-1.2052441	0.8814866
H	-3.2130977	-6.0311279	1.9249222
H	-4.5824039	-5.4783204	2.8864858
H	-4.7005817	-5.5157530	1.1345266
N	-2.8163601	-0.4281830	-1.4545080
N	-2.3443493	-1.6648548	-1.4959223
C	-3.3696896	-2.4998352	-1.6676943
C	-4.5563686	-1.7891835	-1.7422612
C	-4.1414144	-0.4779990	-1.5987482
Ag	-0.2679730	-2.0464955	-1.1487785
Ag	-1.5359169	1.2719106	-1.2187837
Ag	1.9577345	0.7290251	-1.1851415
N	2.6585112	-1.2644757	-1.5244681
N	1.8423475	-2.3008102	-1.4041810
C	2.5559825	-3.4179249	-1.5510310
C	3.8845307	-3.1090057	-1.7793060
C	3.8896953	-1.7244638	-1.7506423
C	1.9144554	-4.7527613	-1.4595332
C	5.0273072	-0.8109119	-2.0272466
C	-3.1725317	-3.9603101	-1.8512126
C	-4.9576080	0.7611534	-1.5798211
N	1.1380503	2.6908618	-1.4431992
N	-0.1677151	2.8807139	-1.5615927

C -0.3853993 4.1800246 -1.7685192

C 0.8159996 4.8686629 -1.7850051

C 1.7483584 3.8698602 -1.5705850

C 3.2250667 3.9812997 -1.4760607

C -1.7445686 4.7129402 -2.0390354

H -5.5545745 -2.1654115 -1.8831654

H 4.7104619 -3.7788459 -1.9434275

H 0.9826644 5.9213093 -1.9331856

F 3.6129589 5.2514828 -1.5854925

F 3.6849214 3.5187112 -0.3033601

F 3.8436827 3.2806330 -2.4308211

F 6.1924676 -1.4397445 -1.8473392

F 5.0068144 -0.3606532 -3.2848964

F 5.0219668 0.2663588 -1.2339082

F -1.7782316 6.0354973 -1.8495923

F -2.6749681 4.1672818 -1.2481337

F -2.1262675 4.4798963 -3.2979468

F 2.8139516 -5.7225197 -1.6190582

F 0.9656126 -4.9172348 -2.3858175

F 1.3233973 -4.9416294 -0.2696362

F -4.2886606 -4.6293174 -1.5464644

F -2.8613593 -4.2632440 -3.1147999

F -2.1881636 -4.4437308 -1.0861483

F -4.8811449 1.3916406 -0.3973084

F -4.5551149 1.6374271 -2.5050314

F -6.2420947 0.4881705 -1.8044925

2a•1f•2a:

132

C 2.7947917 0.1873359 0.0011614

N 2.5607304 -1.1450789 0.0040929

C 3.5752091 -2.0165313 0.0081918

C 4.8920807 -1.6250490 0.0092618

C 5.1886916 -0.2652701 0.0060401

C 4.1236623 0.6208825 0.0020301

Au 0.5814325 -1.8184891 0.0027696

Au 1.2473986 1.4628188 -0.0030339

Au -1.9302407 0.3957367 -0.0003788

N -2.3351453 -1.6554022 0.0022491

C -1.2960115 -2.5218843 0.0027328

C -1.5819667 -3.8900550 0.0031003

C -2.8807470 -4.3726180 0.0028454

C -3.9120063 -3.4384761 0.0026962

C -3.5956830 -2.1014868 0.0022443

C -3.1756569 -5.8391189 0.0030296

C 6.6072076 0.2089044 0.0066989

C -1.5984930 2.3730317 -0.0044856

N -0.3280595 2.8378690 -0.0067058

C -0.0825613 4.1525869 -0.0102216

C -1.0811120 5.0961530 -0.0115685

C -2.4066780 4.6716539 -0.0090780

C -2.6397678 3.3059646 -0.0056924

C -3.5277011 5.6617702 -0.0101938

H 0.9571293 4.4502378 -0.0118789

H -0.8236756 6.1468587 -0.0143466

H -3.6599981 2.9452779 -0.0038409

H -0.7590593 -4.5928649 0.0032951

H -4.9502577 -3.7420858 0.0024365

H -4.3741925 -1.3507494 0.0014673

H 3.3117534 -3.0653148 0.0108365

H	5.6726864	-2.3742447	0.0126733
H	4.3231927	1.6844722	-0.0002543
H	-4.5000259	5.1725012	-0.0051617
H	-3.4723294	6.3134528	0.8633760
H	-3.4774987	6.3055036	-0.8899586
H	6.6709875	1.2957464	0.0098473
H	7.1419194	-0.1643706	0.8817535
H	7.1406263	-0.1588990	-0.8715189
H	-2.2647889	-6.4351451	-0.0043818
H	-3.7564137	-6.1189778	0.8836461
H	-3.7696994	-6.1167792	-0.8693053
N	2.7904063	-0.4014266	-3.3611129
N	2.6511145	0.9186780	-3.3029584
C	3.8623367	1.4737237	-3.3274352
C	4.8255908	0.4872177	-3.4127044
C	4.0919648	-0.6849592	-3.4319847
Au	0.8138189	1.7704978	-3.2891301
Au	1.1610495	-1.6014761	-3.2731432
Au	-1.9253253	-0.2178325	-3.2918518
N	-2.1112375	1.7978006	-3.3296233
N	-1.0399912	2.5815520	-3.3844781
C	-1.4489954	3.8487348	-3.4666478
C	-2.8312052	3.8931389	-3.4584685
C	-3.2002399	2.5649659	-3.3685005
C	-0.5121234	4.9896106	-3.6643428
C	-4.5727248	1.9927259	-3.3059163
C	4.0573226	2.9471615	-3.2525952
C	4.6092887	-2.0687879	-3.6206841
N	-1.7044629	-2.2305462	-3.3574901
N	-0.4923234	-2.7699001	-3.2864194
C	-0.6177673	-4.0966152	-3.2938586

C	-1.9536419	-4.4375464	-3.3817119
C	-2.6012095	-3.2162116	-3.4198044
C	-4.0572554	-2.9730332	-3.6172413
C	0.5590481	-5.0021684	-3.1916300
H	5.8940661	0.6033505	-3.4562246
H	-3.4685046	4.7578728	-3.5144850
H	-2.3879799	-5.4211204	-3.4138294
F	-4.7486069	-4.0723262	-3.3085685
F	-4.5237503	-1.9738233	-2.8697094
F	-4.3276066	-2.6702548	-4.8884002
F	-5.4783640	2.9642640	-3.4188738
F	-4.7908566	1.1137384	-4.2840793
F	-4.7979359	1.3622508	-2.1494703
F	0.1605751	-6.2738766	-3.2247222
F	1.2294799	-4.8184228	-2.0501854
F	1.4220276	-4.8166732	-4.1903997
F	-1.1384116	6.1409910	-3.4123412
F	-0.0701369	5.0365604	-4.9226652
F	0.5601015	4.9269370	-2.8760301
F	5.3575111	3.2383607	-3.2925983
F	3.4636892	3.5804283	-4.2643931
F	3.5629938	3.4580915	-2.1214102
F	5.9142740	-2.1072476	-3.3426406
F	3.9997160	-2.9632436	-2.8438829
F	4.4524208	-2.4749073	-4.8821656
N	2.7927644	-0.3758736	3.3653826
N	2.6539649	0.9439809	3.3002121
C	3.8652789	1.4988462	3.3239726
C	4.8281087	0.5126002	3.4160644
C	4.0941339	-0.6592944	3.4400665
Au	0.8170063	1.7963579	3.2814650

Au 1.1628548 -1.5756194 3.2800126

Au -1.9231450 -0.1908493 3.2921187

N -2.1081486 1.8250042 3.3237617

N -1.0364682 2.6085050 3.3730750

C -1.4447001 3.8762076 3.4507117

C -2.8269171 3.9212027 3.4453332

C -3.1967359 2.5928029 3.3617854

C -0.5066082 5.0173971 3.6407612

C -4.5695998 2.0208172 3.3046370

C 4.0606806 2.9718297 3.2419668

C 4.6114780 -2.0418361 3.6378409

N -1.7029318 -2.2034186 3.3635895

N -0.4909175 -2.7434172 3.2951051

C -0.6169745 -4.0700883 3.3048459

C -1.9531163 -4.4102343 3.3914836

C -2.6001999 -3.1885822 3.4265184

C -4.0563460 -2.9444277 3.6219146

C 0.5593097 -4.9767294 3.2061774

H 5.8965505 0.6286451 3.4608244

H -3.4637148 4.7864509 3.4990920

H -2.3879134 -5.3935523 3.4248867

F -4.7477989 -4.0442050 3.3153835

F -4.3278070 -2.6382913 4.8920367

F -4.5217493 -1.9469169 2.8713940

F -5.4745754 2.9930501 3.4170019

F -4.7981692 1.3868039 2.1508023

F -4.7854338 1.1449448 4.2860844

F 0.1598952 -6.2480503 3.2427972

F 1.4216405 -4.7888622 4.2050709

F 1.2308017 -4.7970489 2.0647103

F -1.1332651 6.1680555 3.3863328

F 0.5628249 4.9510407 2.8488790

F -0.0600731 5.0691622 4.8972913

F 5.3609132 3.2629321 3.2813135

F 3.5671436 3.4772198 2.1079573

F 3.4665839 3.6101764 4.2502976

F 5.9151028 -2.0835677 3.3536205

F 4.4609945 -2.4367037 4.9036863

F 3.9974082 -2.9425853 2.8720732

2b•1f•2b:

132

C 2.5317383 1.2418737 0.0574659

N 2.8247040 -0.0793762 0.0512211

C 4.0943967 -0.4850453 0.0947668

C 5.1544697 0.3941610 0.1509362

C 4.9048252 1.7602841 0.1611086

C 3.5754285 2.1606943 0.1106838

Au 1.2437239 -1.4575940 -0.0012976

Au 0.6179037 1.8296906 0.0077686

Au -1.9156770 -0.3497656 -0.0534852

N -1.5132387 -2.4083631 -0.0671297

C -0.2248890 -2.8194789 -0.0420712

C 0.0440253 -4.1857487 -0.0369876

C -0.9703869 -5.1337812 -0.0555872

C -2.2770158 -4.6618312 -0.0849637

C -2.5030689 -3.3031635 -0.0898932

C -0.6629798 -6.5970774 -0.0643398

C 6.0091645 2.7668489 0.2151224

C -2.3641568 1.6021195 -0.0406450

N	-1.3649747	2.5139941	-0.0277384
C	-1.6467459	3.8179475	-0.0156728
C	-2.9375648	4.2996672	-0.0070560
C	-3.9978477	3.4022085	-0.0123098
C	-3.6817147	2.0500498	-0.0351504
C	-5.4206349	3.8625331	-0.0084552
H	-0.8058931	4.4987593	-0.0068532
H	-3.1059255	5.3679765	0.0113657
H	-4.4804982	1.3192681	-0.0402801
H	1.0753481	-4.5137513	-0.0123513
H	-3.1193087	-5.3402804	-0.0973251
H	-3.5120498	-2.9137212	-0.1076580
H	4.2659198	-1.5531763	0.0885616
H	6.1637684	0.0072735	0.1908243
H	3.3400437	3.2172114	0.1186874
H	-6.0352050	3.2407292	0.6408191
H	-5.5111504	4.8951328	0.3242619
H	-5.8406346	3.7998075	-1.0129698
H	5.8130437	3.5230954	0.9738972
H	6.9691020	2.3045139	0.4370598
H	6.1000585	3.2827039	-0.7408915
H	0.2090319	-6.8232609	0.5470791
H	-1.4997214	-7.1882635	0.3039636
H	-0.4451552	-6.9291417	-1.0800429
N	-0.2709685	2.6649547	-3.0876681
N	-1.4930818	2.1591580	-3.2140899
C	-2.3381906	3.1632173	-3.4505571
C	-1.6552516	4.3665805	-3.4760942
C	-0.3478158	3.9893186	-3.2374650
Cu	-1.8229240	0.2901446	-2.8892574
Cu	1.2371122	1.5005172	-2.8206491
Cu	0.7477573	-1.7540631	-2.8603424
N	-1.0198550	-2.4041660	-3.2563043
N	-2.0710140	-1.5976983	-3.1574956
C	-3.1747310	-2.3207040	-3.3586574
C	-2.8425926	-3.6389939	-3.6047790
C	-1.4608698	-3.6333171	-3.5278695
C	-4.5258716	-1.7094026	-3.2970407
C	-0.5362711	-4.7620394	-3.8030293
C	-3.7779131	2.9377747	-3.7348081
C	0.8516846	4.8572751	-3.1271156
N	2.5183514	-1.0273443	-3.0399873
N	2.6998861	0.2859963	-3.1330588
C	4.0002151	0.5146159	-3.3206134
C	4.6972243	-0.6807475	-3.3468716
C	3.7061556	-1.6247658	-3.1611549
C	3.8487512	-3.1001749	-3.0794897
C	4.5432050	1.8752324	-3.5615155
H	-2.0485691	5.3541185	-3.6421042
H	-3.4967786	-4.4685040	-3.8085468
H	5.7537367	-0.8347210	-3.4799495
F	5.1327959	-3.4507037	-3.1448030
F	3.3590147	-3.5822896	-1.9279368
F	3.2045090	-3.7236172	-4.0670005
F	0.5541302	-4.7230177	-3.0320869
F	-1.1454599	-5.9317354	-3.5889065
F	-0.1177956	-4.7597076	-5.0706272
F	3.8730899	2.8151446	-2.8889629
F	4.4902172	2.2072975	-4.8533651
F	5.8246522	1.9417316	-3.1888838
F	-5.4680222	-2.6374535	-3.4595633
F	-4.7003878	-0.7814119	-4.2391581

F -4.7471826 -1.1136624 -2.1159844

F -4.2773562 1.9133577 -3.0377227

F -4.4924483 4.0249181 -3.4301217

F -3.9926263 2.6729384 -5.0254641

F 0.5113440 6.1417993 -3.2299399

F 1.4695688 4.6988168 -1.9473156

F 1.7524368 4.5967813 -4.0758936

N -0.3732035 2.6326960 3.0773164

N -1.5979710 2.1234905 3.1595488

C -2.4521512 3.1237869 3.3793830

C -1.7725363 4.3277422 3.4394786

C -0.4576053 3.9553951 3.2382735

Cu -1.9088860 0.2549601 2.8114942

Cu 1.1464827 1.4715970 2.8608279

Cu 0.6671992 -1.7885445 2.8226355

N -1.1163124 -2.4528035 3.1249284

N -2.1623257 -1.6381385 3.0335267

C -3.2731809 -2.3645673 3.1791464

C -2.9513820 -3.6926920 3.3775550

C -1.5679008 -3.6900141 3.3331681

C -4.6214881 -1.7483157 3.1083063

C -0.6624159 -4.8415807 3.5763952

C -3.9007551 2.8973032 3.6145723

C 0.7423680 4.8273960 3.1764175

N 2.4214532 -1.0569909 3.1197668

N 2.5951795 0.2558184 3.2357663

C 3.8798558 0.4831038 3.5120571

C 4.5743137 -0.7122074 3.5760563

C 3.5991218 -1.6547215 3.3159545

C 3.7495452 -3.1293028 3.2352616

C 4.4104690 1.8402721 3.7960110

H -2.1731965 5.3129321 3.6020319

H -3.6124186 -4.5278171 3.5304987

H 5.6199031 -0.8665498 3.7777146

F 5.0338918 -3.4743027 3.3249748

F 3.0908701 -3.7576588 4.2102897

F 3.2836202 -3.6107182 2.0738327

F -1.2086803 -5.9758875 3.1288765

F 0.5193673 -4.6918451 2.9727608

F -0.4187964 -5.0102877 4.8782757

F 5.7005905 1.9206085 3.4576653

F 4.3238835 2.1432195 5.0932887

F 3.7532837 2.7922598 3.1277077

F -5.5684421 -2.6746981 3.2512953

F -4.8280362 -1.1408423 1.9304151

F -4.8032282 -0.8290324 4.0574649

F -4.6092543 3.9729225 3.2608601

F -4.3692939 1.8549571 2.9227047

F -4.1605628 2.6591611 4.9022551

F 0.3929066 6.1109271 3.2597298

F 1.6021006 4.5742889 4.1643379

F 1.4114982 4.6675089 2.0249844

2c•1f•2c:

132

C 3.1867654 -0.0345923 -0.1698180

N 2.8373764 -1.3414917 -0.1437703

C 3.7770684 -2.2903572 -0.1747883

C 5.1223604 -2.0033077 -0.2275119

C 5.5327150 -0.6756455 -0.2455312

C	4.5399842	0.2944790	-0.2205138
Au	0.8059986	-1.8383581	-0.0669973
Au	1.7555676	1.3690442	-0.1408959
Au	-1.4933944	0.5845305	0.1291011
N	-2.0800822	-1.4222671	0.0949870
C	-1.1245765	-2.3754233	0.0081771
C	-1.5147213	-3.7148844	-0.0158939
C	-2.8469459	-4.0923799	0.0526995
C	-3.7918465	-3.0736779	0.1348866
C	-3.3709667	-1.7650015	0.1496516
C	-3.2623537	-5.5281824	0.0296319
C	6.9794096	-0.2987114	-0.2806882
C	-0.9882713	2.5267634	0.0985236
N	0.3075997	2.8724832	-0.0844122
C	0.6574734	4.1577210	-0.1751126
C	-0.2526663	5.1840378	-0.0620971
C	-1.5920010	4.8816445	0.1517068
C	-1.9378305	3.5379646	0.2166959
C	-2.6300710	5.9496928	0.2825792
H	1.7048279	4.3673489	-0.3441016
H	0.0871477	6.2076771	-0.1407167
H	-2.9755871	3.2652460	0.3581954
H	-0.7499722	-4.4778038	-0.0857094
H	-4.8500644	-3.2911738	0.1882668
H	-4.0879148	-0.9577804	0.2095489
H	3.4345073	-3.3161002	-0.1654701
H	5.8381555	-2.8136570	-0.2603516
H	4.8206240	1.3395364	-0.2545814
H	-3.2386793	5.7971733	1.1736714
H	-2.1863444	6.9419024	0.3405960
H	-3.3043874	5.9353068	-0.5745368

H	7.1553021	0.5327780	-0.9618239
H	7.3082458	0.0171934	0.7103029
H	7.6089927	-1.1308526	-0.5910151
H	-2.4049139	-6.1965843	0.0676275
H	-3.9123359	-5.7621449	0.8725119
H	-3.8211431	-5.7508521	-0.8796012
N	-2.6804106	-0.4511712	-3.0082208
N	-2.1562277	-1.6524042	-3.2065180
C	-3.1532113	-2.5207780	-3.3768213
C	-4.3733515	-1.8713097	-3.2860604
C	-4.0095931	-0.5588552	-3.0487695
Ag	-0.0512592	-1.9669873	-3.0060418
Ag	-1.4523170	1.2827489	-2.8833896
Ag	2.0660428	0.8585568	-3.1327974
N	2.8426958	-1.1040910	-3.4416287
N	2.0536660	-2.1616072	-3.3145124
C	2.8013050	-3.2604621	-3.4284671
C	4.1231865	-2.9174988	-3.6407700
C	4.0897356	-1.5325300	-3.6373946
C	2.1968377	-4.6109585	-3.3148194
C	5.2165547	-0.6035033	-3.9102395
C	-2.9036840	-3.9455819	-3.7147874
C	-4.8815777	0.6258289	-2.8476224
N	1.1492336	2.7754435	-3.3179822
N	-0.1670650	2.9306734	-3.2985760
C	-0.4394490	4.2229982	-3.4786719
C	0.7352202	4.9431029	-3.6218286
C	1.7109788	3.9701624	-3.5084834
C	3.1870652	4.1202333	-3.5582139
C	-1.8320642	4.7267364	-3.5923668
H	-5.3616454	-2.2863944	-3.3797016

H	4.9710989	-3.5657331	-3.7782511
H	0.8569956	5.9999010	-3.7843538
F	3.5278929	5.3857129	-3.7913833
F	3.7610113	3.7523266	-2.4018972
F	3.7338293	3.3631097	-4.5138499
F	6.3724608	-1.1258116	-3.4916962
F	5.3447719	-0.3532535	-5.2160994
F	5.0618598	0.5782824	-3.3036358
F	-1.8954069	6.0176190	-3.2581512
F	-2.6790961	4.0592647	-2.7998553
F	-2.3006999	4.6127627	-4.8378570
F	3.1171657	-5.5585412	-3.4798991
F	1.2370533	-4.8055750	-4.2233843
F	1.6310790	-4.8022257	-2.1124938
F	-3.9833765	-4.6863497	-3.4503709
F	-2.6163613	-4.1042934	-5.0093059
F	-1.8792089	-4.4620572	-3.0271823
F	-4.6549985	1.2144135	-1.6630289
F	-4.6792244	1.5593348	-3.7819565
F	-6.1666253	0.2802156	-2.8854022
N	-3.1169060	-1.3405353	3.2721203
N	-2.2862908	-2.3735648	3.3066139
C	-2.9968562	-3.4732822	3.5545123
C	-4.3388716	-3.1561646	3.6845594
C	-4.3538909	-1.7866704	3.4953195
Ag	-0.2005316	-2.1013674	2.9441206
Ag	-2.3687615	0.6361681	3.1018852
Ag	1.1017017	1.2178365	2.9372093
N	2.3723538	-0.4754920	3.1516176
N	1.8852311	-1.7072940	3.1099866
C	2.9030611	-2.5657749	3.1765212
C	4.1000861	-1.8790171	3.2656736
C	3.7004950	-0.5536868	3.2465132
C	2.6650645	-4.0307902	3.1611141
C	4.5456416	0.6551325	3.4243084
C	-2.3467975	-4.7960492	3.7409350
C	-5.5099265	-0.8558516	3.5298468
N	-0.2694572	2.8139517	3.2687107
N	-1.5673805	2.5758945	3.3943555
C	-2.1877005	3.7317405	3.6290733
C	-1.2708022	4.7663345	3.6617975
C	-0.0676213	4.1222465	3.4262231
C	1.2967975	4.7095177	3.4341359
C	-3.6574411	3.7821389	3.8314134
H	-5.1663307	-3.8133534	3.8879284
H	5.0971943	-2.2761438	3.3389681
H	-1.4465606	5.8141831	3.8327517
F	1.2506783	6.0165864	3.1746942
F	1.8926045	4.5545524	4.6199598
F	2.0989735	4.1407780	2.5258270
F	5.8206380	0.3865777	3.1341332
F	4.1554078	1.6729115	2.6501052
F	4.5072661	1.0983593	4.6848900
F	-4.0830391	5.0441550	3.8945034
F	-4.0287536	3.1671395	4.9565058
F	-4.3181230	3.1819764	2.8302947
F	3.8155472	-4.7006166	3.1854022
F	1.9958864	-4.4165458	2.0628073
F	1.9348131	-4.4265317	4.2077553
F	-3.2410659	-5.7799129	3.6243092
F	-1.3795539	-5.0152281	2.8430409
F	-1.7838127	-4.9051999	4.9467377

F	-6.6580680	-1.5288768	3.5741670
F	-5.4687429	-0.0466637	4.5916131
F	-5.5400947	-0.0663155	2.446355

8 STM measurements

Scanning tunnelling microscopy (STM) was performed under ambient conditions (room temperature) at the solution/solid interface, using 1-phenyloctane (PHO) as solvent and highly oriented pyrolytic graphite (HOPG) as substrate. The experimental setup consisted of an Agilent 5500 scanning probe microscope, that was placed on a Halcyonics actively isolated microscopy workstation. It was acoustically shielded with a home-built box. Scissor-cut Pt/Ir (80/20) tips were used and further modified after approach by applying short voltage pulses until the desired resolution was achieved. HOPG was obtained from Mikromasch and TipsNano in ZYB quality. All STM images (unless otherwise noted) were calibrated by subsequent immediate acquisition of an additional image at reduced bias voltage, therefore the atomic lattice of the HOPG surface was observed, which was used as a calibration grid. Data processing, also for image calibration, was performed using the SPIP 5 (Image Metrology) software package. (Supra-) molecular models were constructed using Spartan '08 (Wavefunction, Inc.).

Three different sample preparation methods were used for performing the STM measurements presented here:

Method A (pure **1e**): A solution of **1e** in PHO was applied to HOPG at elevated temperature (80 °C), kept at this temperature for 20 s, and allowed to cool to rt before the STM measurements were performed with the tip immersed into the solution.

Method B (addition of **2c** to a monolayer of **1e**): 0.5 µL of a $c_{\mathbf{1e}} = 3\times10^{-6}$ M solution of **1e** was dropped onto the HOPG substrate at elevated temperature (80 °C), kept at this temperature for 20 s, and allowed to cool to rt. Additional 0.5 µL of a 10^{-3} M or 10^{-4} M solution of **2c** in PHO were added to the as-prepared monolayer, and the STM measurements were performed with the tip immersed into the solution. Consequently, the effective concentrations have to be corrected for the volume increase, and final concentrations are $c_{\mathbf{1e}} = 1.5\times10^{-6}$ M and $c_{\mathbf{2c}} = 5\times10^{-4}$ M (or 5×10^{-5} M).

Method C (co-adsorption of a mixture of **2c** and **1e**): 0.5 µL of a mixture of **1e** and **2c** in PHO was dropped onto HOPG substrate at elevated temperature (80 °C), kept at this temperature for 20 s, and allowed to cool to rt, and the STM measurements were performed with the tip immersed into the solution. Concentrations of **1e** and **2c** in the mixture are given.

All STM measurements were typically completed after 1 h after the aforementioned sample preparation procedures.

8.1 Concentration-dependent polymorphism

1e formed self-assembled monolayers at the interface of 1-phenyloctane (PHO) and highly oriented pyrolytic graphite (HOPG). At a (starting) concentration of 1×10^{-6} M to 3×10^{-6} M of

1e in the supernatant solution, a porous polymorph A is observed (overview STM images: Figure S 57a and b, high-resolution STM image: Figure S 57d and Figure 4 (Main Text)) to which a unit cell (containing two molecules) with lattice constants of $a = b = (5.6\pm0.2)$ nm, $\gamma(a,b) = (60\pm2)°$ was indexed. All alkyl side chains of each molecule are aligned along the HOPG main axis directions, so that the unit cell vector a is oriented at an angle of $\gamma(a,d_1) = (15\pm1)°$ relative to the HOPG main axis direction d_1. Each of the C_{3v}-symmetric molecules (A) interacts with three adjacent molecules (B) by interdigitation of alkyl side chains in an ABAB packing scheme (cf. supramolecular and schematic models, Figure S 57e and f, respectively). A coordination number of CN = 3 (with respect to side chain interactions) results, and the overall packing can be viewed as a chiral honeycomb network.

With increasing concentration of **1e** in the supernatant liquid phase, a more densely packed polymorph B was observed alongside with polymorph A (e.g., coexistence of both polymorphs A and B at $c = 7\times10^{-6}$ M as seen in the overview image in Figure S 57c). The surface coverage of polymorph B gradually increases, and polymorph B is solely observed at a concentration as high as 1×10^{-5} M (see high-resolution STM image in Figure S 57g). Two of the six alkyl side chains of each molecule of **1e** point towards the solution phase, so that four alkyl side chains of each molecule are adsorbed on the HOPG surface and are aligned along two HOPG main axis directions so that hexagonal assemblies, formed from six molecules, are observed (schematic model in Figure S 57i), in which each two molecules interact via interdigitating alkyl side chains (with an ABAB packing scheme). These hexagonal assemblies pack densely to form a hexagonal pattern (Figure S 57h and i), to which a unit cell of $a = b = (7.1\pm0.2)$ nm, $\gamma(a,b) = (60\pm2)°$ (containing six molecules) is indexed. The unit cell vector a is oriented at an angle of $\gamma(a,d_1) = (19\pm2)°$ relative to the HOPG main axis direction, d_1. As a result of the hierarchical packing of hexagonal units (each formed by six molecules), triples of bright spots, containing three neighboring backbones, are observed. The bright regions in the interior of some of the pores (indicated by arrow 1 in Figure S 57g) appeared blurred, which is attributed to additional, unspecifically adsorbed molecules. Even at this increased concentration, no evidence for the formation of multilayers, where molecules of **1e** stack on top of each other, is observed in any of the images acquired.

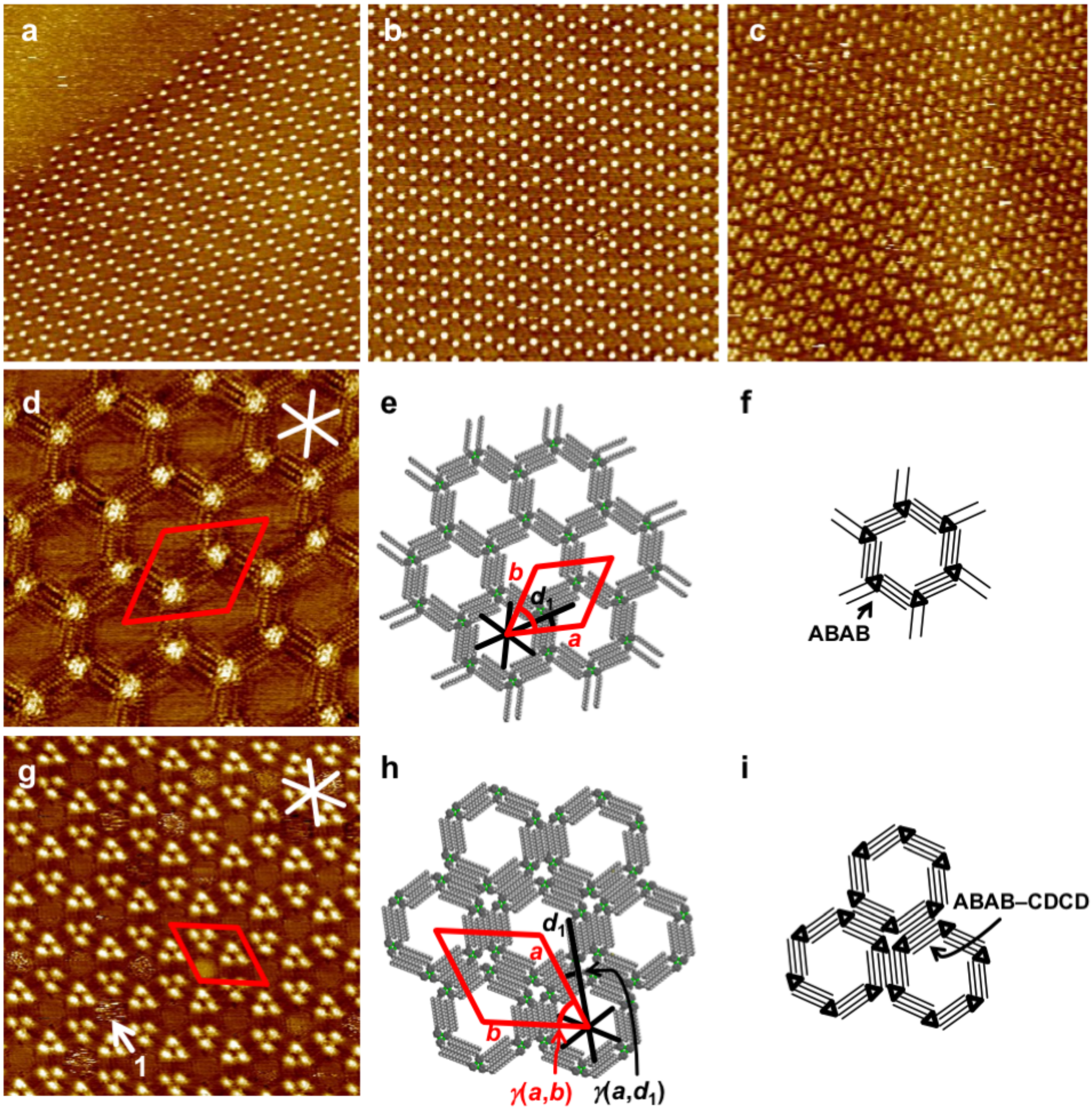

Figure S 57. (a)–(c), (d), (g) STM images, (e), (h) supramolecular, and (f), (i) schematic models of self-assembled monolayers of 1e at the PHO/HOPG interface. (a)–(c) Overview STM images (100×100 nm^2, internal scanner calibration) of (a),(b) polymorph A (a: $c = 1\times10^{-6}$ M, b: $c = 3\times10^{-6}$ M, both: $V_S = -0.8$ V, $I_t = 11$ pA) and (c) polymorph A and B ($c = 7\times10^{-6}$ M, $V_S = -0.8$ V, $I_t = 11$ pA); (d) high-resolution STM image, (e) supramolecular model, and (f) schematic model of polymorph A (c: 20.0×20.0 nm^2, $c = 1.5\times10^{-6}$ M, $V_S = -0.15$ V, $I_t = 24$ pA), $a = b = (5.6\pm0.2)$ nm, $\gamma(a,b) = (60\pm2)°$, $\gamma(a,d_1) = (15\pm1)°$; (g) high-resolution STM image, (h) supramolecular model, and (i) schematic model of polymorph B (g: 42.8×42.8 nm^2, $c = 1\times10^{-5}$ M, $V_S = -0.8$ V, $I_t = 10$ pA), $a = b = (5.6\pm0.2)$ nm, $\gamma(a,b) = (60\pm2)°$, $\gamma(a,d_1) = (15\pm1)°$. The red and white (black) lines indicate unit cells and HOPG main axis directions, respectively.

8.2 Co-adsorption of 2c on 1e

We next investigated whether the concept of π-acid-base interactions could be applied to template the co-adsorption of **2c** on a monolayer of **1e**. The lower concentration range of 1×10^{-6} M to 3×10^{-6} M for **1e** was chosen to make sure that all alkyl chains are aligned on the HOPG substrate.

Two different sample preparation methods, B and C, were used to investigate the coadsorption of **2c** on top of the backbones of **1e**.

When 0.5 µL of a solution of **1e** with $c_{\mathbf{1e}} = 3\times10^{-6}$ M was dropped onto the HOPG substrate at elevated temperature (80 °C), kept at this temperature for 20 s, and allowed to cool to r.t., and 0.5 µL of a 10^{-3} M solution of **2c** in PHO was added to the as-prepared monolayer (which was previously denoted as method B), the STM images shown in Figure S 58a–d were observed. Polymorph A of **1e** is observed to cover the HOPG surface, while few additional bright features are observed which are marked by green circles. When a mixture of **1e** ($c_{\mathbf{1e}}$ = 1.5×10^{-6} M) and **2c** ($c_{\mathbf{2c}} = 5\times10^{-4}$ M) was applied to the HOPG surface at r.t. (which was previously denoted as method C), the STM images shown in Figure S 58e–h were observed. The STM images again show the hexagonal packing of **1e** in polymorph A, while parts of the HOPG surface remaining uncovered, and – again – additional bright features. In both cases, the bright features are clearly localized above the backbone regions of **1e** (according to the lattice). We attribute these to the formation of supramolecular stacks, where **2c** adsorbs on top of the backbone of **1e**. Through its π-basicity, **1e** acts as a secondary template for the adsorption of π-acidic **2c**, dictated through the donor-acceptor interactions.

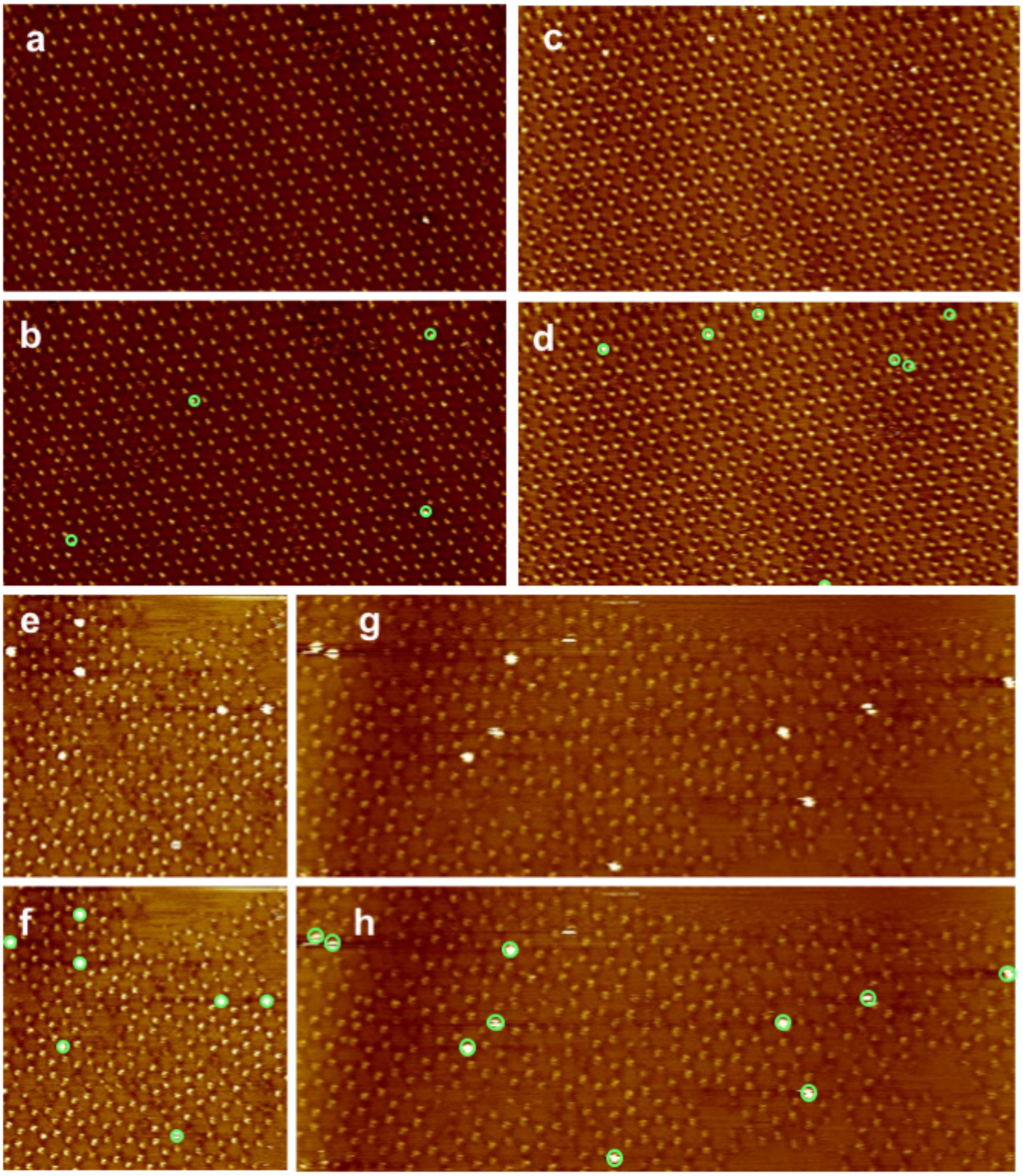

Figure S 58. STM images of self-assembled monolayers of 1e with coadsorbates of 2c (without and with markers), prepared by methods B and C. (a)–(d) Sample prepared by method B; 150×85 nm^2, c_{1e} = 1.5×10^{-6} M and c_{2c} = 5×10^{-4} M, V_S = −0.8 V, I_t = 10 pA; (e)–(h) sample prepared by method C; (e, f: 78×78 nm^2; g, h: 150×61 nm^2, c_{1e} = 1.5×10^{-6} M and c_{2c} = 5×10^{-4} M, V_S = −0.9 V, I_t = 18 pA. All image sizes are determined by internal scanner calibration.

In addition, we investigated whether **2c** forms self-assembled monolayers under similar experimental conditions as used for the co-adsorption experiments, but without the template monolayer of **1e**. Therefore, we prepared $10^{-3} - 10^{-6}$ M solutions of **2c** in PHO, applied 0.5 µL of the solutions (at r.t.) to freshly cleaved HOPG surfaces (without further thermal annealing). Even under the highest concentrations (10^{-3} M), no hints on any self-assembled monolayers or periodic patterns were observed by STM (Figure S 59).

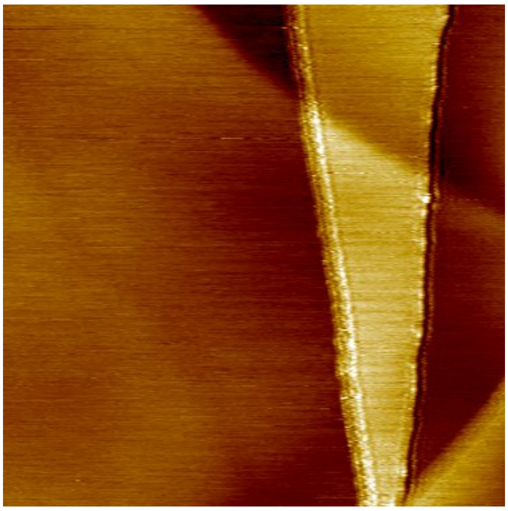

Figure S 59. Typical STM image of the uncovered HOPG surface after applying a solution of 2c in PHO (221×221 nm^2 (internal scanner calibration), c_{2c} = 10^{-3} M V_S = −0.8 V, I_t = 20 pA). No self-assembled monolayers are observed (natural step edges of the HOPG substrate are visible).

The results show that a template monolayer (such as 1**e**) is crucial for the adsorption of **2c** (here).

8.3 Adsorption of 1e on Au(111)

We were interested to find out whether **1e** forms self-assembled monolayers on Au(111). A substrate with several 10×10 nm^2 large terraces of Au(111) was obtained by flame-annealing commercially available glass slides covered with 250 nm gold with an 1 nm chromium adhesive layer (obtained from arrandee.com, Werther, Germany) with a butane flame according to literature procedures (see arrandee.com). After application of a 2.5×10^{-6} M solution of **1e** in PHO to the flame-annealed substrate at 80 °C, keeping at this temperature for 10 s and allowing the substrate to cool to r.t., we acquired the STM images shown in Figure S 60. Typical terraces of the Au(111) surface are seen, which are covered by medium bright features and bright features – the latter of which is attributed to the hexagonal packing of **1e** similar to the porous polymorph A that was observed on HOPG.

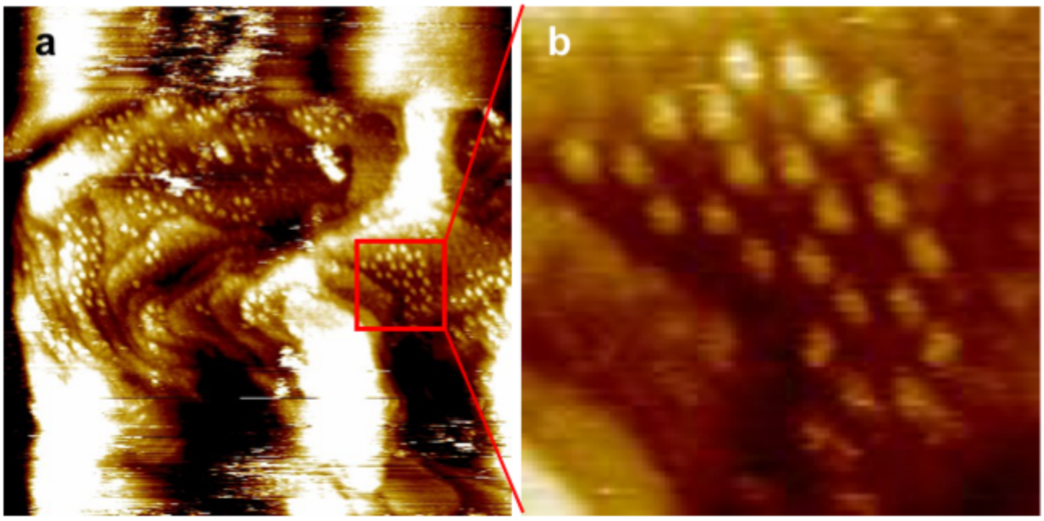

Figure S 60. (a) STM image of 1e (c = 2.5×10^{-6} M) on a Au(111), 200×200 nm^2, internal scanner calibration, V_S = –0.8 V, I_t = 5 pA), (b) enlarged region (36×36 nm^2) of (a).

9 References

[1] G. R. Fulmer, A. J. M. Miller, N. H. Sherden, H. E. Gottlieb, A. Nudelman, B. M. Stoltz, J. E. Bercaw, K. I. Goldberg, *Organometallics* **2010**, *29*, 2176–2179.

[2] M. Peters, M. Trobe, R. Breinbauer, *Chemistry* **2013**, *19*, 2450–2456.

[3] M. A. Omary, M. A. Rawashdeh-Omary, M. W. A. Gonser, O. Elbjeirami, T. Grimes, T. R. Cundari, H. V. K. Diyabalanage, C. S. P. Gamage, H. V. R. Dias, *Inorg. Chem.* **2005**, *44*, 8200–8210.

[4] H. . Rasika Dias, S. A. Polach, Z. Wang, *J. Fluor. Chem.* **2000**, *103*, 163–169.

[5] C. Nieto-Oberhuber, S. López, A. M. Echavarren, *J. Am. Chem. Soc.* **2005**, *127*, 6178–6179.

[6] Q. Chen, X. M. du Jourdin, P. Knochel, *J. Am. Chem. Soc.* **2013**, *135*, 4958–4961.

[7] K. Tamao, K. Sumitani, Y. Kiso, M. Zembayashi, A. Fujioka, S. Kodama, I. Nakajima, A. Minato, M. Kumada, *Bull. Chem. Soc. Jpn.* **1976**, *49*, 1958–1969.

[8] K. Akiba, Y. Iseki, M. Wada, *Tetrahedron Lett.* **1982**, *23*, 3935–3936.

[9] T. Kaminski, P. Gros, Y. Fort, *European J. Org. Chem.* **2003**, *2003*, 3855–3860.

[10] L. G. Vaughan, W. A. Sheppard, *J. Am. Chem. Soc.* **1970**, *11*, 730–731.

[11] P. Thordarson, *Chem. Soc. Rev.* **2011**, *40*, 1305–1323.

[12] S. Grimme, *Chem. Eur. J.* **2012**, *18*, 9955–9964.

[13] S. Grimme, J. G. Brandenburg, C. Bannwarth, A. Hansen, *J. Chem. Phys.* **2015**, *143*, 54107.

[14] F. Weigend, R. Ahlrichs, *Phys. Chem. Chem. Phys.* **2005**, *7*, 3297–3305.

[15] S. Grimme, J. Antony, S. Ehrlich, H. Krieg, *J. Chem. Phys.* **2010**, *132*, 154104.

[16] H. Kruse, S. Grimme, *J. Chem. Phys.* **2012**, *1336*, 154101.

[17] C. Adamo, V. Barone, *J. Chem. Phys.* **1999**, *110*, 6158.

[18] M. Ernzerhof, G. E. Scuseria, *J. Chem. Phys.* **1999**, *110*, 5029.

[19] T. Risthaus, S. Grimme, *J. Chem. Theory Comput.* **2013**, *9*, 1580–1591.

[20] E. R. Johnson, A. D. Becke, *J. Chem. Phys.* **2005**, *123*, 24101.

[21] A. D. Becke, E. R. Johnson, *J. Chem. Phys.* **2005**, *123*, 154101.

[22] B. M. Axilrod, E. Teller, *J. Chem. Phys.* **1943**, *11*, 299.

[23] Y. Muto, *Proc. Phys. Math. Soc. Jpn.* **1944**, *17*, 629.

[24] S. Grimme, C. Bannwarth, P. Shushkov, *J. Chem. Theory Comput.* **2017**, *13*, 1989–2009.

[25] A. Klamt, *J. Phys. Chem.* **1995**, *99*, 2224–2235.

[26] F. Eckert, A. Klamt, *AIChE J* **2002**, *48*, 369–385.

[27] F. Eckert, A. Klamt, COSMOtherm, Version C3.0, Release 16.01; COSMOlogic GmbH & Co. KG, Leverkusen, Germany, **2016**.

[28] F. Furche, R. Ahlrichs, C. Hättig, W. Klopper, M. Sierka, F. Weigend, *WIREs Comput. Mol. Sci.* **2014**, *4*, 91–100.

[29] see also: Turbomole.com**.**

[30] K. Eichkorn, O. Treutler, H. Öhm, M. Häser, R. Ahlrichs, *Chem. Phys. Lett.* **1995**, *242*, 652–660.

[31] F. Weigend, *Phys. Chem. Chem. Phys.* **2006**, *8*, 1057–1065.

[32] Y. Zhao, D. G. Truhlar, *J. Phys. Chem. A.* **2005**, *109*, 5656–5667.

[33] J. P. Perdew, *Phys. Rev. B* **1986**, *33*, 8822–8824.

[34] A. D. Becke, *Phys. Rev. A.* **1988**, *38*, 3098–3100.

[35] E. F. Pettersen, T. D. Goddard, C. C. Huang, G. S. Couch, D. M. Greenblatt, E. C. Meng, T. E. Ferrin, *J. Comput. Chem.* **2004**, *25*, 1605–1612.

[36] C. Riplinger, B. Sandhoefer, A. Hansen, F. Neese, *J. Chem. Phys.* **2013**, *139*, 134101.

[37] C. Riplinger, P. Pinski, U. Becker, E. F. Valeev, F. Neese, *J. Chem. Phys.* **2016**, *144*, 24109.

[38] ORCA – an ab initio, density functional and semiempirical program package, V. 4.0.0, F. Neese, MPI für Chemische Energiekonversion, Mülheim a. d. Ruhr (Germany), **2017.**

[39] F. Neese, *Comput. Mol. Sci.* **2012**, *2*, 73–78.

[40] A. Hellweg, C. Hattig, S. Hofener, W. Klopper, *Theor. Chem. Acc.* **2007**, *117*, 587–597.

[41] D. Andrae, U. Haeussermann, M. Dolg, H. Stoll, H. Preuss, *Theor. Chim. Acta* **1990**, *77*, 123–141.

[42] F. Neese, E. F. Valeev, *J. Chem. Theory Comput.* **2011**, *7*, 33.

[43] E. Caldeweyher, C. Bannwarth, S. Grimme, *J. Chem. Phys.* **2017**, *147*, 34112.

[44] J. P. Perdew, K. Burke, M. Ernzerhof, *Phys. Rev. Lett.* **1996**, *77*, 3865–3868.
